# The Promoting Role of Ni on In_2_O_3_ for CO_2_ Hydrogenation to Methanol

**DOI:** 10.1021/acscatal.2c04872

**Published:** 2023-01-18

**Authors:** Francesco Cannizzaro, Emiel J. M. Hensen, Ivo A. W. Filot

**Affiliations:** Laboratory of Inorganic Materials and Catalysis, Department of Chemical Engineering and Chemistry, Eindhoven University of Technology, 5600 MBEindhoven, The Netherlands

**Keywords:** CO_2_ hydrogenation, In_2_O_3_, Ni, mechanism, oxygen vacancy

## Abstract

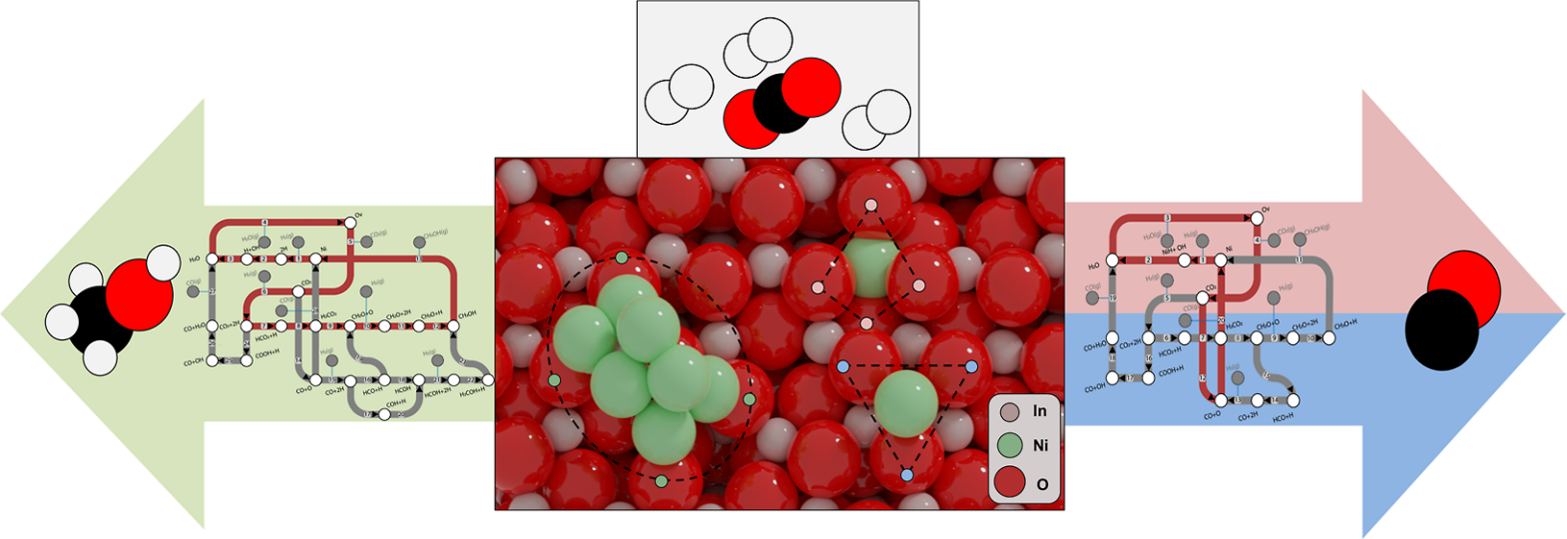

Ni-promoted indium oxide (In_2_O_3_) is a promising catalyst for the selective hydrogenation of CO_2_ to CH_3_OH, but the nature of the active Ni sites remains unknown. By employing density functional theory and microkinetic modeling, we elucidate the promoting role of Ni in In_2_O_3_-catalyzed CO_2_ hydrogenation. Three representative models have been investigated: (i) a single Ni atom doped in the In_2_O_3_(111) surface, (ii) a Ni atom adsorbed on In_2_O_3_(111), and (iii) a small cluster of eight Ni atoms adsorbed on In_2_O_3_(111). Genetic algorithms (GAs) are used to identify the optimum structure of the Ni_8_ clusters on the In_2_O_3_ surface. Compared to the pristine In_2_O_3_(111) surface, the Ni_8_-cluster model offers a lower overall barrier to oxygen vacancy formation, whereas, on both single-atom models, higher overall barriers are found. Microkinetic simulations reveal that only the supported Ni_8_ cluster can lead to high methanol selectivity, whereas single Ni atoms either doped in or adsorbed on the In_2_O_3_ surface mainly catalyze CO formation. Hydride species obtained by facile H_2_ dissociation on the Ni_8_ cluster are involved in the hydrogenation of adsorbed CO_2_ to formate intermediates and methanol. At higher temperatures, the decreasing hydride coverage shifts the selectivity to CO. On the Ni_8_-cluster model, the formation of methane is inhibited by high barriers associated with either direct or H-assisted CO activation. A comparison with a smaller Ni_6_ cluster also obtained with GAs exhibits similar barriers for key rate-limiting steps for the formation of CO, CH_4_, and CH_3_OH. Further microkinetic simulations show that this model also has appreciable selectivity to methanol at low temperatures. The formation of CO over single Ni atoms either doped in or adsorbed on the In_2_O_3_ surface takes place via a redox pathway involving the formation of oxygen vacancies and direct CO_2_ dissociation.

## 1 Introduction

Anthropogenic CO_2_ emissions due to combustion of fossil fuels constitute a serious environmental threat because of their negative impact on the climate such as global warming, rise in sea level, and ocean acidification.^1−3^ One of the most attractive strategies to reduce these emissions is to close the carbon cycle by recycling CO_2_ captured from combustion processes or directly from the air, followed by its conversion into chemical feedstocks and fuels. This can be achieved by converting CO_2_ with hydrogen obtained from renewable resources.^4−6^ The use of carbon in a circular manner can eventually lead to the replacement of fossil fuels with renewable resources for covering the energy demand. Besides effectively mitigating emissions, CO_2_ hydrogenation products can be used to synthesize important chemical feedstocks. In the overall context of sustainability, methanol is particularly attractive because it can be directly used as a fuel and a building block to produce a wide range of chemicals such as formaldehyde, dimethyl ether, olefins, and hydrocarbon fuels.^7−9^

Currently, large-scale methanol production is based on the conversion of synthesis gas (CO/CO_2_/H_2_) over Cu/ZnO/Al_2_O_3_ catalysts at typical temperatures and pressures of 473–573 K and 50–100 bar, respectively.^10,11^ Challenges arise, however, in the hydrogenation of CO_2_ to CH_3_OH. The commercial Cu-based catalyst shows significant activity for the reverse water–gas-shift reaction (rWGS), leading to the formation of unwanted CO.^12^ Moreover, the catalyst is prone to sintering, decreasing the activity.^13,14^ Many efforts have been made to identify other catalyst formulations more suitable for the hydrogenation of CO_2_ to CH_3_OH.^15−21^ Among these, indium oxide (In_2_O_3_) was recently introduced as a promising catalyst for CO_2_ hydrogenation to methanol, especially when supported on ZrO_2_.^22−25^ Several density functional theory (DFT) studies emphasize the role of oxygen vacancies in the mechanism of CO_2_ hydrogenation to methanol on In_2_O_3_.^26−31^ Although In_2_O_3_ enables high selectivity toward methanol by suppressing the competitive rWGS reaction, CO_2_ conversion is limited by the low activity of indium oxide in dissociating molecular H_2_. To enhance the rate of hydrogen activation, several metal promoters have been investigated. Rui et al. showed that the addition of small Pd nanoparticles can enhance methanol synthesis.^32^ Supporting DFT calculations indicated that small Pd clusters increase the rate of H_2_ activation, resulting in a larger amount of H atoms at the metal–oxide interface.^33^ Frei et al. proposed that single-atom (SA) Pd doped inside the In_2_O_3_ lattice can stabilize clusters of a few Pd atoms on the In_2_O_3_ surface, enhancing H_2_ activation and, therefore, CH_3_OH productivity.^25^ Similar results have been reported for Pt-promoted In_2_O_3_.^34−36^ Given their price, it would be advantageous to replace Pd and Pt with earth-abundant metals like first-row transition metals. Earlier investigations have shown that adding Co^37^ and Cu^38^ to In_2_O_3_ can enhance the activity of In_2_O_3_ for methanol synthesis. More recently, Jia et al. prepared a highly dispersed Ni/In_2_O_3_ catalyst with significantly enhanced CO_2_ conversion compared to the unpromoted oxide while preserving high methanol selectivity.^39^ Notably, no methane was found in the product stream. In another study, Snider et al. suggested that the higher activity of bimetallic Ni–In catalysts for CO_2_ hydrogenation to CH_3_OH compared to In_2_O_3_ is due to the synergistic interactions between a Ni–In alloy and In_2_O_3_.^40^ Furthermore, Frei et al. reported that highly dispersed InNi_3_ patches formed on the In_2_O_3_ surface increase methanol production by supplying neutral H species, whereas Ni SAs were active for the rWGS.^41^ Shen et al. investigated the DFT pathways of CO_2_ hydrogenation to methanol on a Ni_4_/In_2_O_3_ model catalyst, demonstrating that oxygen vacancies are involved in CO_2_ hydrogenation, followed by hydrogenation of adsorbed CO_2_ to methanol.^42^ In a recent experimental study, we showed that Ni/In_2_O_3_ catalysts prepared using flame spray pyrolysis synthesis have enhanced CH_3_OH synthesis activity compared to that of bare In_2_O_3_.^43^ X-ray photoelectron spectroscopy, X-ray absorption spectroscopy, and electron paramagnetic resonance analyses of the used catalysts suggest that the working state of the catalyst corresponds to SAs or clusters of a few atoms of Ni dispersed on the In_2_O_3_ surface. However, differentiating the catalytic role of single Ni atoms and small clusters was challenging, meaning that the exact nature of Ni species in the active sites of Ni/In_2_O_3_ catalysts remains unclear. Supporting DFT calculations indicate that Ni promotion of methanol synthesis from CO_2_ on In_2_O_3_ is mainly due to low-barrier H_2_ dissociation, yet the mechanism of CO_2_ conversion to methanol has not been explored so far.

In the present study, we employ DFT in combination with genetic algorithms (GAs) and microkinetic modeling to systematically investigate the nature of the active sites and the mechanism of CO_2_ hydrogenation to methanol on Ni/In_2_O_3_. To understand the nature of the active site, we consider the following three model systems: (i) SA doped in and (ii) SA adsorbed on In_2_O_3_(111) and (iii) a Ni_8_ cluster placed on a In_2_O_3_(111) surface. GAs are used to identify optimum structural models for supported clusters, while DFT calculations are used to determine the elementary reaction steps for CO_2_ conversion to CH_3_OH, CO, and H_2_O. On the basis of these first-principles data, we construct microkinetic models to predict the CO_2_ consumption rate and the product distribution as a function of temperature, as well as surface coverages, reaction orders, and apparent activation energies. We perform a sensitivity analysis of the kinetic network to identify the elementary steps that control the rate of CO_2_ consumption and CH_3_OH selectivity. We herein show the factors underlying different activity and selectivity patterns on Ni/In_2_O_3_ model catalysts during CH_3_OH synthesis from CO_2_ hydrogenation, highlighting that small clusters are the most active and selective form of Ni for obtaining the desired methanol product, whereas on Ni SA models, the competing rWGS is preferred, wherein CO_2_ is hydrogenated to CO and H_2_O.

## 2 Computational Methods

### 2.1 DFT Calculations

All DFT calculations were conducted using the projector augmented wave (PAW) method^44^ and the Perdew–Burke–Ernzerhof^45^ exchange–correlation functional, as implemented in the Vienna ab initio simulation package (VASP) code.^46,47^ Solutions to the Kohn–Sham equations were calculated using a plane-wave basis set with a cut-off energy of 400 eV. The semi-core 5s and 5p states of In were treated explicitly as valence states within the scalar-relativistic PAW approach. All calculations were spin-polarized. The Brillouin zone was sampled using a 3 × 3 × 1 Monkhorst–Pack grid. Electron smearing was employed using Gaussian smearing with a smearing width (σ) of 0.1 eV. We chose the In_2_O_3_(111) surface termination because it is more stable than the (110) and (100) surfaces, as shown in a previous computational study.^48^ The stoichiometric In_2_O_3_(111) surface was modeled as a 2D slab with periodic boundary conditions. A 20.0 Å vacuum region was introduced in the *c*-direction to avoid the spurious interaction with neighboring super cells. It was verified that the electron density approached zero at the edges of the periodic super cell in the *c*-direction. In all calculations, the bottom two layers were frozen, while the top two layers were allowed to perturb. The supercell has dimensions of 14.57 Å × 14.57 Å × 26.01 Å. The In_2_O_3_(111) slab consisted of 96 O atoms and 64 In atoms distributed in four atomic layers on top of which the Ni species were placed.

The global minimum (GM) structure of In_2_O_3_-supported Ni clusters of either 6 or 8 atoms (Ni_6_ and Ni_8_) was determined by an in-house written GA procedure based on the earlier approach of Hammer et al.^49,50^ In this procedure, evolutionary processes including cross-over and mutation were employed to generate new candidate structures, whose geometry was optimized using the conventional conjugate-gradient method. This resulted in increasingly more stable clusters while maintaining a statistically diverse population to ensure that sufficiently distinct new candidates could be generated. The algorithm was seeded using 12 randomly generated clusters. Each iteration of the algorithm produced a new generation of clusters until population stagnation was observed, that is until no new clusters stabler than the existing clusters in the population pool were found. For the Ni_8_-cluster, a total of 513 structures were generated, of which the 201 most stable clusters were selected for further analysis. For the Ni_6_-cluster, 136 stable candidates were identified out of 346 structures obtained in total. The structure with the lowest energy in the pool of candidates was designated as the GM structure. To better understand the structures obtained with the GA, a statistical analysis based on Boltzmann statistics and a similarity analysis based on the minimum Hilbert–Schmidt (HS) norm were employed. In the latter, a distance matrix for each cluster was produced, wherein each matrix element represents the distance between any two atoms in a single cluster. The similarity between any two clusters in the set can then be expressed as the minimum HS norm of the difference of their distance matrices, wherein the minimum is established by evaluating all possible permutations over the indices for one of the distance matrices.

The influence of oxygen vacancies on the reaction energetics was investigated by removing oxygen atoms from the In_2_O_3_(111) lattice. The energy required to remove surface oxygen to form a vacancy (Δ*E*_Ov_) was calculated using either O_2_ or H_2_O as the reference, according to the following two equations for the oxygen vacancy formation energy
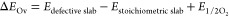
1
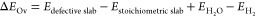
2Where *E*_defective slab_ is the electronic energy of the catalyst containing one oxygen vacancy, and *E*_stoichiometric slab_ is the reference energy of the stoichiometric slab. , , and  are the DFT-calculated energies of gas-phase O_2_, H_2_O, and H_2_, respectively. Herein, we include the electronic energy, the zero-point energy (ZPE) correction, and a finite temperature correction of translational and rotational energies of each gas-phase molecule.

The stable states in the chemo-kinetic network were calculated using the conjugate-gradient algorithm. Transition states were determined using the climbing-image nudged elastic band method.^51^ A frequency analysis was performed to all states. Specifically, it was verified that stable states have no imaginary frequencies and transition states have a single imaginary frequency in the direction of the reaction coordinate.^52^ The Hessian matrix in this frequency analysis was constructed using a finite difference approach with a step size of 0.0015 Å for the displacement of individual atoms along each Cartesian coordinate. The corresponding normal mode vibrations were also used to calculate the ZPE correction and the vibrational partition functions.

Partial density of state (pDOS) and projected crystal orbital Hamiltonian population (pCOHP) analyses are conducted to analyze the electronic structure of each Ni/In_2_O_3_ model catalyst using the Lobster package.^53,54^ The atomic charges of Ni atoms were calculated using the Bader charge method.^55^

### 2.2 Microkinetic Simulations

Microkinetic simulations were conducted based on the DFT-calculated activation barriers and reaction energies to investigate the kinetics of CO_2_ hydrogenation to methanol. The chemo-kinetic network was modeled using a set of ordinary differential equations involving rate constants, surface coverages, and partial pressures of gas-phase species. Time-integration of the differential equations was conducted using the linear multistep backward differential formula method with a relative and absolute tolerance of 10^–8^.^56−58^

For the adsorption processes, the net rate of a gas-phase species *i* was calculated as
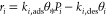
3where θ* and θ_*i*_ are the fraction of free sites and the fraction of coverage species *i*, respectively. *k*_*i*,ads/des_ is the rate constant for the adsorption/desorption process, and *P*_*i*_ is the partial pressure of species *i*.

To derive a rate for the adsorption processes, we assumed that the adsorbate loses one translational degree of freedom in the transition state with respect to the initial state. From this assumption, the rate of adsorption derived from transition state theory can be expressed as follows
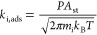
4where *A*_st_ and *m*_*i*_ are the effective area of an adsorption site and the molar mass of the gas species, respectively. *P* and *T* are the total pressure and temperature, respectively, and *k*_B_ is the Boltzmann constant. The gas-phase entropy of the adsorbates was calculated using the thermochemical Shomate equation as given by
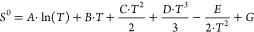
5where *S*^0^ is the standard molar entropy.^59^ The parameters A–G from eq 5 were obtained from the NIST Chemistry Webbook.^60^ For the corresponding desorption processes, we assumed that the species gains two translational degrees of freedom and three rotational degrees of freedom in the transition state with respect to the initial state. From this assumption, the rate of desorption derived from transition state theory can be expressed as follows
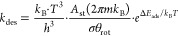
6Herein, *k*_des_ is the rate constant for the desorption of the adsorbate, *h* is the Planck constant, σ is the symmetry number and is equal to 1, θ_rot_ is the rotational temperature, and Δ*E*_ads_ is the desorption energy. The value of A_st_ is equal to 9 × 10^–19^ m^–2^.

Finally, the rate constant (*k*) of an elementary reaction step is given by
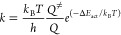
7where *Q*^≠^ and *Q* are the partition functions of the activated complex and its corresponding initial state, respectively, and Δ*E*_act_ is the ZPE-corrected activation energy.

To identify the steps that control the CO_2_ consumption rate and the product distribution, we employed the concepts of the degree of rate control (DRC) developed by Kozuch and Shaik^61,62^ and popularized by Campbell^63^ as well as the degree of selectivity control (DSC).^63−65^

Herein, the DRC coefficient is defined as
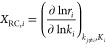
8

A positive DRC coefficient indicates that the elementary reaction step is rate-controlling, whereas a negative coefficient suggests that the step is rate-inhibiting. When a single elementary reaction step has a DRC coefficient of 1, this step is identified as the rate-determining step.

The DSC quantifies the extent to which a particular elementary reaction step influences the selectivity for certain products. The DSC of a particular key component is expressed as
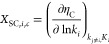
9where *X*_SC,*i*,*c*_ is the DSC of product *c* due to a change in the kinetics of the elementary reaction step *i*, and η_C_ is the selectivity toward a key product (methanol in this work). Note that the relationship between DRC and DSC coefficients is given by
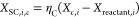
10

## 3 Results and Discussion

### 3.1 Structure of Ni/In_2_O_3_ Models

**Figure 1:**
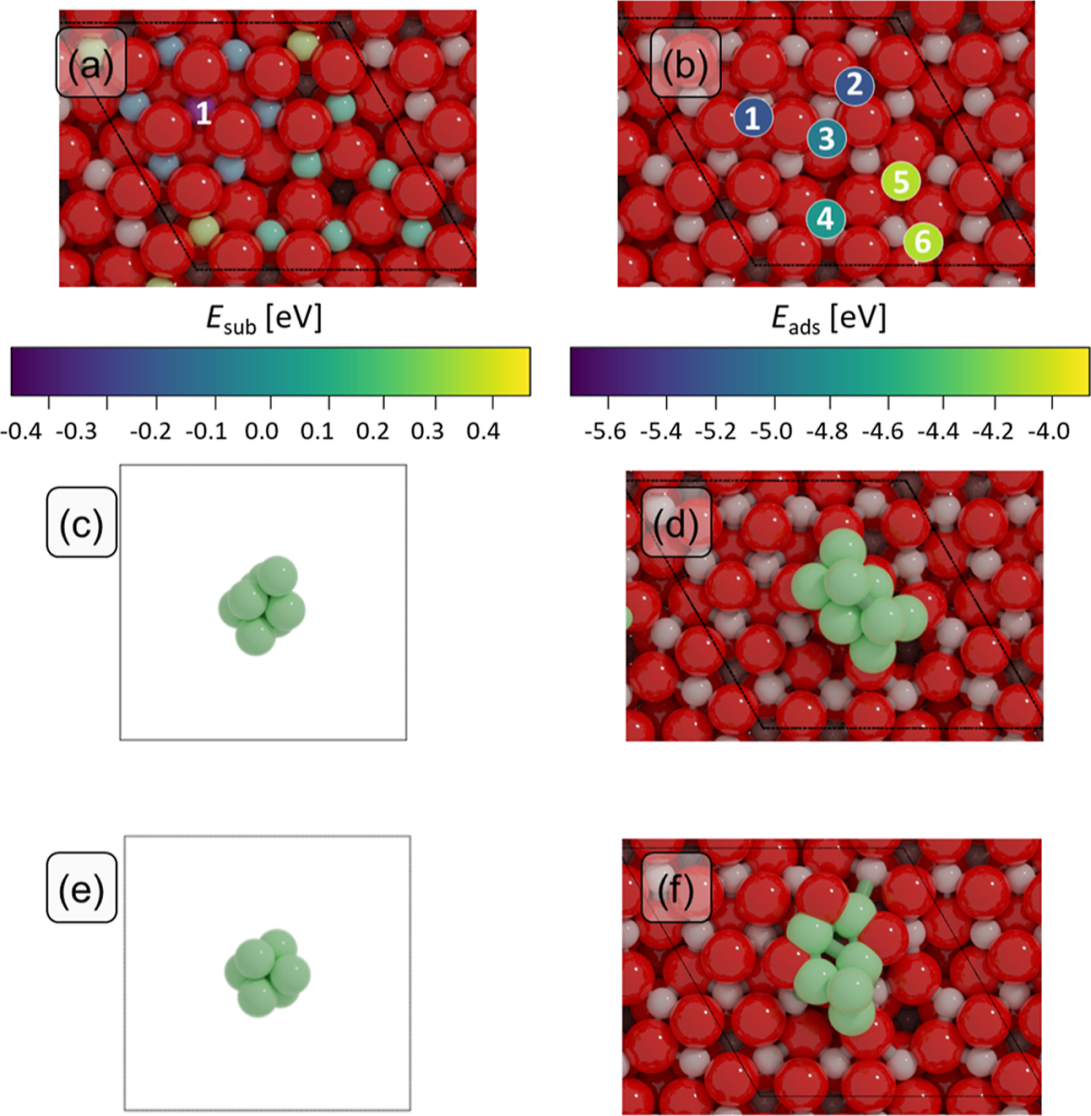
(a) Substitution energy of In/Ni substitution in In_2_O_3_. The coloring of In atoms at the surface represents the energy associated with their replacement by Ni (*E*_sub_). Position 1 marks the most stable substitution site. (b) Adsorption energy of Ni SAs on In_2_O_3_(111). The coloring of the atoms at the surface represents their adsorption energy (*E*_ads_). Position 1 marks the most stable adsorption site. Energy minima structures obtained by the GA for gas-phase (c,e) and supported (d,f) Ni_8_ and Ni_6_ clusters, respectively. Red: O; gray: In; green: Ni.

The understand the nature of the active sites for Ni/In_2_O_3_ in CO_2_ hydrogenation to methanol, three model systems were considered, namely SA of Ni either (i) doped in the In_2_O_3_(111) surface (denoted as Ni_1_-doped) or (ii) adsorbed on the In_2_O_3_(111) surface (Ni_1_-adsorbed) and a cluster of (iii) 8 Ni atoms adsorbed on top of the In_2_O_3_(111) surface (Ni_8_-cluster). The models are shown in Figure 1. We also compare our Ni_8_ cluster with a smaller Ni_6_ cluster obtained at the same level of theory. To determine the most stable location of Ni for the Ni_1_-doped In_2_O_3_(111) model system, the energy of substituting an In atom for a Ni atom was calculated. The reported substitution energies (*E*_sub_) are given with respect to bulk Ni and In by
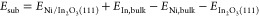
11where  is the energy of each Ni_1_-doped In_2_O_3_(111) surface, *E*_In,bulk_, *E*_Ni,bulk,_ and  are the energies of In bulk, Ni bulk, and In_2_O_3_(111) surface models, respectively. The resulting substitution energies for all the doping positions are collected in Table S1. DFT calculations indicate that Ni substitution is favored in position 1 as shown in Figure 1a (*E*_sub_ = −0.35 eV). Ni prefers a nearly perfect octahedral coordination by oxygen in the first metal layer of the structure (position 1). The six sites nearest to position 1 are slightly less stable (i.e., with substitution energies ∼+0.15 eV compared to position 1) due to a slight distortion of the octahedral environment around the Ni substituent. Compared to these sites, substitution at other positions in the first metal layer is unfavorable, with substantially larger exchange energies. We also studied the possibility of substituting an In atom in the bulk of In_2_O_3_ with a Ni atom (Figure S2a). Our calculations indicate that the substitution of a Ni SA in subsurface layers is unfavorable (*E*_sub_ = +0.51 eV). We also evaluated the possibility of replacing a second In atom in the surface (Figure S2b) and found that this is also unfavorable (*E*_sub_ = +0.59 eV). These findings are in line with a previous study on Pd-doped In_2_O_3_.^25^

The preferred location of a Ni SA on the In_2_O_3_(111) surface is bridging between two lattice oxygens (Figure 1b). The adsorption energies for all investigated sites are reported in Table S2. In its most stable configuration, the Ni–O distances are *r*(Ni–O_α_) = 1.79 Å and *r*(Ni–O_β_) = 1.81 Å (Figures 1b and S3b). In its least stable adsorption configuration (position 6), Ni coordinates to a single oxygen atom and an In atom. The Ni–O and Ni–In distances are 2.14 and 2.54 Å, respectively. The Ni–O distance increases from 1.79 to 2.14 Å from the most stable to the least stable adsorption configuration, in line with a weaker binding of the Ni SA with the surface. In general, more stable configurations feature multiple Ni–O bonds. Furthermore, we determined barriers for the diffusion of a single Ni atom between stable adsorption sites (Table S3). Migration from position 1 to position 3 (Figure 1b) is associated with a barrier of 95 kJ/mol and is endothermic by 21 kJ/mol. Migration from position 3 to position 4 has a higher barrier (165 kJ/mol) and is endothermic by 11 kJ/mol. Lastly, migration from position 4 to position 5 is associated with a barrier of 142 kJ/mol and is endothermic by 65 kJ/mol.

In a recent experimental study on Ni/In_2_O_3_, it was found that Ni is present in a clustered form under reducing reaction conditions.^43^ These clusters were relatively small in optimized catalysts, as no methane was observed during CO_2_ hydrogenation. Methane was only formed at much higher Ni loading, where Ni nanoparticles were present on In_2_O_3_. To model small Ni clusters, we used clusters containing either 8 or 6 Ni atoms (Ni_8_ and Ni_6_, respectively) on the In_2_O_3_(111) surface as a compromise between computational tractability and a reasonable description of supported metal clusters that often adopt a bilayer structure.^49^ For comparison, we also determined the most stable structure of clusters in the gas phase of the Ni_8_ and Ni_6_ clusters (Figure 1c,e, respectively). The DFT-based GA identified 201 and 136 stable candidate structures for the Ni_8_ and Ni_6_-cluster models, respectively. The algorithm did not find new configurations that were significantly different from the energy minimum within an energy threshold of 0.1 eV after 57 and 36 genetic iterations for the Ni_8_ and Ni_6_-cluster models, respectively. The minimum-energy structures for gas-phase and supported Ni_8_ and Ni_6_ clusters are shown in Figure 1c–f respectively. In the gas phase, the most stable free clusters adopt a square bipyramidal shape, in line with an earlier computational study.^66^ The most stable supported Ni_8_ cluster obtained by our DFT-GA (Figure 1d) consists of a bilayer structure where 6 Ni atoms form the bottom layer, with the other two Ni atoms placed on threefold sites of the first Ni layer. For the supported Ni_6_ cluster (Figure 1f), 5 Ni atoms form the bottom layer, and the sixth Ni atom is placed on a threefold site. The different shapes of the Ni clusters in the gas phase and on the In_2_O_3_(111) support are caused by strong metal-support interactions (MSI) between Ni and In_2_O_3_. Such MSI were quantified by computing the cohesive energy per Ni atom of the GA-optimized Ni_8_ clusters in the gas phase and supported on In_2_O_3_ (Table S4). Placing the Ni_8_ cluster on the (111) surface of In_2_O_3_ increases the cohesive energy from 268 kJ/mol/atom for the gas-phase cluster to 358 kJ/mol/atom for the cluster on the In_2_O_3_ support. For a Ni_6_-cluster, the stabilization effect is slightly higher (111 kJ/mol/atom).

The stability of the supported Ni clusters can be further evaluated by determining the barrier needed to remove a single Ni atom from the cluster via migration on the In_2_O_3_ surface. We performed such DFT calculations and reported the results in Tables S5 and S6. Removing a Ni atom from a Ni_6_ cluster has a barrier of 201 kJ/mol and is endothermic by 179 kJ/mol. For the Ni_8_-cluster, the corresponding values are 232 and 192 kJ/mol. Thus, it is clear that the redispersion of Ni clusters into SAs is prohibitive from the kinetic and thermodynamic points of view.

To determine whether other configurations of the Ni clusters will also contribute to the catalytic performance, we analyzed the Boltzmann probability distribution for the structures (Section S3.2 of the Supporting Information). This analysis demonstrated that, for both clusters, one other structure occurs with a sufficiently large contribution at a reaction temperature at 400 K. Based on a structural similarity analysis (Table S7), it was found that this structure is nearly the same as the minimum energy structure for both the Ni_6_ and Ni_8_-cluster models. Accordingly, we focused only on the minimum energy structure of the Ni_8_ cluster in the following.

The electronic structure of the Ni/In_2_O_3_ models can be compared on the basis of the pDOS (Figure S6). In all cases, overlap between Ni 3d and O 2p orbitals is observed. The In 4d orbitals are core orbitals and, therefore, do not show appreciable overlap with O 2p or Ni 3d orbitals. For optimized, doped, and adsorbed SA models, the calculated Bader charges of the Ni atoms are +1.26 and +0.66 |e|, respectively. The Bader charge analysis further shows that Ni_8_ clusters on In_2_O_3_(111) carry a cumulative charge of +1.07 |e| (Table S8 and Figure S7a). The six Ni atoms located at the bottom layer of the cluster carry a positive charge, while the two Ni atoms adsorbed on top carry a slightly negative charge. Thus, for all models, there is a net flow of electrons from Ni to the O atoms of the In_2_O_3_ surface.

### 3.2 Oxygen Vacancy Formation

**Figure 2:**
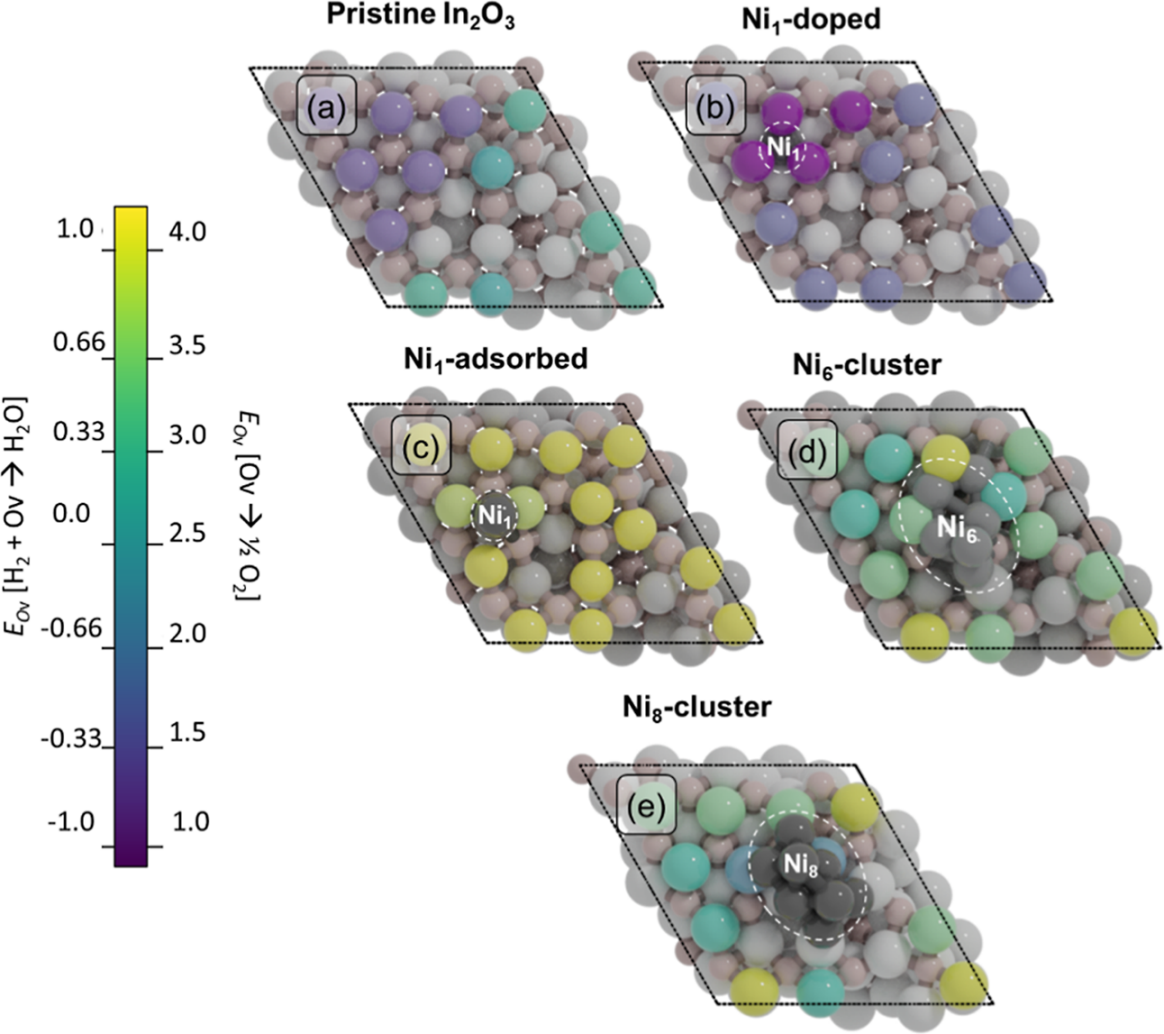
Oxygen vacancy formation energies (*E*_Ov_) for the 12 surface oxygens on (a) bare In_2_O_3_, (b) Ni_1_-doped in and (c) Ni_1_-adsorbed on In_2_O_3_, (d) Ni_6_-cluster, and (e) Ni_8_-cluster. The coloring of the 12 surface oxygen atoms represents their *E*_Ov_. Ni atoms are colored in dark gray, while all other atoms are colored in light gray. The Ni species are highlighted inside dashed circles.

The role of oxygen vacancies (Ov) on the In_2_O_3_ surface has been emphasized for the adsorption and activation of CO_2_.^26−28^ To assess how the addition of Ni affects the formation of such vacancies, we compared the energy required to form such Ov (*E*_Ov_, in eV) for the Ni/In_2_O_3_ surfaces with unpromoted In_2_O_3_ (Figure 2). The computed energies are referenced to gaseous O_2_. On the bare In_2_O_3_(111) surface (Figure 2a), the energy needed to remove an oxygen atom ranges from 1.8 to 3.0 eV, in line with previous calculations.^48,67^ We also calculated the Ov formation energies with respect to H_2_O because oxygen vacancies are usually formed in the presence of H_2_. In this case, the *E*_Ov_ lies between −0.65 and 0.99 eV.

Doping In_2_O_3_ with a Ni SA (Figure 2b) lowers the energy for Ov formation significantly. Values for *E*_Ov_ referenced to O_2_ lie between 0.94 and 1.86 eV. Considering H_2_O formation, oxygen vacancy formation is exothermic for all surface oxygens considered (*E*_Ov_ between −1.58 and −0.66 eV). As can be seen from Figure 2b, the O atoms bonded to Ni are more easily removed than O atoms bonded to In. In its most stable doping position, the Ni–O bond distance is 1.94 Å, which is 0.25 Å shorter than the In–O distance measured for an In atom occupying the same lattice position on the bare oxide. This suggests that doping a smaller Ni atom in In_2_O_3_ leads to lattice contraction bringing the O atoms closer to each other. This enhances electron–electron repulsion between the relatively large electron clouds around the negatively charged O atoms, weakening the Ni–O bonds, and thereby explaining the lower *E*_Ov_. Adsorbing a Ni atom on top of the In_2_O_3_ (Figure 2c) significantly increases the oxygen vacancy formation energy, resulting in *E*_Ov_ values between 3.42 and 4.41 eV referenced to O_2_ and between 0.89 and 1.87 eV referenced to H_2_O. For a supported Ni_6_ cluster (Figure 2d), oxygen vacancy formation via direct thermal desorption of O_2_ lies between 2.65 and 3.52 eV. With respect to H_2_O formation, *E*_Ov_ values lie between 0.12 and 1.16 eV. For a supported Ni_8_ cluster (Figure 2e), similar values are found for oxygen vacancy formation energies via direct thermal desorption (2.48–3.51 eV). Thus, adsorbed Ni phases increase the oxygen vacancy formation energy compared to the reference pristine In_2_O_3_ case. This is in line with our knowledge that the Ni–O bond (396 kJ/mol) is stronger than the In–O bond (346 kJ/mol).^68^

A more careful inspection of Figure 2b–d shows that, for each of the Ni-promoted surfaces, formation of vacancies is easier for oxygens directly bonding to Ni species than for O atoms bonding only to In atoms. To understand this point, we performed a pCOHP analysis of the Ni–O and In–O bonds (Figure S8), where we compare the stoichiometric In_2_O_3_ surface with Ni-promoted ones. On In_2_O_3_ (Figure S8a), the character of In–O interactions is bonding. For all Ni-promoted surface models (Figure S8b–i), the pCOHP exhibits antibonding Ni–O and In–O interactions close to the Fermi level for oxygen atoms adjacent to Ni. This explains why less energy is needed to remove these oxygens compared to oxygens at larger distances from Ni. We infer that the addition of Ni to In_2_O_3_ causes electron transfer from Ni to O, which results in the formation of antibonding N–O and In–O interactions. Furthermore, Bader charge analysis shows that, upon formation of an oxygen vacancy, the excess charge is redistributed mainly to the nearest Ni atom from the cluster (Figure S7b). In this way, Ni atoms can contribute to stabilizing the oxygen-defective Ni/In_2_O_3_ surface, resulting in easier formation of vacancies at the Ni/In_2_O_3_ interface. In summary, with respect to the stoichiometric In_2_O_3_ surface, only the Ni_1_-doped model features more exothermic oxygen vacancy formation energies, whereas the other Ni/In_2_O_3_ models feature more endothermic oxygen vacancy formation energies. To understand whether oxygen vacancies will be formed on these models, we investigate the DFT pathways of oxygen vacancy formation via surface reduction by H_2_ and perform microkinetic simulations (vide infra).

### 3.3 Elementary Reaction Steps

**Figure 3:**
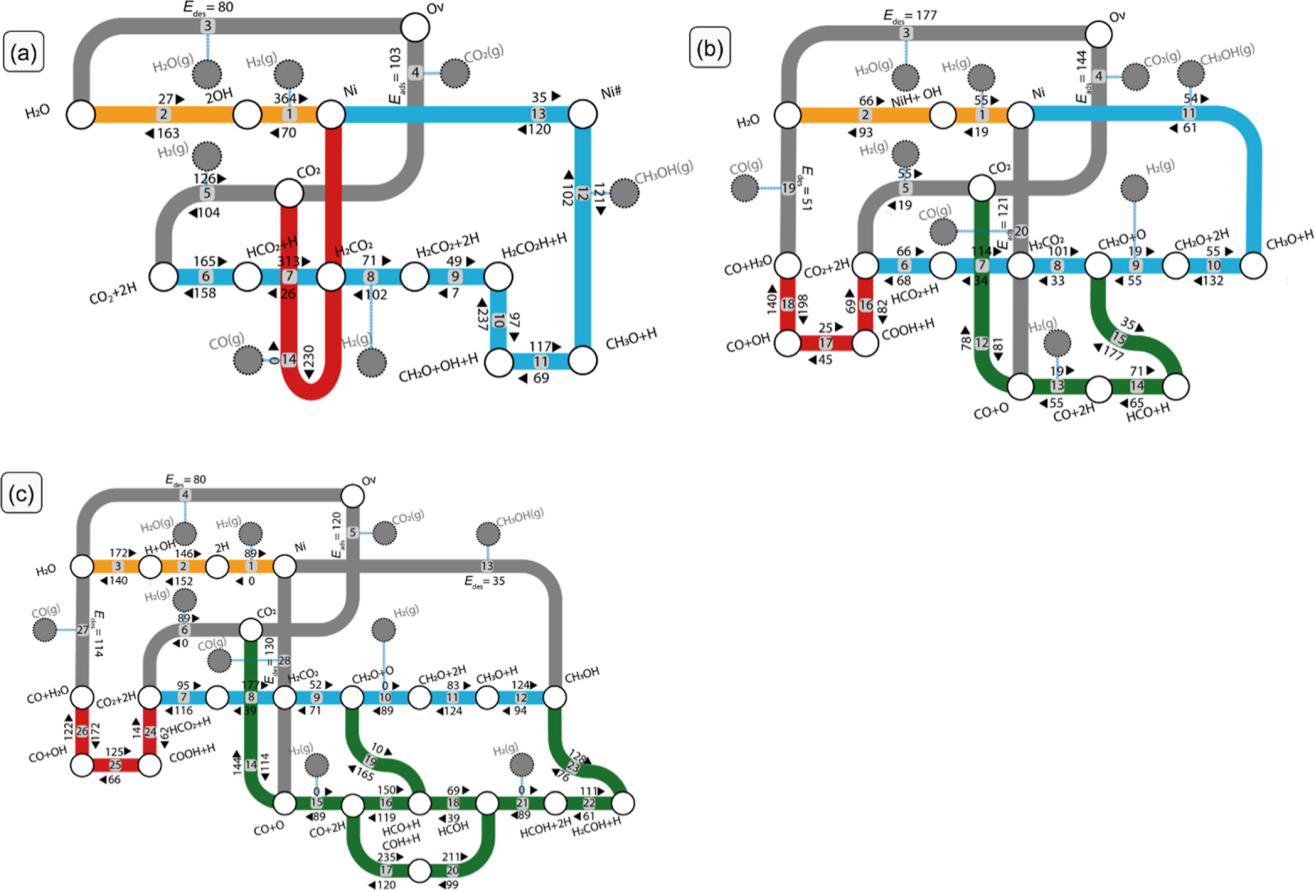
Full kinetic networks for CO_2_ hydrogenation to CO and CH_3_OH for (a) Ni_1_-doped, (b) Ni_1_-adsorbed, and (c) Ni_8_-cluster models. Orange: oxygen vacancy formation pathway; blue: formate pathway to CH_3_OH; green: CO hydrogenation pathway to CH_3_OH; red: rWGS to CO; gray: adsorption/desorption elementary steps. The numbers correspond to the elementary reaction steps as listed in Tables S9a–c.

We performed DFT calculations to elucidate the reaction mechanism of CO_2_ hydrogenation to methanol (CH_3_OH), carbon monoxide (CO), and water (H_2_O). No methane is observed during CO_2_ hydrogenation experiments for a Ni/In_2_O_3_ catalyst with a low Ni loading.^32,34^ To verify that CO_2_ methanation on small In_2_O_3_-supported Ni clusters is difficult, we studied direct and H-assisted CO dissociation to assess whether the second C–O bond scission is facile. The reaction network explored in this study is depicted in Figure 3. Based on previous computational studies, we investigate specific pathways for the formation of oxygen vacancies via H_2_O, for the formation of CH_3_OH via formate, and for the rWGS pathway leading to CO.^24,70,71^ The latter can take place either via direct cleavage of one C–O bond in CO_2_ or via an H-assisted pathway involving the COOH intermediate. In addition, a pathway via the CO intermediate toward CH_3_OH has been included for the Ni_1_-adsorbed and Ni_8_-cluster model surfaces.

We will discuss the elementary reaction steps in this network for the Ni_1_-doped, Ni_1_-adsorbed, and Ni_8_-cluster model surfaces and highlight the main trends in activation energies and transition-state structures. We also compare key elementary reaction steps for the formation of CO, CH_4_, and CH_3_OH between Ni_6_ and Ni_8_ clusters. The activation barriers are given with respect to the most stable adsorbed state for each intermediate. All the elementary reaction steps along with the corresponding forward and backward activation energies are tabulated in the Supporting Information in Section S7 (Tables S9a–c). The geometries corresponding to the initial, transition, and final states are reported in Sections S8–S10 of the Supporting Information.

#### 3.3.1 Methanol Synthesis on Ni_1_-Doped In_2_O_3_

**Figure 4:**
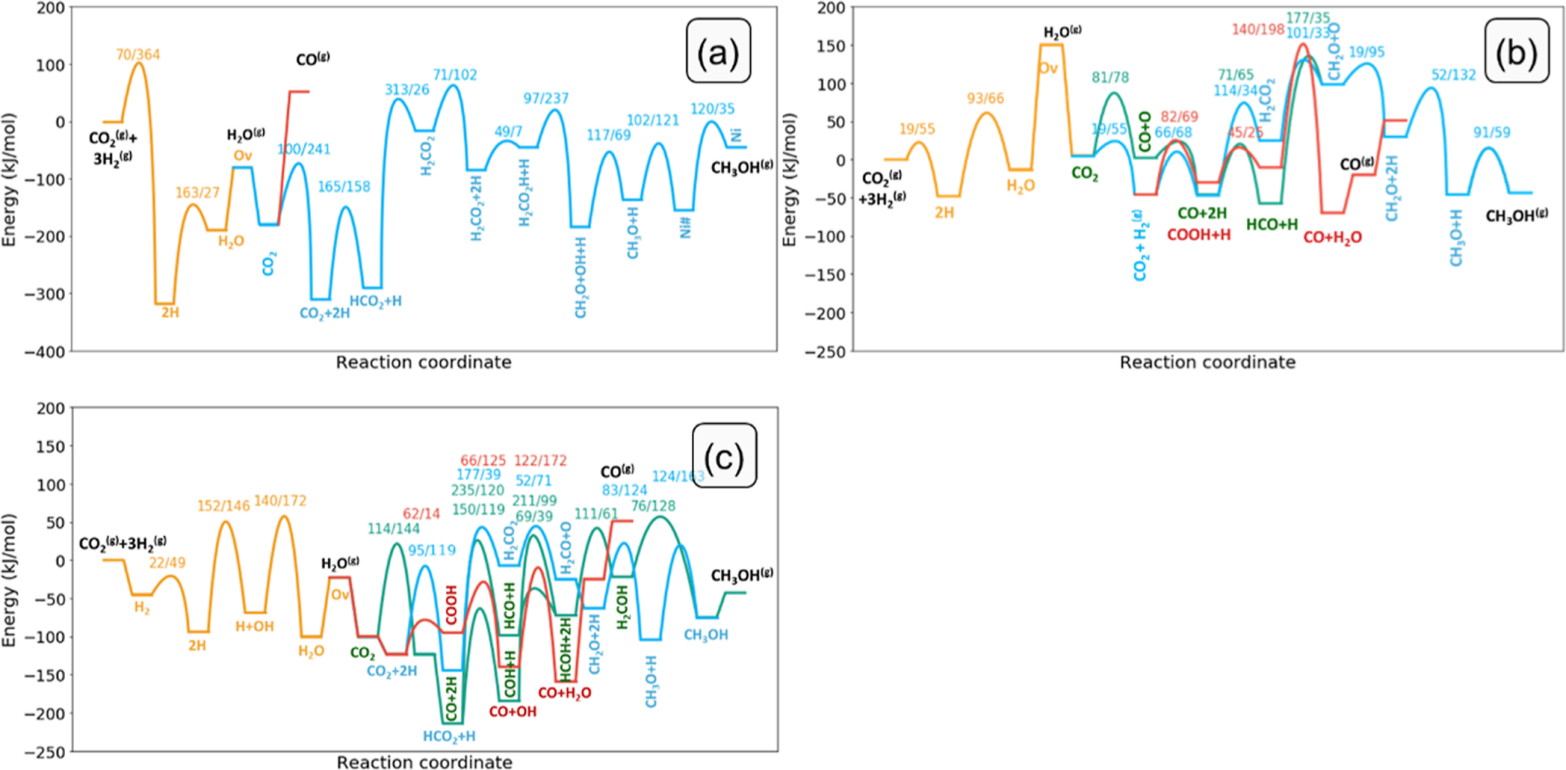
PEDs of the conversion of CO_2_ and H_2_ to CO, CH_3_OH, and H_2_O on (a) Ni_1_-doped, (b) Ni_1_-adsorbed, and (c) Ni_8_-cluster model. Reaction barriers are reported in the X/Y format, wherein X corresponds to the forward barrier and Y to the backward barrier. All energies are given in kJ/mol. Orange: oxygen vacancy formation pathway; blue: formate pathway to CH_3_OH; green: CO hydrogenation pathway to CH_3_OH; red: rWGS (direct or H-assisted) to CO.

We first discuss the reaction energetics of CO_2_ hydrogenation to methanol over the Ni_1_-doped system. The reaction network is shown in Figure 3a and the corresponding potential energy diagram (PED) in Figure 4a. The geometries of initial, transition, and final states can be found in Section S8. H_2_ dissociation on the Ni_1_-doped In_2_O_3_(111) surface (step 1) is homolytic, similar to the In_2_O_3_ surface and leads to the formation of two OH^δ+^ groups.^72^ This step has an activation energy of 70 kJ/mol and is exothermic by Δ*E*_r_ = −294 kJ/mol, indicative of the high stability of the surface hydroxyl groups. The relatively high barrier for H_2_ activation can be understood by considering that the surface O anions cannot stabilize the negatively charged H atoms during dissociation.^43^ Subsequent water formation has a barrier of 163 kJ/mol and is endothermic by 137 kJ/mol. H_2_O desorption (Δ*E*_des_ = 80 kJ/mol; step 3) completes oxygen vacancy formation. Overall, the process of oxygen vacancy formation is associated with a reaction energy of −80 kJ/mol and an activation barrier of 70 kJ/mol with respect to gas-phase H_2_.

Next, CO_2_ adsorbs at the oxygen vacancy (step 4). The carbon atom coordinates to a lattice oxygen instead of the Ni SA, which is due to the steric hindrance around Ni due to its location on the surface. The adsorption energy of CO_2_ is exothermic by Δ*E*_ads_ = −103 kJ/mol. Dissociative adsorption of another H_2_ molecule (step 5) leads to two hydroxyl groups adjacent to CO_2_. This step has an activation energy of 104 kJ/mol and is exothermic by Δ*E*_r_ = −22 kJ/mol. CO_2_ hydrogenation to HCO_2_ + H (step 6) proceeds by proton migration from an adjacent hydroxyl species with a forward activation energy of 165 kJ/mol (Δ*E*_r_ = 8 kJ/mol). Another proton migration step leads to H_2_CO_2_ (step 7) with a forward barrier of 313 kJ/mol (Δ*E*_r_ = 287 kJ/mol). Notably, high activation energies are associated with the formation of a bond between a slightly positively charged C atom and the H atom of the OH group, which is also positively charged, in line with a previous work on In_2_O_3_.^24^ The possibility of hydrogenating one of the O atoms in HCO_2_ giving HCOOH was also investigated. However, no TS could be found for this elementary step because the HCOOH moiety does not adsorb stably on the surface. No TS has also been found for the direct dissociation of H_2_CO_2_ into CH_2_O + O. However, the CH_2_O intermediate can be obtained from H_2_CO_2_H dissociation. After dissociative adsorption of another H_2_ molecule (step 8; *E*_act_ = 71 kJ/mol, Δ*E*_r_ = −32 kJ/mol), one of the two oxygens of H_2_CO_2_ is protonated (step 9) to form H_2_CO_2_H + H (*E*_act_ = 49 kJ/mol, Δ*E*_r_ = 42 kJ/mol). The subsequent cleavage of a C–O bond of H_2_CO_2_H yields CH_2_O and a OH moiety, which occupies the oxygen vacancy (step 10). This step has an activation energy of 97 kJ/mol and is exothermic by 140 kJ/mol. Next, the CH_2_O moiety is hydrogenated to CH_3_O via proton migration from a neighboring OH group (step 11). This step is endothermic by 49 kJ/mol and has a forward activation energy of 117 kJ/mol. Finally, CH_3_O is hydrogenated by the hydroxyl species obtained from H_2_CO_2_H dissociation, forming methanol, which immediately desorbs (step 12). This concerted elementary step has an activation energy of 102 kJ/mol and is exothermic by Δ*E*_r_ = −19 kJ/mol. Finally, oxygen migration (step 13) restores the initial stoichiometric surface. This step has an activation energy of 120 kJ/mol and is endothermic by Δ*E*_r_ = 85 kJ/mol.

CO is observed during experiments on Ni/In_2_O_3_ as a by-product of the rWGS reaction (Figure 4a, red). On the Ni_1_-doped model, CO can be formed via a redox mechanism involving the formation of H_2_O (steps 1–3), adsorption of CO_2_ on an oxygen vacancy (step 4), and subsequent dissociation and desorption of CO (step 14). The energy penalty associated with replenishing one oxygen vacancy by CO_2_, thus restoring the stoichiometric surface, is 234 kJ/mol. Ye et al. observed that the same process on an In_2_O_3_(110) model surface has a barrier of 1.4 eV.^27,28^ The higher barrier for our model is in keeping with the result that doping In_2_O_3_ with Ni results in a more exothermic oxygen vacancy formation energy. A hydrogen-assisted rWGS pathway to CO via the COOH intermediate is generally also followed. We explored this pathway for the Ni_1_-doped model; however, no TS could be found for the dissociation of COOH into CO and OH as there is no active site to accept the OH.

#### 3.3.2 Methanol Synthesis on Ni_1_-Adsorbed In_2_O_3_

Next, we discuss the mechanism of CO_2_ hydrogenation over the Ni_1_-adsorbed system. The reaction network is shown in Figure 3b and the PED in Figure 4b. The geometries of initial, transition, and final states can be found in Section S9. On this surface, H_2_ is heterolytically dissociated to form a NiH^δ−^ and a OH^δ+^ species (step 1), as we reported in another study.^43^ This step is mildly activated (*E*_act_ = 19 kJ/mol) and exothermic by −36 kJ/mol. Notably, H_2_ dissociation has a lower activation energy compared to the formation of two OH groups, as found for the Ni_1_-doped system (*E*_act_^doped^ = 70 kJ/mol). Next, the formation of an oxygen vacancy proceeds by H migration from the Ni–H moiety to form H_2_O (step 2). This step has an activation energy of 93 kJ/mol and a reaction energy of +27 kJ/mol. Water desorption from the surface (step 3) is associated with a desorption energy of 177 kJ/mol. The overall barrier and reaction energy of oxygen vacancy formation with respect to gas-phase H_2_ on this surface amount to 150 kJ/mol, which is considerably higher than the corresponding values for the Ni_1_-doped surface (70 and −80 kJ/mol, respectively).

CO_2_ adsorbs in the oxygen vacancy, coordinating with the Ni SA (step 4). In its most stable adsorption configuration, the carbon atom of CO_2_ binds to the Ni atom (Δ*E*_ads_ = −144 kJ/mol). A second H_2_ molecule can dissociate, forming two OH species (step 5). Homolytic dissociative adsorption of H_2_ is preferred because of the steric hindrance of the Ni atom, which is also involved in Ni–C bonds with adsorbed CO_2_. This step has an activation energy of 19 kJ/mol and is exothermic by Δ*E*_r_ = −36 kJ/mol. Along the formate pathway to methanol (steps 6–11), first a H species migrates from the lattice oxygen to the Ni SA and hydrogenates CO_2_ to form HCO_2_ (step 6). This step has a forward activation energy of 66 kJ/mol and is slightly exothermic (Δ*E*_r_ = −2 kJ/mol). The subsequent hydrogenation to H_2_CO_2_ (step 7) has a forward activation energy of 114 kJ/mol (Δ*E*_r_ = 80 kJ/mol). Notably, the formation of a C–H bond on the Ni_1_-adsorbed model is easier than on the Ni_1_-doped model. This can be ascribed to the hydride character of the H atom adsorbed to Ni. Next, the cleavage of a C–O bond in the H_2_CO_2_ intermediate takes place (step 8, *E*_act_ = 101 kJ/mol, Δ*E*_r_ = 33 kJ/mol), leading to CH_2_O and an O atom, the latter healing the oxygen vacancy. After adsorption of another H_2_ molecule (step 9; *E*_act_ = 19 kJ/mol; *E*_R_ = −36 kJ/mol), the CH_2_O moiety is hydrogenated to CH_3_O (step 10; *E*_act_ = 55 kJ/mol *E*_R_ = −79 kJ/mol). Finally, CH_3_O hydrogenation to methanol and its subsequent desorption (step 11) take place in a single elementary reaction step with an activation energy of 61 kJ/mol.

On the Ni_1_-adsorbed model surface, methanol can also be obtained via CO hydrogenation, as seen in Figure 3b. This involves steps 12–15 in the CO hydrogenation pathway, followed by steps 9–11 in the formate pathway. Upon adsorption on an oxygen vacancy, CO_2_ can directly dissociate (step 12) into CO and O (*E*_act_ = 81 kJ/mol, *E*_R_ = 3 kJ/mol), where the O replenishes the oxygen vacancy, restoring the stoichiometric surface. From this state, CO is further hydrogenated to HCO (step 14) after adsorption of another H_2_ molecule (step 13; *E*_act_ = 19 kJ/mol; *E*_R_ = −36 kJ/mol). Herein, CO hydrogenation to HCO has an activation energy of 71 kJ/mol (*E*_R_ = 6 kJ/mol). Subsequently, HCO is hydrogenated to CH_2_O (step 15), which has a barrier of 177 kJ/mol and is endothermic by 142 kJ/mol. The resulting CH_2_O fragment is part of the formate pathway and, thus, its subsequent hydrogenation of methanol can proceed via this pathway.

The CO by-product can be obtained via formation of H_2_O (steps 1–3), followed by direct cleavage of the C–O bond in CO_2_ and subsequent desorption of CO from a stoichiometric surface (steps 12 and 20). The desorption of CO from a stoichiometric surface is associated with a barrier of 121 kJ/mol. In addition, a H-assisted rWGS pathway was investigated. After adsorption of CO_2_ on an oxygen vacancy (step 5) and dissociative adsorption of H_2_ (step 6), protonation of CO_2_ takes place (step 16), yielding COOH (*E*_act_ = 82 kJ/mol; *E*_R_ = 13 kJ/mol). Next, COOH dissociates (step 17) into CO and OH (*E*_act_ = 45 kJ/mol, *E*_R_ = 20 kJ/mol). OH hydrogenation to form water (step 18) is associated with an activation energy of 140 kJ/mol and is endothermic by −58 kJ/mol. Finally, desorption of CO and H_2_O (steps 19 and 3) proceeds with desorption energies of 51 and 177 kJ/mol, respectively, closing the catalytic cycle.

#### 3.3.3 Methanol Synthesis on Ni_8_-Cluster In_2_O_3_

The network diagram and PEDs of CO_2_ conversion to CH_3_OH and CO on the Ni_8_-cluster model are depicted in Figures 3c and 4c, respectively. The geometries of initial, transition, and final states can be found in Section S10. H_2_ adsorbs molecularly on the nanocluster (Δ*E*_r_ = −49 kJ/mol) and then homolytically dissociates into H atoms (*E*_act_ = 22 kJ/mol, Δ*E*_r_ = −40 kJ/mol). Overall, the process of H_2_ activation (step 1) is exothermic by −89 kJ/mol. Notably, H_2_ activation on the Ni_8_-cluster model is more facile than on the SA models, which is expected for a Ni cluster with a metallic character. Next, one interfacial O atom can be hydrogenated to form H_2_O. The barriers for the two consecutive hydrogenation steps are 152 kJ/mol (step 2) and 140 kJ/mol (step 3). Water desorption from the surface (step 4) costs 80 kJ/mol. The oxygen vacancy formation involves overall reaction and activation energies of −22 and 57 kJ/mol, respectively, with respect to gas-phase H_2_. On the oxygen-defective surface, CO_2_ adsorbs (Δ*E*_ads_ = −120 kJ/mol; step 5) at the metal–oxide interface with the carbon coordinating to a lattice oxygen next to two Ni–H fragments. From this state, CO_2_ can be hydrogenated to methanol via a formate intermediate (steps 7–12). First, CO_2_ is hydrogenated to HCO_2_ by a Ni–H species (step 7), which is exothermic by −25 kJ/mol and has an activation energy of 95 kJ/mol. Next, HCO_2_ is hydrogenated to H_2_CO_2_ (step 8) by another Ni–H species. This step features the highest forward barrier of the formate pathway (*E*_act_ = 177 kJ/mol) and is endothermic by Δ*E*_r_ = 138 kJ/mol. Next, cleavage of a C–O bond in the H_2_CO_2_ intermediate yields CH_2_O and O (step 9), the latter replenishing the oxygen vacancy. This elementary reaction step has a barrier of 52 kJ/mol and is exothermic by Δ*E*_r_ = −30 kJ/mol. After adsorption of another H_2_ on the Ni_8_ cluster (step 10; Δ*E*_r_ = −89 kJ/mol), two further hydrogenation steps toward CH_3_O and CH_3_OH with activation energies of 83 kJ/mol (step 11; Δ*E*_r_ = 41 kJ/mol) and 124 kJ/mol (step 12; Δ*E*_r_ = −39 kJ/mol), respectively, lead to methanol. Desorption of methanol costs 35 kJ/mol (step 13).

Besides H-assisted CO_2_ dissociation via the formate intermediate, C–O bond scission can also proceed in a direct fashion following oxygen vacancy formation. Herein, the vacancy is healed upon C–O bond scission in adsorbed CO_2_. CO_2_ can adsorb on the Ni_8_ cluster with a slightly lower adsorption energy of Δ*E*_ads_ = −88 kJ/mol than on the vacancy (−120 kJ/mol). Subsequent dissociation of CO_2_ adsorbed on the cluster results in an O atom that heals the oxygen vacancy and adsorbed CO. This reaction has a barrier of 114 kJ/mol and is exothermic by 30 kJ/mol (step 14). This barrier is higher than the corresponding barrier over the Ni_1_-adsorbed system (*E*_act, adsorbed_ = 81 kJ/mol). CO is a precursor for methanol synthesis along the CO-hydrogenation pathway (Figures 3c and 4c, green). After CO_2_ dissociation, CO can either desorb (Δ*E*_ads_ = −130 kJ/mol; step 28), which would be representative of an alternative rWGS pathway to the one discussed below or be hydrogenated to either HCO (*E*_act_ = 150 kJ/mol; step 16) or COH (*E*_act_ = 235 kJ/mol; step 17). HCO can be hydrogenated toward HCOH (step 18). This step is endothermic by 30 kJ/mol and features a barrier of 69 kJ/mol. Alternatively, HCOH can also be formed from COH by hydrogenation involving a barrier of 211 kJ/mol (step 20; Δ*E*_r_ = 50 kJ/mol). HCOH can subsequently be hydrogenated to H_2_COH (step 22; Δ*E*_r_ = −52 kJ/mol) and then to CH_3_OH (step 23). These two hydrogenation steps have activation energies of *E*_act_ = 111 kJ/mol and *E*_act_ = 76 kJ/mol, respectively.

To verify our hypothesis that CO_2_ methanation on small In_2_O_3_-supported Ni clusters does not occur, we computed the barriers for direct and H-assisted CO dissociation. These steps are most likely the rate-limiting steps in CO_2_ methanation.^73^ The barriers for these steps are reported in Table S10, and the structures of the TS can be found in Table S11. We can compare these results to data for CO dissociation pathways on extended surfaces of Ni by Sterk et al.^73^ Compared to Ni(110), direct cleavage of the C–O bond on a Ni_8_-cluster model is associated with a very high barrier (*E*_act_ = 312 kJ/mol for the cluster vs *E*_act_ = 150 kJ/mol for Ni(110)). On the Ni_8_ cluster, the barrier of H-assisted CO dissociation via an HCO intermediate of 174 kJ/mol is more facile than the barrier for direct CO dissociation. However, this barrier is still significantly higher than the one computed for Ni(110) (*E*_act_ = 117 kJ/mol). It is likely that the structure of the Ni_8_-cluster model would also lead to high barriers for alternative CO dissociation reactions involving COH, H_2_CO, and H_3_CO intermediates due to the absence of step-edge sites, in line with the expectations based on experimental evidence.^43^

In line with the Ni_1_-adsorbed model, on the Ni_8_-cluster model, CO can be obtained via formation of H_2_O (steps 1–4), followed by direct cleavage of the C–O bond in CO_2_ and subsequent desorption of CO from a stoichiometric surface (steps 14 and 28, respectively). The desorption of CO from a stoichiometric surface has a barrier of 130 kJ/mol. Finally, we discuss the formation of CO from CO_2_ via the H-assisted rWGS pathway. Herein, one of the oxygen atoms of the CO_2_ molecule is protonated to form COOH (step 24) with an activation energy of 62 kJ/mol. This step is endothermic by 48 kJ/mol. In turn, COOH can dissociate on the Ni_8_ cluster into CO and OH (step 25; *E*_act_ = 66 kJ/mol). This step is exothermic by 59 kJ/mol. The resulting hydroxyl fragment is hydrogenated to H_2_O (step 26; *E*_act_ = 122 kJ/mol). Finally, H_2_O and CO desorb, leaving a vacancy on the surface with Δ*E*_des_ of 80 and 114 kJ/mol, respectively.

### 3.4 Microkinetic Simulations

#### 3.4.1 Overall Kinetics

To compare the catalytic performance of different Ni/In_2_O_3_ models, we compute the CO_2_ hydrogenation reaction rate employing microkinetic simulations and the DFT reaction energetics. The active sites in our model consist of either isolated single Ni atoms, that is, either the Ni_1_-adsorbed or the Ni_1_-doped model or clusters (Ni_8_- and Ni_6_-cluster models) stabilized on the In_2_O_3_ support. We do not take migration of intermediates between the active sites into account. Co-adsorbed species are modeled as distinct varieties of a single active site. In this approximation, all elementary reaction steps are unimolecular, with the exception of the adsorption and desorption steps. A detailed list of the elementary reaction steps is provided in the Supporting Information.

**Figure 5:**
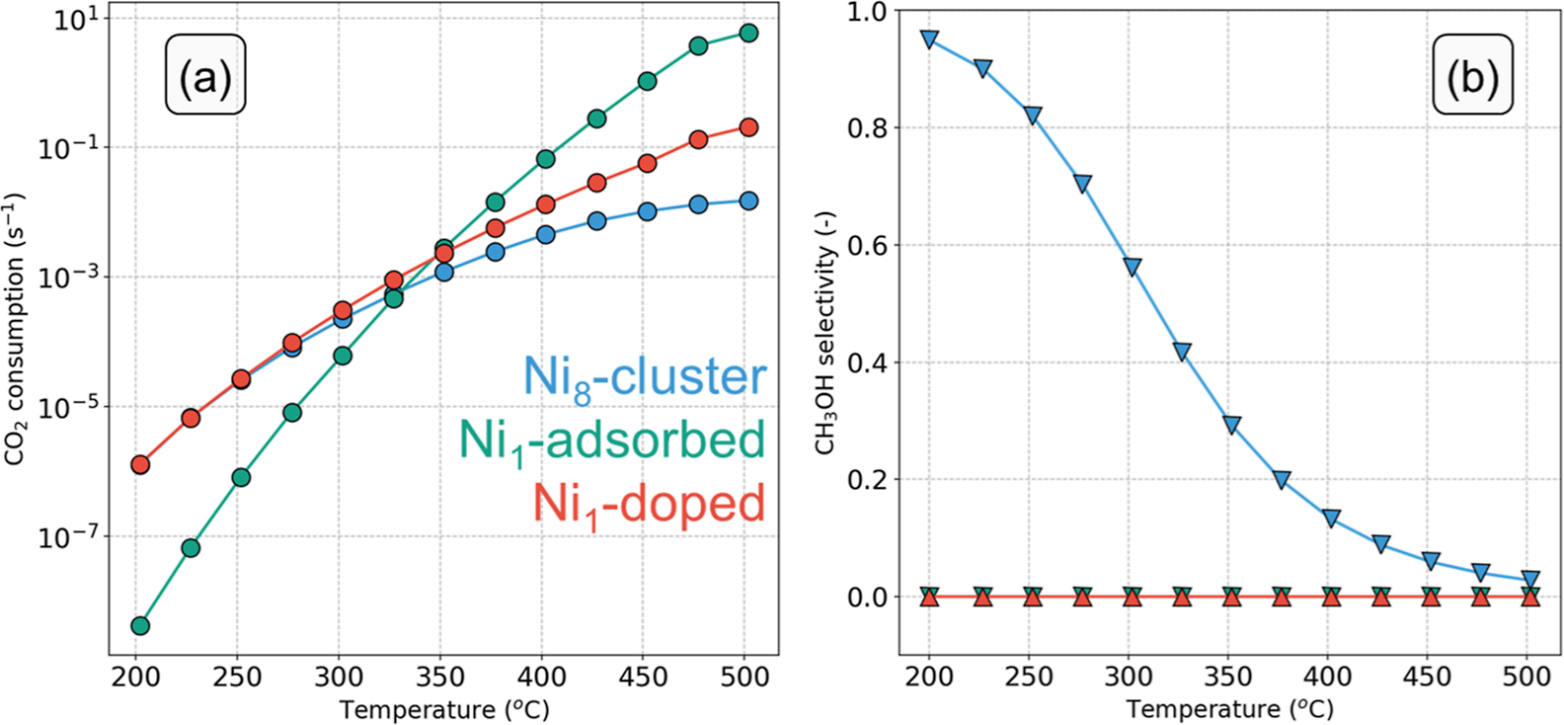
(a) CO_2_ consumption rate (s^–1^) and (b) CH_3_OH selectivity as a function of temperature on different models (*p* = 50 bar, H_2_/CO_2_ ratio = 5).

The CO_2_ consumption rate and the CH_3_OH selectivity as a function of temperature are plotted in Figure 5 for the three systems considered. The turnover frequencies (TOF) toward CH_3_OH and CO are given in Figure S9. As can be seen from Figure 5a, the Ni_1_-doped and Ni_8_-cluster models exhibit the highest CO_2_ consumption rate below 275 °C. Above 300 °C, the Ni_1_-adsorbed and Ni_1_-doped systems are more active than the Ni_8_-cluster model. From Figure 5b, it can be seen that the CH_3_OH selectivity for the Ni_8_ cluster is 95% at 200 °C. With increasing temperature, the rate of CO formation increases faster than the rate of methanol formation (Figure S9a,b), resulting in a decrease in methanol selectivity. Above 350 °C, CO becomes the main reaction product, in line with experimental results. Both the Ni_1_-adsorbed and Ni_1_-doped models show negligible CH_3_OH selectivity (Figure 5b), indicating that these two models are mainly active for the rWGS reaction.

To highlight the promoting effect of Ni on In_2_O_3_, we constructed a microkinetic model for unpromoted In_2_O_3_ based on the published DFT data of Frei et al.^24^ The resulting predictions in terms of activity and selectivity are provided in Figure S10. These data clearly show the promoting effect of Ni clusters on In_2_O_3_ for CO_2_ hydrogenation at relevant temperatures. Notably, these results are qualitatively in line with the experimental results published by Jia et al.^39^ Overall, this implies that the Ni_8_–In_2_O_3_ model presents the highest methanol reaction rate in line with our earlier experimental work.^69^

We also verified that methane formation on the Ni_8_-cluster model is unlikely by performing microkinetic simulations using the barrier for direct CO dissociation calculated on the Ni_8_ cluster (312 kJ/mol) and using the data by Sterk et al.^73^ for the C–H coupling steps. Sensitivity analysis shows that appreciable methane selectivity takes place only if the activation energy of the CO dissociation step is below 60 kJ/mol (Figure S11).

**Figure 6:**
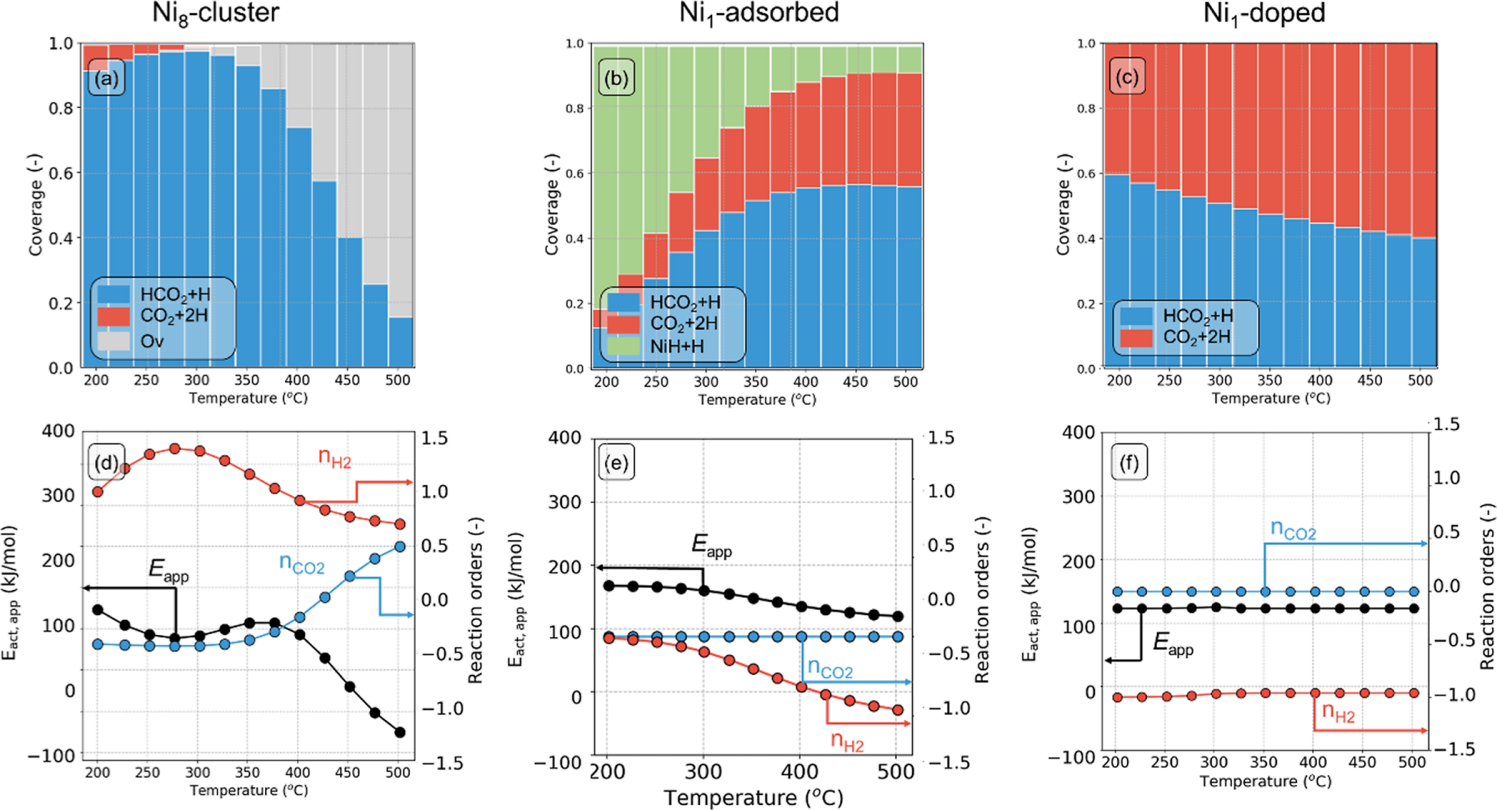
Surface state of the model catalysts (a–c), reaction orders and apparent activation energies (d−f) with CO_2_ as the key component as a function of temperature.

The results in terms of coverage, apparent activation energy (*E*_app_), and reaction orders as a function of temperature are given in Figure 6. The coverages in Figure 6a–c should be interpreted as the fraction of time the system spends in a particular state (i.e., the time average). According to the ergodicity principle in statistical thermodynamics, this equals the fraction of active sites that are in a particular state (i.e., the ensemble average). The Ni_8_ cluster (Figure 6a) predominantly resides in the HCO_2_ + H state at lower temperatures (200 °C < *T* < 300 °C). The reaction order in H_2_ (Figure 6d) is positive, while the reaction order in CO_2_ is close to zero. A higher H_2_ partial pressure is beneficial because it increases the rate of hydrogenation of HCO_2_ to methanol. As the surface is dominated by a CO_2_-derived intermediate, further increasing the CO_2_ partial pressure will not increase its coverage and the reaction rate, explaining the close to zero reaction order in this reactant. The kinetic parameters for the Ni_8_-cluster model lie within the range of values reported in the experimental literature for In_2_O_3_. For instance, Frei et al. reported a positive reaction order in H_2_ (0.33) and a close-to-zero order for CO_2_ at 250 °C.^72^ We find that, with increasing temperature, the reaction order in H_2_ decreases and the one in CO_2_ increases. This is because the oxygen vacancy state becomes the dominant state at high temperature (Figure 6a, in gray). In the limit of the empty surface, a higher partial pressure of CO_2_ results in a higher coverage with reaction intermediates, increasing the CO_2_ turnover rate. This behavior is also reflected in the *E*_app_ trend (Figure 6d). The *E*_app_ is approximately constant below 400 °C and decreases at higher temperature. This reflects a change in the changing coverages. By comparing Figure 6a,d, it can be seen that the decrease in the apparent activation energy goes together with an increase in the oxygen vacancies, resulting in a higher coverage of CO_2_. The exothermic energy of CO_2_ adsorption (Δ*E*_ads_ = −120 kJ/mol) lowers the apparent activation energy of the overall reaction. The computed *E*_app_ for the Ni_8_-cluster model is in line with the experimental values reported on Ni/In_2_O_3_ (80 kJ/mol between 200 and 300 °C).^39^ We also performed simulations with either CO or CH_3_OH as the key component to better understand the decrease of the *E*_app_ with decreasing temperature (Figure S12). On the Ni_8_-cluster, the *E*_app_ for CH_3_OH synthesis is lower than the one for CO production (Figure S12a), in line with the selectivity trend depicted in Figure 5b. The trends in kinetic parameters as shown in Figure 6a,d for the Ni_8_ cluster are also in keeping with the changing selectivity from CH_3_OH to CO. To further understand these aspects, we investigate these trends in more detail with a DRC and a flux analysis (vide infra).

For the Ni_1_-adsorbed model (Figure 6b), at lower temperature, the dominant surface state features two adsorbed H species (NiH + H) on a stoichiometric Ni_1_–In_2_O_3_(111) surface. Subsequent water desorption along the oxygen vacancy formation pathway is associated with a relatively high barrier (Δ*E*_des, H_2_O_ = 177 kJ/mol). This step is the most difficult one along the most favorable pathway, as can be seen in Figures 3b and 4b. As a result, the NiH + H state is dominant. With increasing temperatures, H_2_O desorption will be easier, resulting in more vacancies, where CO_2_ can adsorb. This results in a decreasing contribution of the NiH + H state in favor of intermediates in the CO_2_ hydrogenation pathway, such as CO_2_ + 2H and HCO_2_ + H. The reasons behind the latter two states being dominant can be understood from Figure 3b. CO_2_ binds strongly to the surface (Δ*E*_ads_ = −144 kJ/mol), and subsequent hydrogenation of CO_2_ to HCO_2_ has a low activation energy of only 66 kJ/mol (step 6 in Figure 3b), whereas the subsequent hydrogenation step to H_2_CO_2_ has a considerably higher activation energy of 114 kJ/mol (step 7 in Figure 3b). Consequently, the HCO_2_ + H state together with the CO_2_ + 2H state, which only differ by 2 kJ/mol, are found to be the dominant states.

At low temperatures, the reaction orders in both CO_2_ and H_2_ (Figure 6e) are close to zero. Under these conditions, the reaction is limited by the rate of H_2_O removal, which is the last step toward oxygen vacancy formation (step 3 in Figure 3b). The rate of H_2_O desorption is not affected by the partial pressure of H_2_ and CO_2_. A temperature increase thus results in the formation of oxygen vacancy and CO_2_ adsorption (Figure 6b). Under these conditions, an increased partial pressure of H_2_ leads to a higher rate of H adsorption which, together with the presence of adsorbed CO_2_, results in CO_2_ + 2H and HCO_2_ + H states becoming dominant. However, these elementary reaction steps proceed toward a branch in the kinetic network away from the dominant product (CO). Thus, under these conditions, the reaction order in H_2_ is negative.

For the Ni_1_-adsorbed model, *E*_app_ (Figure 6e) is constant at 175 kJ/mol for temperatures below 300 °C. This value corresponds to the desorption energy of H_2_O. As the temperature increases, the *E*_app_ decreases. On this model, the *E*_app_ for the CH_3_OH product is higher than the *E*_app_ for the CO by-product (Figure S12b), in line with the selectivity trend depicted in Figure 5b. Together, these findings suggest that oxygen vacancy formation controls the overall reaction rate at low temperatures while, at high temperatures, either CO_2_ activation or hydrogenation are limiting the overall rate. These aspects can be better appreciated by means of a DRC analysis (vide infra).

When Ni is doped inside In_2_O_3_ (Figure 6c), the surface is predominantly in the CO_2_ + 2H and HCO_2_ + H working states. Although CO_2_ hydrogenation to HCO_2_ is possible on the catalyst’s surface, further hydrogenation toward methanol is limited by the high barrier for HCO_2_ hydrogenation to H_2_CO_2_ (*E*_act_ = 313 kJ/mol, Δ*E*_r_ = 287 kJ/mol). The reaction order in CO_2_ is zero because the dominant working state (either CO_2_ + 2H or HCO_2_ + H) already corresponds to intermediates derived from CO_2_. The reaction order in H_2_ is negative at low temperature, indicating that a higher partial pressure of H_2_ would push the network away from the dominant pathway. On the Ni_1_-doped model, the *E*_app_ (Figure 6f) is constant to a value of 130 kJ/mol in the explored temperature range. In line with the Ni_1_-adsorbed model, on the Ni1-dopped system, the *E*_app_ for the CH_3_OH product is higher than the *E*_app_ for the CO by-product (Figure S12c).

#### 3.4.2 Rate and Selectivity Control of the Reaction Network

In this section, we discuss in detail the reaction network that leads to CH_3_OH and CO formation from CO_2_ hydrogenation on the three Ni/In_2_O_3_ models. We identify the elementary steps that control the overall CO_2_ consumption rate and the CH_3_OH selectivity and investigate how these steps change with reaction temperature. For this purpose, we conduct a sensitivity analysis based on Campbell’s DRC^63^ and DSC.^65,74^ Under zero extent of reaction, the sum of the DRC coefficients is conserved at one.^75^ The DSC quantifies the extent to which a particular elementary reaction step influences the selectivity of certain products for which methanol is of our primary interest. Note that the sum of the DSC values of all elementary reaction steps for a single product equals zero.^64^

##### 3.4.2.1 Ni_8_-cluster

**Figure 7:**
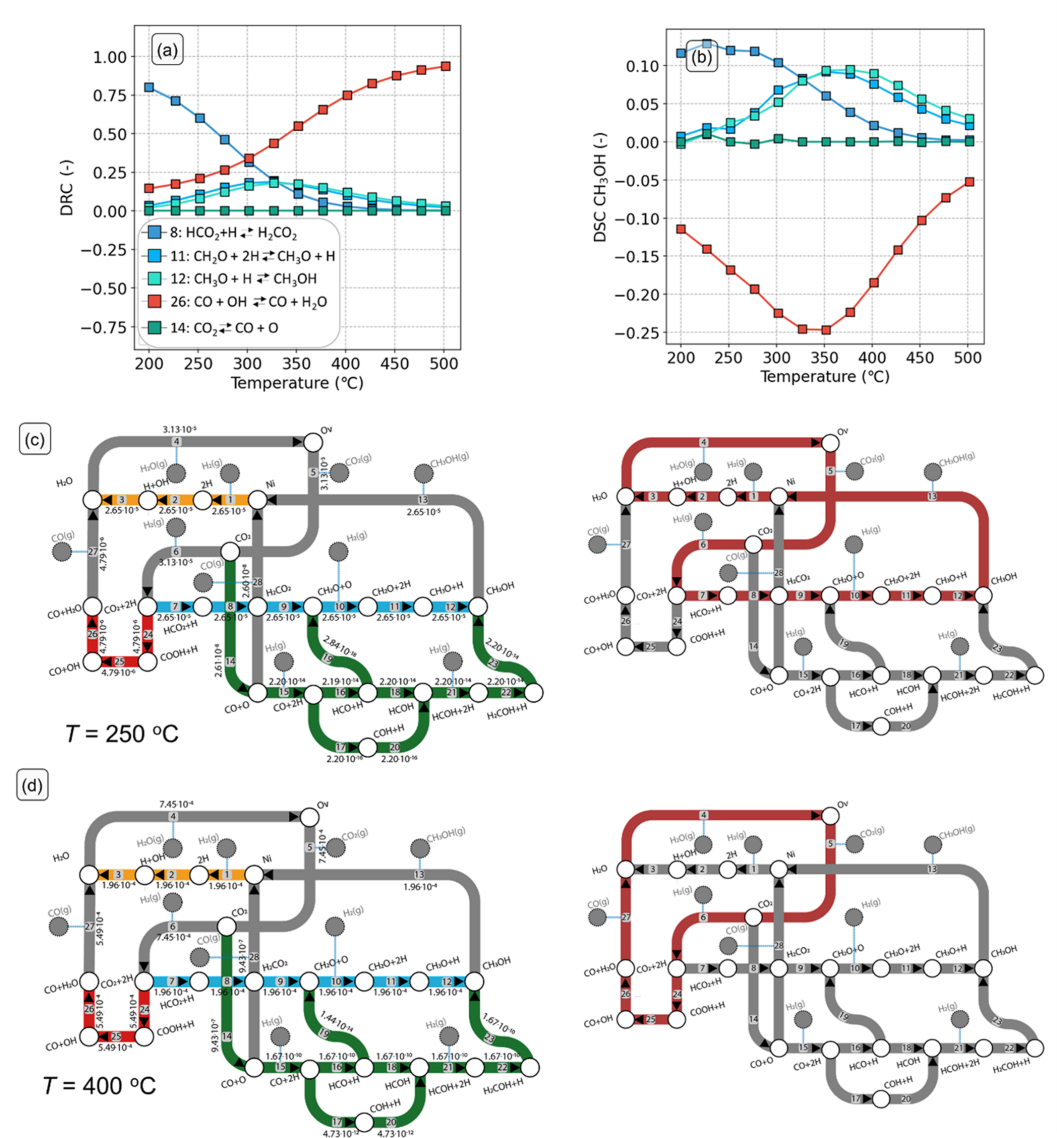
DRC (a) and DSC (b) with CO_2_ as the key component as a function of temperature for the Ni_8_-cluster model. (c,d) Reaction network analysis (left) and dominant pathways (right) for CO_2_ hydrogenation to CH_3_OH, CO, and H_2_O. (c) *T* = 250 °C and (d) *T* = 400 °C (*p*_tot_ = 50 bar, H_2_/CO_2_ = 5). The numbers in the arrows are molar reaction rates (s^–1^) and are normalized with respect to the amount of adsorbed CO_2_. The pathways with the highest molar fluxes are highlighted in red in the right part of the figure.

The DRC and DSC analyses for the Ni_8_-cluster model are reported in Figure 7a,b, respectively. From these figures, it can be seen that at low temperatures (200 °C < *T* < 300 °C), the rate of CO_2_ consumption is almost exclusively controlled by the rate of HCO_2_ hydrogenation to H_2_CO_2_ (step 8 in Figure 7c), which has the highest activation energy in the dominant pathway as seen from Figure 7c (pathway highlighted in red). Other elementary steps, such as CH_2_O and CH_3_O hydrogenation (steps 11 and 12 in Figure 7c), control the kinetics to a smaller extent. These results are consistent with the positive reaction order in H_2_ and a reaction order in CO_2_ of zero (Figure 6d). A higher partial pressure of H_2_ would increase the concentration of Ni–H states necessary for the hydrogenation reactions, thus enhancing the overall rate, whereas a change in the partial pressure of CO_2_ would not affect these reactions. The DSC analysis (Figure 7b) shows that the same elementary steps that are controlling the rate are also controlling methanol selectivity. An increased rate of HCO_2_ hydrogenation (step 8) would result in a higher flux in the route toward methanol, benefiting its formation at the expense of CO formation. With increasing temperatures, a change in the selectivity from methanol to CO is observed (Figure 5b), which is reflected by the DRC analysis. Herein, the DRC coefficient of HCO_2_ hydrogenation to H_2_CO_2_ decreases as a function of temperature, whereas the DRC coefficient of OH hydrogenation to H_2_O (step 26 in Figure 7c) increases. This last step pertains to the rWGS branch in the kinetic network. Thus, this step is observed to inhibit the selectivity toward methanol. Figure 7b shows that, at higher temperatures, the steps of CH_2_O hydrogenation to CH_3_O and its further hydrogenation to CH_3_OH (steps 11 and 12, respectively) control the selectivity to the desired methanol product. These two steps require more Ni–H species to occur than HCO_2_ hydrogenation (step 8). However, Ni–H species are less abundant at high temperature, as we previously observed (Figure 6a), suggesting that steps requiring less surface hydrogen, like the ones pertaining the rWGS pathway, are facilitated at high temperature. This can explain the observed shift in selectivity toward CO and is also consistent with the decrease in the computed reaction orders in H_2_ (Figure 6d). To confirm the effect of H_2_ partial pressure on CH_3_OH selectivity, we performed a simulation with a H_2_/CO_2_ ratio of 1:5 (instead of 5:1) and observed that the selectivity to CH_3_OH decreases to 20% at 250 °C (Figure S13). Thus, the selectivity shift to CO observed at high temperatures can be explained by the lower availability of Ni–H species at such temperatures.

Figure 7c,d shows the flux analysis at low and high temperatures, respectively. Following the pathways with the largest molar fluxes readily reveals the dominant kinetic pathway at those conditions (highlighted in red). At low temperatures (Figure 7c), the dominant pathway corresponds to methanol formation via formate, whereas at high temperatures (Figure 7d), the dominant pathway shifts to the H-assisted rWGS route. This is consistent with the activity and selectivity trends predicted by the microkinetic model. The reaction flux diagram also shows that the pathway of CO hydrogenation to methanol features significantly lower rates (2.20 × 10^–14^ mol s^–1^; steps 14–23) than the formate pathway (2.65 × 10^–5^ mol s^–1^; steps 7–12), indicating that it is not taken. Indeed, hydrogenation of CO to HCO and CO to COH is associated with activation energies of 150 and 235 kJ/mol, respectively, while CO desorption is associated with a lower barrier of Δ*E*_des_ = 130 kJ/mol. Therefore, the desorption of CO is more favorable than its further hydrogenation to methanol. Concerning the formation of CO, Figure 7c,d shows that direct CO_2_ dissociation has lower rates (2.61 × 10^–8^ mol s^–1^; step 14) as compared to the H-assisted rWGS pathway (4.79 × 10^–6^ mol s^–1^; step 24). This difference can be ascribed to the higher activation energy for direct CO_2_ dissociation as compared to its hydrogenation to COOH (114 and 62 kJ/mol, respectively), which makes the H-assisted rWGS more facile.

##### 3.4.2.2 Ni_1_-doped and Ni_1_-adsorbed

From Figure 5b, it was established that neither the Ni_1_-doped nor the Ni_1_-adsorbed models produce methanol. As such, we only consider the DRC and flux analyses as the DSC analysis will evidently only show coefficients close to zero for all elementary reaction steps.

**Figure 8:**
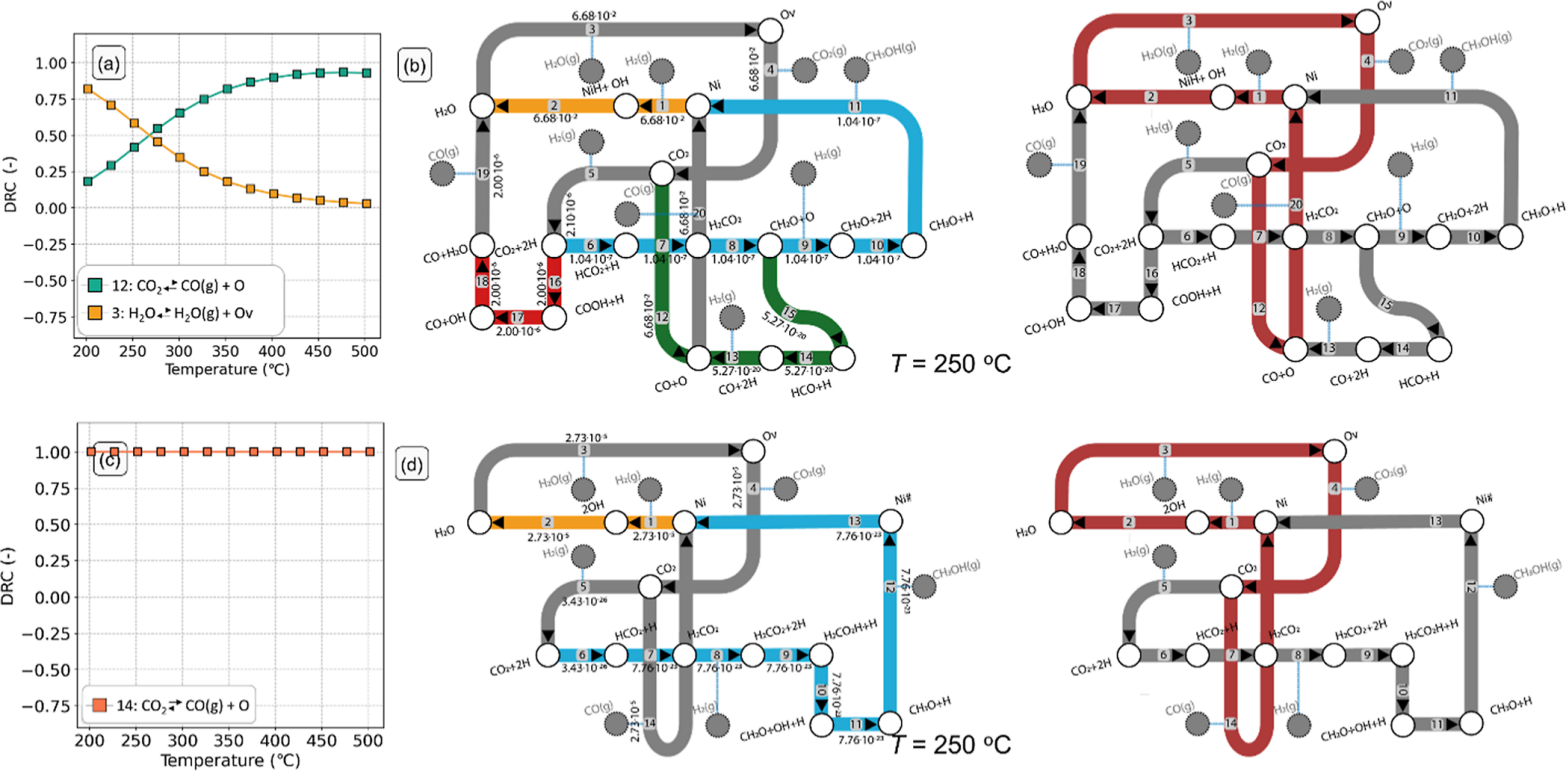
DRC analysis with CO_2_ as the key component as a function of temperature for (a) Ni_1_-adsorbed and (c) Ni_1_-doped model surfaces. Reaction network analysis (left) and dominant pathways (right, highlighted in red) for CO_2_ hydrogenation to CH_3_OH, CO, and H_2_O for (b) Ni_1_-adsorbed and (d) Ni_1_-doped model surfaces. (*T* = 250 °C, *p*_tot_ = 50 bar, H_2_/CO_2_ = 5). The numbers in the arrows are molar reaction rates (s^–1^) and are normalized with respect to the amount of adsorbed CO_2_. The pathways with the highest molar fluxes are highlighted in red.

The DRC analysis and reaction flux analysis for the Ni_1_-adsorbed model are shown in Figure 8a,b. At low temperatures, the CO_2_ consumption rate is mostly controlled by the rate of H_2_O desorption (step 3) because this elementary step has the highest barrier among the steps pertaining to the oxygen vacancy formation pathway (Δ*E*_des_ = 177 kJ/mol). This result is consistent with the surface state of the catalyst featuring mostly adsorbed hydrogen species (NiH + H, in Figure 6b), which is a state that precedes H_2_O formation. At higher temperatures (*T* > 275 °C), the formation of oxygen vacancies and subsequent adsorption of CO_2_ result in a decrease in the DRC coefficient of H_2_O desorption in favor of CO_2_ dissociation (step 12), which becomes the dominant rate-controlling step. An increased rate of CO_2_ dissociation (step 12) would result in a higher rate toward the main product CO. As can be seen in Figure 8b, the branch of the mechanism going toward CO_2_ hydrogenation via the formate pathway features considerably lower rates than the one proceeding toward CO_2_ dissociation (2.10 × 10^–6^ and 6.68 × 10^–2^ s^–1^, respectively). The formate pathway is not taken because the steps of H_2_CO hydrogenation to H_2_CO_2_ (step 7) and its further dissociation into CH_2_O and O (step 9) are highly activated and endothermic, resulting in an overall reaction energy and barrier of 150 and 172 kJ/mol, respectively. The dominant pathway (highlighted in red in Figure 8b, right) on the Ni_1_-adsorbed model proceeds thus via direct dissociation of CO_2_, yielding CO, which desorbs from the surface, closing the rWGS catalytic cycle. In line with the Ni_8_-cluster model, the pathway of CO hydrogenation to methanol is not taken (steps 13–15 in Figure 8b). This is due to the step of HCO hydrogenation to CH_2_O being associated with a high activation energy (*E*_act,f_ = 177 kJ/mol) and being endothermic (Δ*E*_r_ = 142 kJ/mol). Concerning the formation of CO, the direct dissociation of CO_2_ is preferred over the H-assisted rWGS (6.54 × 10^–2^ and 1.05 × 10^–4^ s^–1^, respectively). This is because the H-assisted rWGS pathway is limited by the activation energy of OH hydrogenation (*E*_act_ = 140 kJ/mol), which is significantly higher than that of the CO_2_ dissociation and subsequent CO desorption elementary steps (81 and 121 kJ/mol, respectively).

The DRC analysis and reaction flux analysis for the Ni_1_-doped model are shown in Figure 8c,d, respectively. The elementary reaction step of CO_2_ dissociation and CO desorption (step 14) has a DRC coefficient of 1; thus, it is the rate-determining step (RDS) in the mechanism under the explored temperature range (Figure 8c). This step is rate determining because it has the highest activation energy in the rWGS pathway (Figures 3a and 4a). The dominant pathway for the Ni_1_-doped model at *T* = 250 °C (Figure 8d right highlighted in red) involves the hydrogenation of CO_2_ to CO via a mechanism involving the formation of an oxygen vacancy (steps 1–3), adsorption of CO_2_ on an oxygen vacancy (step 4), subsequent formation of CO, and healing of the vacancy (step 14). Concerning the formation of CH_3_OH, negligible reaction fluxes for the formate pathway are found (3.43 × 10^–26^ s^–1^), in line with the TOF_CH3OH_ computed by the microkinetic model (Figure S7a). This is due to the high activation energy associated with the hydrogenation of HCO_2_ to H_2_CO_2_ (*E*_act_ = 313 kJ/mol, Δ*E*_r_ = 287 kJ/mol), which makes methanol formation unfavorable.

### 3.5 General Discussion

The present study provides new insights into the role of promoting Ni species in the In_2_O_3_-catalyzed hydrogenation of CO_2_ to methanol. Methanol formation involves oxygen vacancies in the In_2_O_3_ support. Frei et al. reported oxygen vacancy formation on a In_2_O_3_(111) surface via homolytic dissociation of molecular hydrogen forming two surface OH groups, followed by water formation and desorption.^72^ Oxygen vacancy formation has an overall barrier of 67 kJ/mol with respect to gas-phase H_2_.^48^ Heterolytic H_2_ dissociation on this surface was found to have a similar overall barrier.^76^ The presence of a Ni_8_-cluster on the In_2_O_3_ surface results in a lower overall barrier (57 kJ/mol), whereas when a single Ni atom is either doped in or adsorbed onto the In_2_O_3_(111) surface, higher overall barriers are found (70 and 150 kJ/mol, respectively). Since the Ni_6_ cluster has the same metallic character and interface with In_2_O_3_ as the Ni_8_-cluster, we infer that the overall barriers to oxygen vacancy formation on the two clusters should be similar. Thus, oxygen vacancies are likely to be present under reaction conditions of methanol synthesis. Their formation and role in the reaction mechanism were explicitly taken into account in the microkinetic models for methanol synthesis based on DFT calculations covering reaction mechanisms involving methanol formation via formates and CO.

On the Ni_8_-cluster model, at relatively low temperatures, the rate of CO_2_ consumption is mainly limited by the elementary step of HCO_2_ hydrogenation to H_2_CO_2_. The main reaction pathway to methanol on the Ni_8_ cluster is via low-barrier C–O bond dissociation in a H_2_CO_2_ intermediate. This results in a high methanol selectivity at low temperatures. With increasing temperatures, the selectivity shifts to CO in line with experimental observations.^39,43^ The dependence of the selectivity on the temperature is associated with the availability of NiH^δ−^ species. The analysis of the surface coverages shows that, at relatively low temperature, hydrides mainly occupy the surface, favoring hydrogenation reactions. With increasing temperatures, the coverage of surface hydrogen rapidly decreases, as it is used to reduce the surface by creating oxygen vacancies. Since the rWGS reaction requires only two hydrogenation steps, whereas methanol formation requires four, the former reaction becomes dominant under surface-hydrogen-lean conditions.

The outcomes of the simulations might be affected by the size and shape of the cluster. For this reason, we compare our Ni_8_ cluster with a smaller Ni_6_-cluster, which was also obtained with our DFT-based GA. We looked into key elementary reaction steps that are limiting the rate for the formation of CH_4_, CO, and CH_3_OH based on the microkinetic analysis for CO_2_ hydrogenation on the supported Ni_8_-cluster. The forward and backward activation energies for these steps are reported in Table S12, and the structures of IS, TS, and FS are depicted in Table S13. As can be seen from Table S12, the forward and backward activation energies of the Ni_6_ cluster are comparable to those obtained for the Ni_8_-cluster. Based on these results, we constructed a microkinetic model for the Ni_6_-cluster, assuming that all the other elementary reaction steps are the same as for the Ni_8_-cluster. The results highlighted in Figure S13 show that also the Ni_6_ cluster exhibits significant selectivity to CH_3_OH at low temperature (50% at 200 °C). This indicates that the choice of an eight-atom cluster is sufficiently representative for small In_2_O_3_-supported Ni clusters.

We also briefly discuss here our results in comparison to the findings recently reported by Shen et al., who investigated DFT possible pathways of CO_2_ hydrogenation to methanol on Ni_4_/In_2_O_3_ model catalysts.^42^ In line with our work, interfacial oxygen vacancies not only contribute to the adsorption of CO_2_ but also facilitate the hydrogenation of intermediate species to methanol. Shen et al. speculated on the basis of energy diagrams that the formation of CO from CO_2_, followed by its hydrogenation to methanol, is favored over the formate pathway. This is at odds with our finding from microkinetic simulations that CO_2_ hydrogenation to methanol involves formates.

For the Ni_1_-adsorbed system, H_2_ dissociation is heterolytic, resulting in NiH^δ−^ and OH^δ+^ species. On this model, oxygen vacancy formation is endothermic and is furthermore kinetically limited by H_2_O desorption. As oxygen vacancy formation is endothermic, healing the oxygen vacancy is favorable, resulting in a relatively low barrier for direct CO_2_ dissociation into CO and O, with the latter healing the vacancy. This, together with the lower availability of NiH^δ−^ species, results in a preference for CO formation over hydrogenation to methanol, explaining the predicted high CO selectivity. In line with our work, Frei et al. suggested that Ni SA on In_2_O_3_ would be active for the rWGS reaction.^41^

The Ni_1_-doped system also does not lead to methanol. For this model, the abundance of OH^δ+^ species causes high activation energies for the formation of C–H bonds, as also reported in a previous work on In_2_O_3_.^24^ For instance, a barrier of 313 kJ/mol is associated with HCO_2_ hydrogenation to H_2_CO_2_, making methanol formation unfavorable. Furthermore, because of the doped configuration of Ni, there is steric hindrance preventing the carbon atom from effectively bonding to Ni. This prevents the direct involvement of Ni in the catalytic reaction. The dominant pathway on the Ni_1_-doped model features the formation of CO via a redox pathway involving the formation of an oxygen vacancy, the adsorption of CO_2_ on such a vacancy, and subsequent dissociation yielding CO. On this model, the elementary step of CO_2_ dissociation is the investigation of the RDS in the temperature range.

## 4 Conclusions

Using DFT to compute the electronic structure and reaction energy diagrams and construct the input for microkinetic simulations, we investigated the promoting role of Ni on In_2_O_3_ for CO_2_ hydrogenation to methanol. As the exact location and nuclearity of the Ni promoter in Ni/In_2_O_3_ catalysts are unknown, we considered three representative model systems: (i) a single Ni atom doped in the In_2_O_3_(111) surface, (ii) a Ni atom adsorbed on In_2_O_3_(111), and (iii) a small cluster of eight Ni atoms adsorbed on In_2_O_3_(111). With respect to the pristine In_2_O_3_(111) surface, the Ni_8_-cluster model offers a lower overall barrier to oxygen vacancy formation, whereas the Ni_1_-doped and Ni_1_-adsorbed models feature higher overall barriers. Microkinetic simulations reveal that significant methanol formation occurs only for the Ni_8_-cluster model. The metallic cluster allows for facile H_2_ activation, providing hydride (Ni–H) species needed for the formation of oxygen vacancies in In_2_O_3_ and hydrogenation reactions of adsorbed surface intermediates. Methanol synthesis at intermediate temperatures involves the hydrogenation of CO_2_ adsorbed on an oxygen vacancy to a H_2_CO_2_ intermediate (formate pathway). At higher temperatures, the lack of Ni–H species at the surface results in a shift of the selectivity to CO via a mechanism involving a COOH intermediate. On the Ni_8_-cluster model, high barriers associated with either direct or H-assisted CO activation inhibit methane formation. We compared our Ni_8_ cluster with a smaller Ni_6_ cluster also obtained with our DFT-based GA. Our DFT calculations show similar barriers for key rate-limiting steps for the formation of CO, CH_4_, and CH_3_OH for the two clusters. Based on this, we investigated the kinetics of the Ni_6_ cluster with microkinetic modeling and found appreciable selectivity to methanol at low temperatures. Therefore, we conclude that our choice of an eight-atom cluster is sufficiently representative for a small In_2_O_3_-supported Ni cluster. When a single Ni atom is adsorbed on the In_2_O_3_ surface, CO is the main product because a relatively low barrier for direct CO_2_ dissociation is available. When a single Ni atom is doped in the In_2_O_3_ surface, the acidic nature of the H atoms adsorbed on the oxygen anions of In_2_O_3_ results in high activation barriers for CO_2_ hydrogenation reactions, precluding methanol formation.

## Supporting Information Available

Detailed description of the stability of the Ni SA-doped and adsorbed model, an analysis of the structures obtained with the DFT-based GA, electronic structure and Bader charge analysis of Ni/In_2_O_3_ models, full DFT dataset of the mechanisms investigated, geometries of elementary reaction steps, further microkinetic simulations, comparison with a Ni_6_-cluster, and the coordinates of the models (PDF)
