# Supplementary material for: The Promoting Role of Ni on In2O3 for CO2 Hydrogenation to Methanol
Source: ACS Catal. 2023 Jan 18;13(3):1875–92. doi: 10.1021/acscatal.2c04872 (PMC9903295; doi:10.1021/acscatal.2c04872)
Supplement: Supplementary file 1 — cs2c04872_si_001.pdf [file cs2c04872_si_001.pdf]

Supplementary Information

The promoting role of Ni on In_2O_3 for CO_2 hydrogenation to methanol

Francesco Cannizzaro, Emiel J. M. Hensen and Ivo A. W. Filot*

Laboratory of Inorganic Materials and Catalysis, Department of Chemistry and Chemical Engineering, Eindhoven University of Technology, Eindhoven, The Netherlands 5600 MB (NL)

*E-mail: i.a.w.filot@tue.nl

S1: Stability of Ni SA doped in In_2O_3

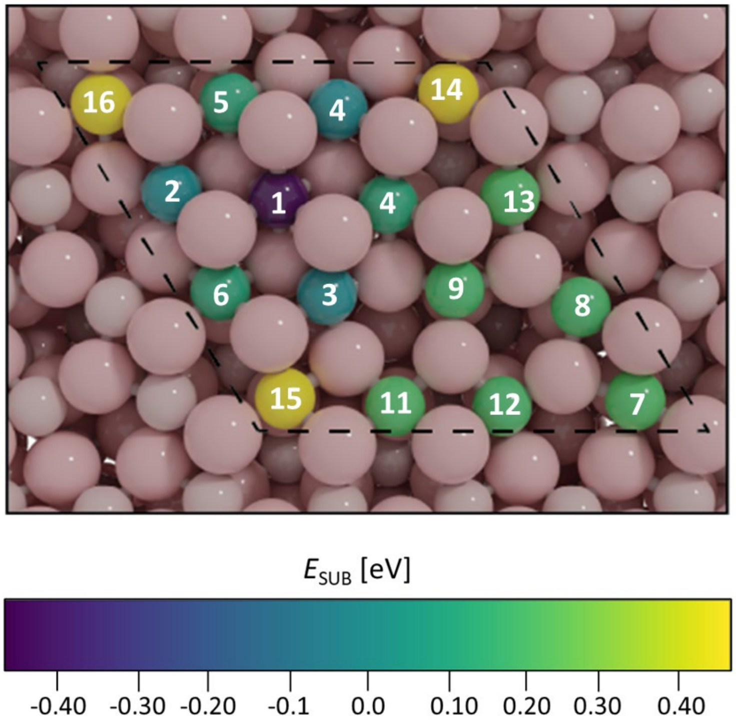

Figure S1. Substitution energy of replacing a surface In atom with a Ni atom. The coloring of atoms at the surface represents the energy associated with their replacement by Ni (E_{sub}, in eV) as can be read in the colorbar. The most stable substitution is indicated by position 1.

Here we have defined the substitution energy E_{SUB}, as:

$$E_{SUB} = E_{Ni/In_2O_3(111)} + E_{In,bulk} - E_{Ni,bulk} - E_{In_2O_3(111)} \quad (S1)$$

where $E_{Ni/In_2O_3(111)}$ is the energy of each Ni_1-doped In_2O_3(111) surface, $E_{In,bulk}$, $E_{Ni,bulk}$ and $E_{In_2O_3(111)}$ are the energies of In bulk, Ni bulk an In_2O_3(111) surface models, respectively. A more negative value indicates a stronger atomic binding.

Table S1. Substitution energy of Ni single atoms inside In_2O_3(111) As shown in Figure S1.

Doping site	E_{sub} [eV]
Ni-1	-0.34573437
Ni-2	-0.19080619
Ni-3	-0.17845143
Ni-4	-0.15901192
Ni-5	-0.04374681
Ni-6	-0.03785749
Ni-7	0.03865669
Ni-8	0.04338165
Ni-9	0.06744435
Ni-10	0.17168803
Ni-11	0.35099871
Ni-12	0.43707849
Ni-13	0.59568647
Ni-14	0.60240986
Ni-15	0.60681143
Ni-16	0.61352356

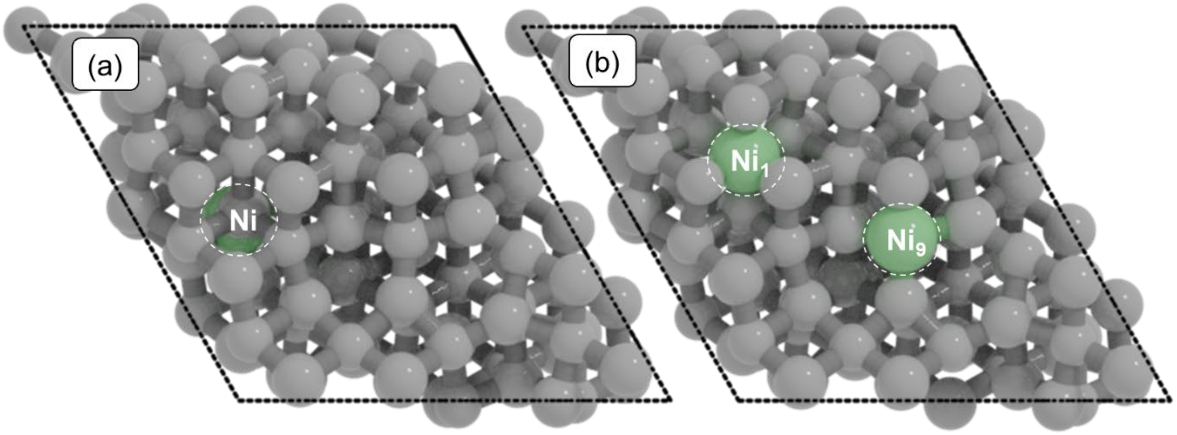

Figure S2. (a) Ni atom doped in the In_2O_3 bulk, one layer underneath the surface. (b) two Ni atoms doped on different positions labeled as in Figure S1. Ni atoms are highlighted in green, all the other atoms are in grey.cd .

S2: Stability of Ni single atom adsorbed on top of In_2O_3

Table S2. Adsorption energy of adsorbed single atoms on In_2O_3(111).

Adsorption site	E_{ads} [eV]
A1	-5.17
A2	-5.17
A3	-4.96
A4	-4.85
A5	-4.17
A6	-4.20

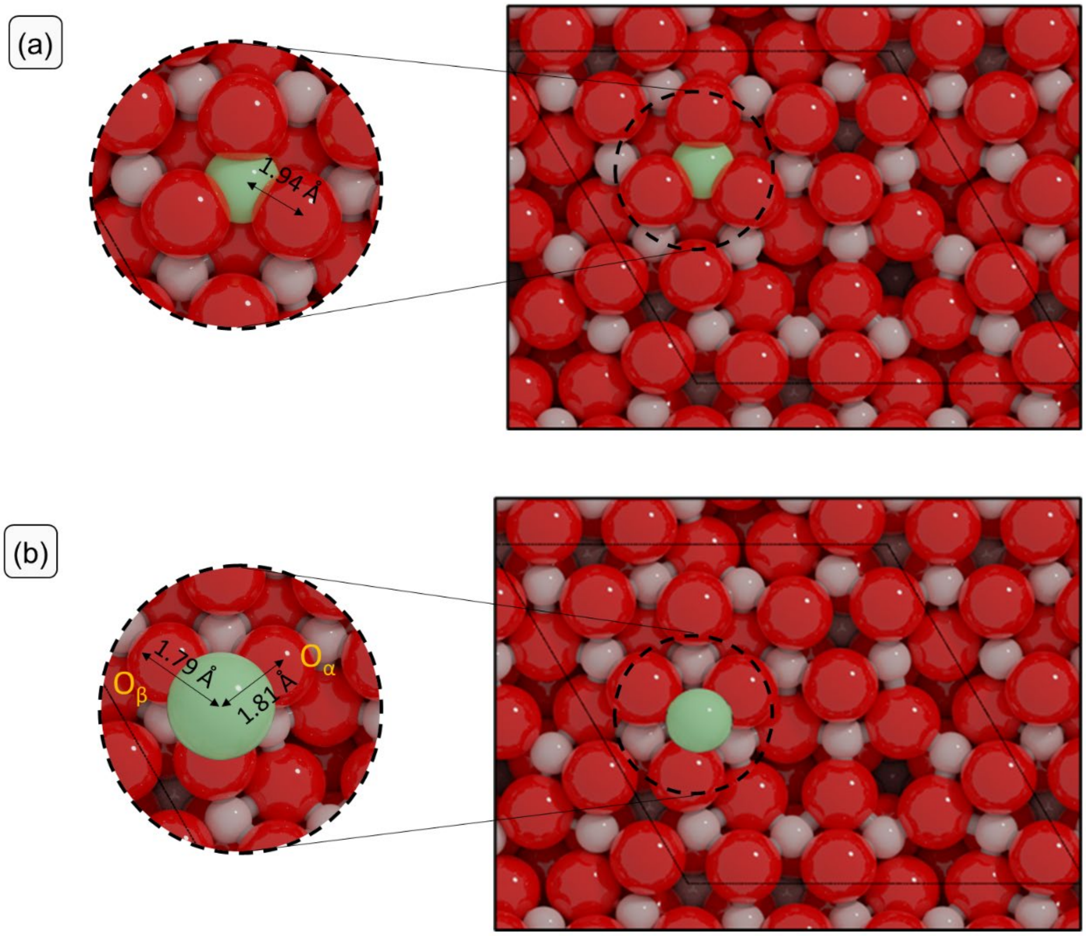

Figure S3. Most stable structures for (a) SA doped and (b) SA adsorbed. The Ni-O distances are depicted on the left.

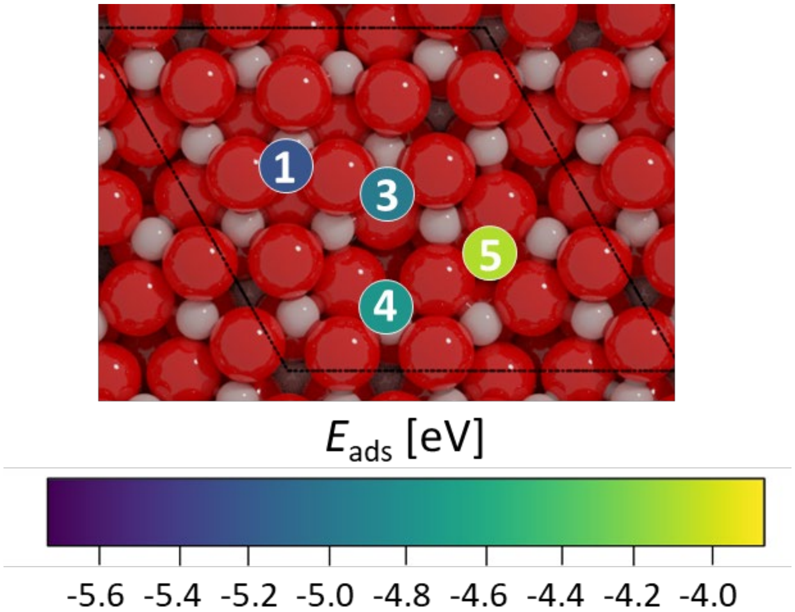

Figure S4. Adsorption sites used to study the migration of Ni single atoms on the In_2O_3(111) surface.

Table S3. Migration barriers of adsorbed single atoms on In_2O_3(111). The positions are shown in Figure S4.

	E_a [kj/mol]	E_R [eV]
Pos1 → Pos3	95	21
Pos3→ Pos4	165	11
Pos4 → Pos5	142	65

S3. Analysis of GA-obtained Ni/In_2O_3 clusters

S3.1 Metal support interactions of Ni/In_2O_3 clusters

Table S4. Cohesive energies for gas-phase ($E_{COH,\ free}$) and supported ($E_{COH,\ supp}$) Ni_8- and Ni_6-In_2O_3 models.

**Model**	**$E_{COH,\ free}$ [kJ/mol/atom]**	**$E_{COH,\ supp}$[kJ/mol/atom]**	**Diff [kj/mol/atom]**
**Ni_6**	249	360	111
**Ni_8**	268	358	90

Table S5. Activation and reaction energies (in kJ/mol) for the removal of a single Ni atom from supported Ni_8- and Ni_6-In_2O_3 models.

**Model**	**E_a [kJ/mol]**	**E_R [kJ/mol]**
**Ni_6/In_2O_3**	201	179
**Ni_8/In_2O_3**	232	192

Table S6. Initial, transition and final states the calculations as reported in Table S5.

**Ni_8/In_2O_3**			**Ni_6/In_2O_3**		
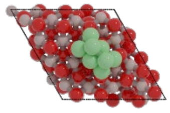	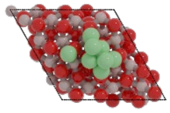	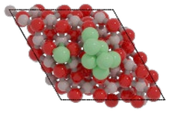	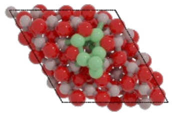	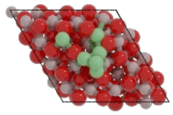	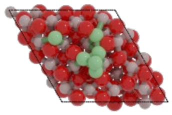

S3.2 Structure analysis of GA-obtained Ni-In_2O_3 clusters

To assess the probability to find a structure different than the energy minimum one, we performed an analysis based on the Boltzmann probability distribution as given by:

$$\frac{p_i}{p_j} = e^{\frac{\epsilon_j - \epsilon_i}{kT}} \tag{S2}$$

where p_i is the probability of the system being in state i, ϵ_i is the energy of that state, ϵ_j the energy of the energy minimum structure, and kT is the product of Boltzmann's constant k and temperature T. This function gives the probability that the system will be in a certain state (e.g., a given cluster configuration) as a function of the energy of that state and the temperature. The result of this analysis for In_2O_3-supported Ni_8 and a Ni_6 clusters are given in Figure S5a and Figure S5c, respectively. Herein, we focus on candidate structures having a relative probability of at least 10% with respect to the most stable cluster in the population. As can be seen from Figure S5a, we identified only one Ni_8-cluster structure (cand_2 in Figure S5b) that has a sufficiently large relative probability at 400 K other than the energy minimum one. Similarly, for Ni_6-In_2O_3, only one structure other than the energy minimum one has a probability of at least 10% at 400 K (cand_2 in Figure S5d).

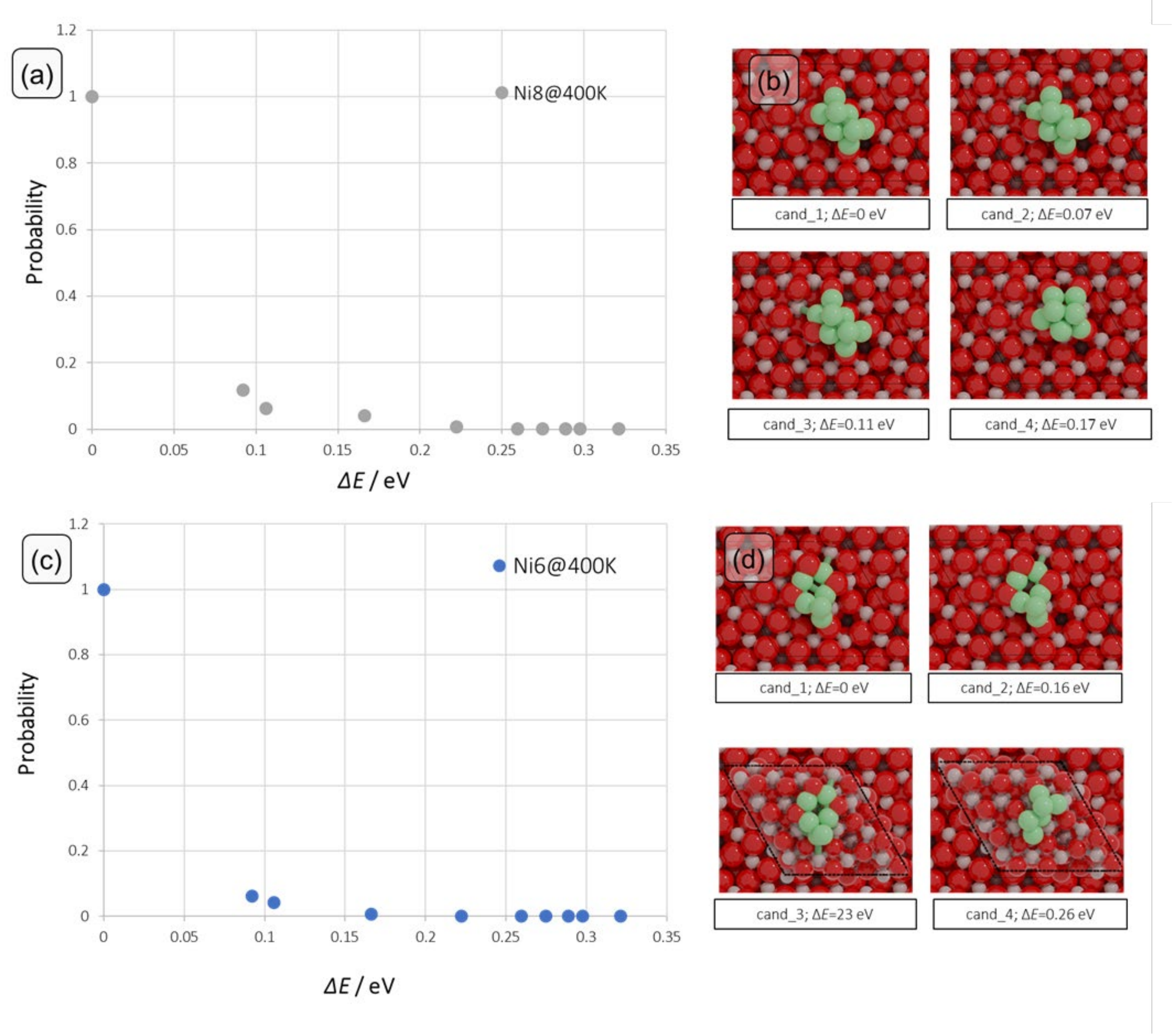

Figure S5. (a), (c) Boltzmann probability distribution of the candidate structures for In_2O_3-supported Ni_8 and Ni_6 clusters, respectively. (b, (d) top-view of the candidate structures with at least 10% probability and their energy relative to the energy minimum for In_2O_3-supported Ni_8 and Ni_6 clusters, respectively.

To perform a similarity analysis of the GA-obtained structures, a distance matrix $\mathbf{A}(i)$ for each cluster was produced wherein each matrix element represents the distance between any two atoms in a single cluster. The similarity between any two clusters in the set can then be expressed as the minimum Hilbert-Schmidt (HS) norm of the difference of their distance matrices, wherein the minimum is established by evaluating all possible permutations over the indices for one of the distance matrices. The minimized HS norm (mHS) for a pair of clusters ($\mathbf{A}(i)$, $\mathbf{A}(j)$) is given by

$$d_{ij} = \min\left\|\mathbf{HA}(i)\mathbf{H}^{\mathrm{T}} - \mathbf{A}(j)\right\|_F \quad \text{(S3)}$$

where $\|\cdot\|_F$ is the Frobenius norm and $\mathbf{H}$ is a permutation over the indices for one of the distance matrices. Table S7 collects the results of the minimum Hilbert-Schmidt norm of the distance matrix for the structures reported in Figure S5. As can be seen from Table S7, for the Ni_8-cluster, the mHS value between the minimum energy structure and cand_2 amounts to 0.64. Concerning a Ni_6-cluster, a value of 0.18 is found. We can better understand this value by calculating a hypothetical minimized HS norm of an 8-atom cluster with a perturbation of 0.1 Å per atom with respect to an arbitrary cluster configuration. The minimized HS norm (equation S3) is then given by $\|\mathrm{A}\|_{HS} = \sqrt{N^2} * 0.1 = 0.8$. Thus, a value of the HS norm of 0.64 indicates that the coordinates of the atoms in the two clusters differ, on average, less than 0.1 Å. From a careful visual inspection of the cand_1 and cand_2 structures for both Ni_6 and Ni_8 clusters, we infer that these clusters have identical structure, and that CO_2 hydrogenation would likely follow similar reaction pathways.

Table S7. Minimum Hilbert-Schmidt norm of the distance-matrix for the structures reported in Figure S5. Cand_1 corresponds to the energy minimum structure. The first two lines report the results for the Ni_8-In_2O_3 model, while the last two lines report the results for the Ni_6-In_2O_3 model.

**Ni_8**	**Cand_1**	**Cand_2**	**Cand_3**	**Cand_4**
**Cand_1**	0	0.64	0.80	4.79
**Ni_6**				
**Cand_1**	0	0.18	0.91	3.83

S4: Electronic structure analysis of Ni/In_2O_3 model catalyst

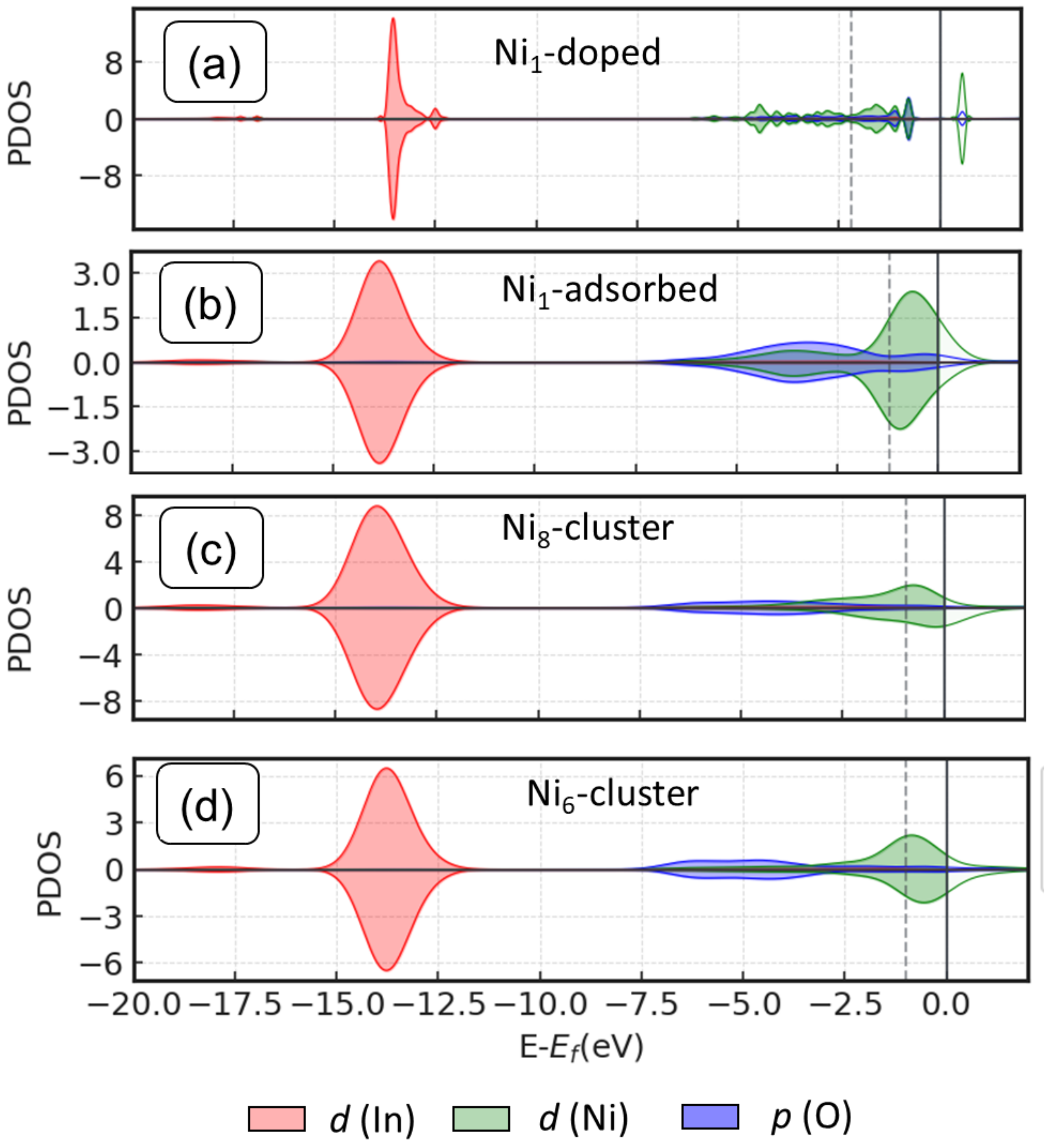

Figure S6. Partial density of states (PDOS) of Ni 3*d*, O 2*p* and In 4*d* orbitals (the O atom adjacent to Ni) in (a) Ni_1-doped, (b) Ni_1-adsorbed and (c) Ni_8- and (d) Ni_6-cluster. The *d*-band center is shown with a dashed grey line. The Fermi level is set at zero.

S5. Bader Charge analysis of the Ni_8/In_2O_3 model

Table S8. Bader charges of stoichiometric and oxygen-defective $Ni_8/In_2O_3(111)$ surfaces.

Cluster	q[Nix] /|e|
Ni_8-cluster stoichiometric	1.07
Ni_8-cluster with one Ov	0.67

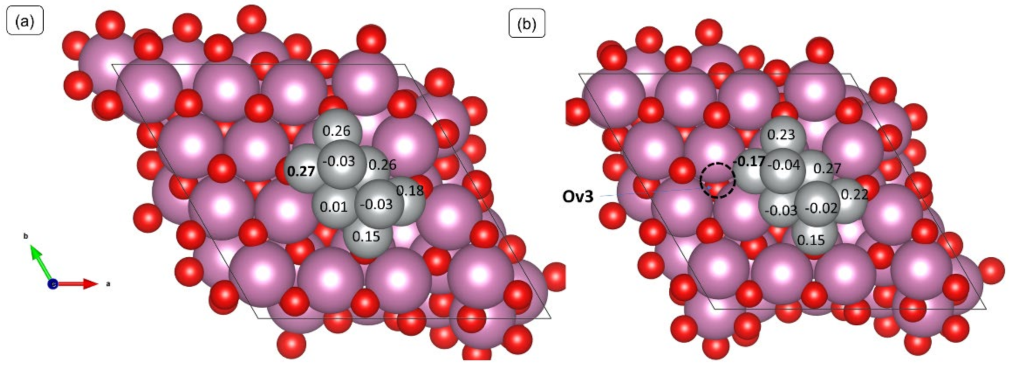

Figure S7. Bader charge analysis of (a) Ni_8-cluster stoichiometric and (b) Ni_8-cluster with one oxygen vacancy (Ov3) indicated by a dashed circle. Upon formation of an oxygen vacancy, the excess charge is redistributed towards the closest Ni atom from the cluster.

S6: pCOHP analysis of O bonding on Ni/In_2O_3

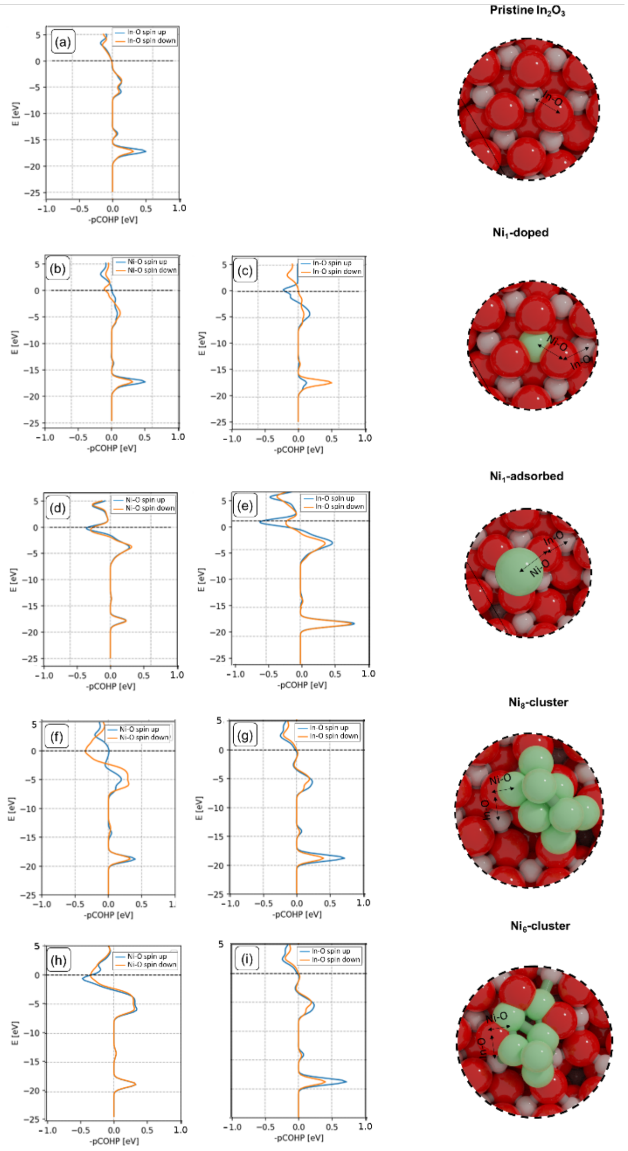

Figure S8. Projected cystal hamiltonian population (pCOHP) analysis of Ni-O and In-O bonds. (a) In-O interactions for a surface oxygen on bare In_2O_3. (b-c) Ni-O and In-O interactions for a surface oxygen atom bonding the Ni_1-doped (b-c) and Ni_1-adsorbed (d-e), respectively. (f-g) Ni-O and In-O interactions for a surface oxygen atom at the Ni/In_2O_3 interface in the Ni_8-cluster model. Positive (negative) pCOHP values indicate (anti)bonding interactions.The Fermi level is set at zero. The structures on the right show the interactions taken into account for each model surface.

S7. DFT Tables

Table S9a. Kinetic parameters for CO_2 hydrogenation to CH_3OH, CO and H_2O over Ni_1-doped In_2O_3 model catalyst

	Elementary steps	Q_{TS}/Q_{IS}	Q_{TS}/Q_{FS}	$E_{act,f}$ (kJ/mol)	$E_{act,b}$ (kJ/mol)
ID	Oxygen vacancy formation pathway				
1	$H_2(g) + 2* \rightleftharpoons 2H^*$	2.51	1.79	70	364
2	$2OH^* \rightleftharpoons H_2O^* + O^*$	0.19	2.91	164	27
3	$H_2O^* \rightleftharpoons H_2O(g) + *$	-	-	80	-
	Formate pathway				
4	$CO_2(g) + * \rightleftharpoons CO_2^*$	1.0	1.0	103	-
5	$CO_2^* + H_2(g) + 2* \rightleftharpoons CO_2^* + 2H^*$	2.51	1.79	104	126
6	$CO_2^* + 2H^* \rightleftharpoons HCO_2{}^* + H^* + *$	0.086	0.47	165	158
7	$HCO_2{}^* + H^* \rightleftharpoons H_2CO_2{}^* + *$	0.05	0.93	313	26
8	$H_2CO_2{}^* + H_2(g) + 2* \rightleftharpoons H_2CO_2^* + 2H^*$	2.51	1.79	71	102
9	$H_2CO_2^* + 2H^* \rightleftharpoons H_2CO_2H^* + H^*$	2.64	1.82	49	7
10	$H_2CO_2H^* + H^* \rightleftharpoons CH_2O^* + H^* + OH^*$	1.00	1.00	97	237
11	$CH_2O^* + OH^* + H^* \rightleftharpoons CH_3O^* + O^* + H^*$	3.453	2.16	117	69
12	$CH_3O^* + H^* \rightleftharpoons CH_3OH(g)$	0.728	4.30	102	121
13	$Ni\# \rightleftharpoons Ni$	1.0	1.0	120	35
	rWGS pathway				
14	$CO_2^* \rightleftharpoons CO(g) + O^*$	-	-	230	-

Activation energies (E_{act}) were directly obtained from DFT calculations.

Pre-exponential factors were estimated by transition state theory at T = 550 K.

Elementary steps ID correspond to Figure 3a

Table S9b. Kinetic parameters for CO_2 hydrogenation to CH_3OH, CO and H_2O over Ni_1-adsorbed In_2O_3 model catalyst.

	Elementary steps	Q_{TS}/Q_{IS}	Q_{TS}/Q_{FS}	$E_{act,f}$ (kJ/mol)	$E_{act,b}$ (kJ/mol)
ID	Oxygen vacancy formation pathway				
1	$H_2(g) + 2* \rightleftharpoons 2H^*$	0.20	0.57	19	55
2	$H^* + OH^* \rightleftharpoons H_2O^* + O^*$	1.70	0.39	93	66
3	$H_2O^* \rightleftharpoons H_2O(g) + *$	-	-	177	-
	Formate pathway				
4	$CO_{2(g)} + * \rightleftharpoons CO_2^*$	1.0	1.0	144	-
5	$CO_2^* + H_2(g) + 2* \rightleftharpoons CO_2^* + 2H^*$	0.20	0.57	19	55
6	$CO_2^* + 2H^* \rightleftharpoons HCO_2{}^* + H^* + *$	5.55	0.59	66	68
7	$HCO_2{}^* + H^* \rightleftharpoons H_2CO_2{}^* + *$	0.17	1.44	114	34
8	$H_2CO_2{}^* + * \rightleftharpoons CH_2O^* + O^*$	1.05	0.28	101	33
9	$CH_2O^* + H_2(g) + 2* \rightleftharpoons CH_2O^* + 2H^*$	0.20	0.57	19	55
10	$CH_2O^* + 2H^* \rightleftharpoons CH_3O^* + H^*$	0.47	0.54	55	132
11	$CH_3O^* + H^* \rightleftharpoons CH_3OH_{(g)}$	0.06	0.61	61	54
	CO hydrogenation pathway				
12	$CO^*{}_2 + * \rightleftharpoons CO^* + O^*$	0.52	0.17	81	78
13	$CO^* + H_2(g) + 2* \rightleftharpoons CO^* + 2H^*$	0.20	0.57	19	55
14	$CO^* + 2H^* \rightleftharpoons HCO^* + H^*$	0.52	0.17	71	65
15	$HCO^* + H^* \rightleftharpoons CH_2O^* + *$	0.20	0.57	177	35
	rWGS pathway				
16	$CO^*{}_2 + 2H^* \rightleftharpoons COOH^* + H^* + *$	0.31	0.02	82	69
17	$COOH^* + * \rightleftharpoons CO^* + OH^*$	1.13	0.33	42	25
18	$CO^* + OH^* + H^* \rightleftharpoons CO^* + H_2O^*$	1.51	0.96	140	198
19	$CO^* \rightleftharpoons CO(g) + Ov$	-	-	51	-
20	$CO^* \rightleftharpoons CO(g) + *$	-	-	121	-

Activation energies (E_{act}) were directly obtained from DFT calculations.

Pre-exponential factors were estimated by transition state theory at T = 550 K.

Elementary steps ID correspond to Figure 3b

Table S9c. Kinetic parameters for CO_2 hydrogenation to CH_3OH, CO and H_2O over Ni_8/In_2O_3 model catalyst.

	Elementary steps	Q_{TS}/Q_{IS}	Q_{TS}/Q_{FS}	$E_{act,f}$ (kJ/mol)	$E_{act,b}$ (kJ/mol)
ID	Oxygen vacancy formation pathway				
1	$H_2(g) + 2* \rightleftharpoons 2H^*$	2.51	1.79	0	89
2	$2H^* + O \rightleftharpoons H^* + OH^*$	2.93	0.51	152	146
3	$H^* + OH^* \rightleftharpoons H_2O^* + *$	0.13	0.40	140	172
4	$H_2O^* \rightleftharpoons H_2O(g) + *$	-	-	80	-
	Formate pathway				
5	$CO_{2(g)} + * \rightleftharpoons CO_2^*$	1.0	1.0	144	-
6	$CO_2^* + H_2(g) + 2* \rightleftharpoons CO_2^* + 2H^*$	2.51	1.79	0	89
7	$CO_2^* + 2H^* \rightleftharpoons HCO_2^* + H^* + *$	1.01	0.9	95	116
8	$HCO_2^* + H^* \rightleftharpoons H_2CO_2^*$	0.56	0.85	177	39
9	$H_2CO_2^* + * \rightleftharpoons CH_2O^* + O^*$	2.64	1.82	52	71
10	$CH_2O^* + H_2(g) + 2* \rightleftharpoons CH_2O^* + 2H^*$	2.51	1.79	0	89
11	$CH_2O^* + 2H^* \rightleftharpoons CH_3O^* + H^* + *$	1.61	4.78	83	124
12	$CH_3O^* + H^* \rightleftharpoons CH_3OH^* + *$	0.82	1.31	124	94
13	$H_3COH^* \rightleftharpoons H_3COH(g) + *$	-	-	35	-
	CO hydrogenation pathway				
14	$CO^*_2 + * \rightleftharpoons CO^* + O^*$	1.72	0.75	114	144
15	$CO^* + H_2(g) + 2* \rightleftharpoons CO^* + 2H^*$	2.51	1.79	0	89
16	$CO^* + 2H^* \rightleftharpoons HCO^* + H^{*+*}$	1.3	1.1	150	119
17	$CO^* + 2H^* \rightleftharpoons COH^* + H^* + *$	1.2	0.54	235	120
18	$HCO^* + H^* \rightleftharpoons HCOH^* + *$	0.48	0.90	69	39
19	$HCO^* + H^* \rightleftharpoons CH_2O^* + *$	0.48	0.51	165	10
20	$COH^* + H^* \rightleftharpoons HCOH^* + *$	0.48	0.90	211	99
21	$HCOH^* + H_2(g) + 2* \rightleftharpoons HCOH^* + 2H^*$	2.51	1.79	0	89
22	$HCOH^* + 2H^* \rightleftharpoons CH_2OH^* + H^*$	0.67	3.13	111	61
23	$CH_2OH^* + H^* \rightleftharpoons CH_3OH^* + *$	1.0	1.0	76	128
	rWGS pathway				
24	$CO^*_2 + 2H^* \rightleftharpoons COOH^* + H^* + *$	0.73	0.89	62	14
25	$COOH^* + * \rightleftharpoons CO^* + OH^*$	7.32	3.55	66	125
26	$CO^* + OH^* + H^* \rightleftharpoons H_2O^* + CO^{*+*}$	0.67	0.67	122	172
27	$CO^* \rightleftharpoons CO(g) + Ov$	-	-	114	-
28	$CO^* \rightleftharpoons CO(g) + *$	-	-	130	-

Activation energies (E_{act}) were directly obtained from DFT calculations.

Pre-exponential factors were estimated by transition state theory at T = 550 K.

Elementary steps ID correspond to Figure 3c

S8. Geometries of elementary reaction steps on Ni_1-doped model

1. $\mathbf{H_2 + 2 * \rightleftharpoons 2H^*}$	2. $H^* + OH^* \rightleftharpoons H_2O\,^*$
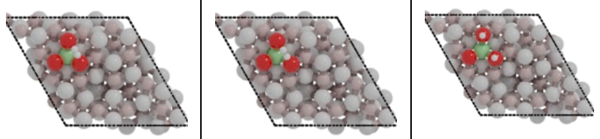	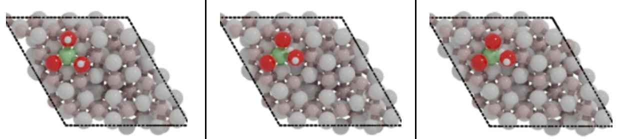
3. $\mathbf{H_2O + * \rightleftharpoons H_2O\,^*}$	4. $CO_2 \quad + * \rightleftharpoons CO_2^* +$
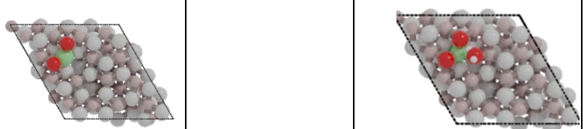	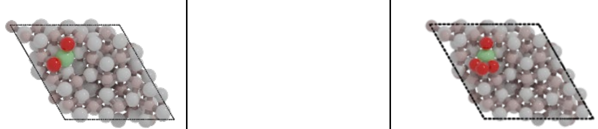
**5.** $\mathbf{CO_2^* + \ 2 * \ + H_2 \ \rightleftharpoons CO_2^* + 2H}$	**6.** $CO_2^* \quad + 2H^* \rightleftharpoons HCO_2^* + \ H^*$
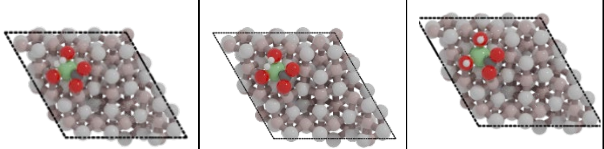	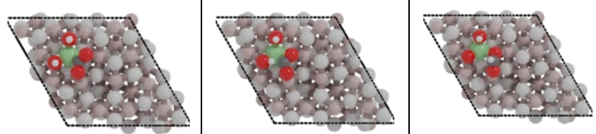
7. $\mathbf{HCO_2^* + H^* \rightleftharpoons H_2CO_2^* + *}$	8. $H_2CO_2^* + H_2(g) + 2 * \rightleftharpoons H_2CO_2^* + \ 2H^*$
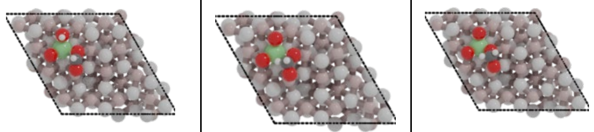	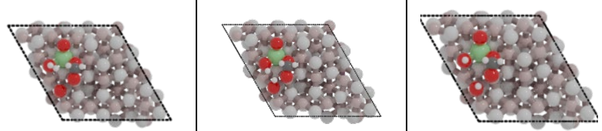
9. $\mathbf{H_2CO_2^* + 2H^* \rightleftharpoons H_2CO_2\ H^* + \ H^*}$	10. $H_2CO_2\ H + * + H^* \ \rightleftharpoons H_2CO^* + \ OH^* \ + H^*$
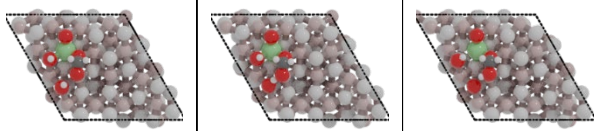	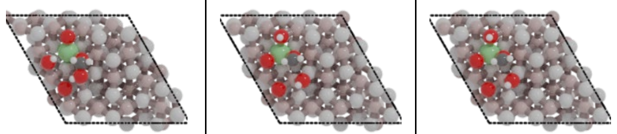
11. $\mathbf{CH_2O^* + H^* + OH^* \rightleftharpoons CH_3O^* \ + * \quad OH^*}$	12. $CH_3O^* + H^* \ \rightleftharpoons CH_3OH(g) \quad + *$

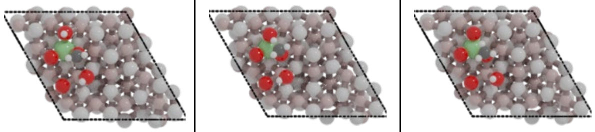	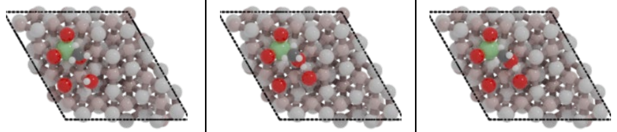
13. $\mathbf{Ni\#} \rightleftharpoons \mathbf{Ni}$	14. $CO_2^* + * \rightleftharpoons CO(g) + O^*$
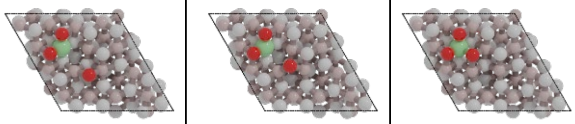	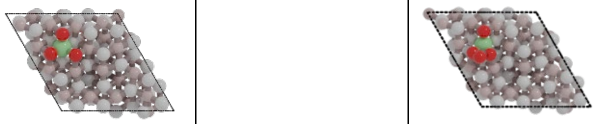

S9. Geometries of elementary reaction steps on Ni_1-adsorbed model

1. $\mathbf{H_2 + 2 * \rightleftharpoons 2H^*}$	2. $H^* + OH^* \rightleftharpoons H_2O^*$
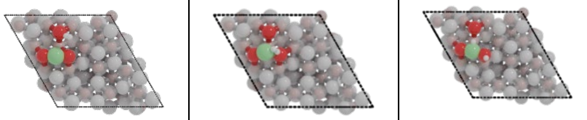	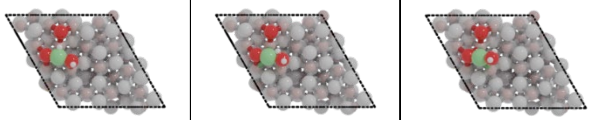
**3.** $\mathbf{H_2O + * \rightleftharpoons H_2O^*}$	**4.** $CO_2(g) + * \rightleftharpoons CO_2^* +$
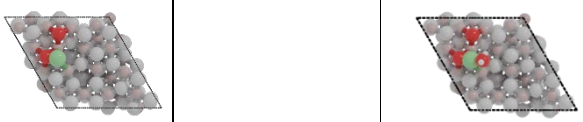	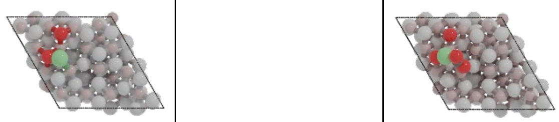
**5.** $\mathbf{CO_2^* + 2 * + H_2 \rightleftharpoons CO_2^* + 2H}$	**6.** $CO_2^* + 2H^* \rightleftharpoons HCO_2^* + * + H^*$
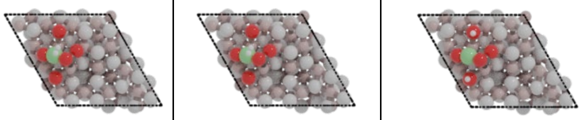	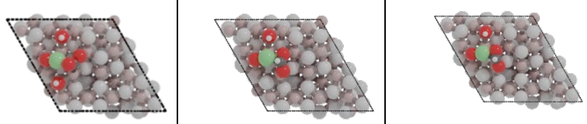

7. $\mathbf{HCO_2^* + H^* \rightleftharpoons H_2CO_2{}^* + *}$

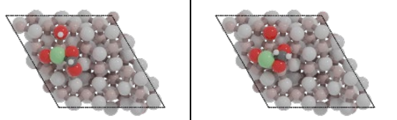

8. $H_2CO_2{}^* \rightleftharpoons H_2CO^* + O^*$

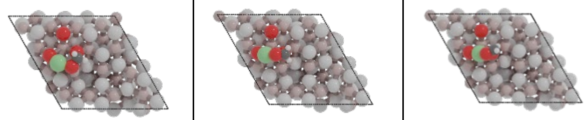

9. $\mathbf{CH_2O^* + H_2(g) + 2\,* \rightleftharpoons CH_2O^* + 2H^*}$

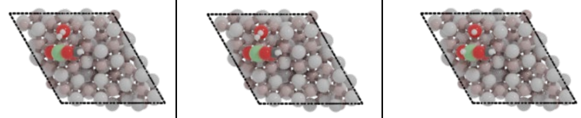

10. $CH_2O^* + H^* \rightleftharpoons CH_3O^* + *$

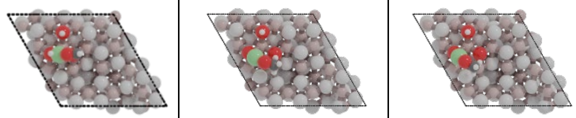

11. $\mathbf{CH_3O^* + H^* \rightleftharpoons CH_3OH(g) + *}$

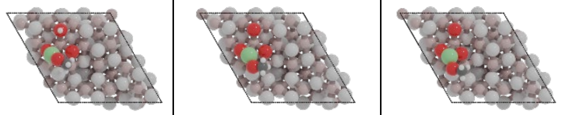

12. $\mathbf{CO^*{}_2 + * \rightleftharpoons CO^* + O^*}$

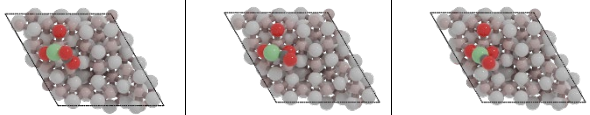

13. $CO^* + H_2(g) + 2\,* \rightleftharpoons CO^* + 2H^*$

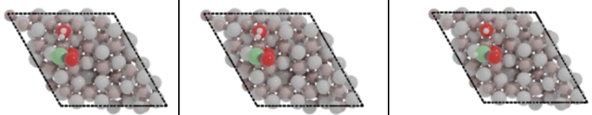

14. $\mathbf{CO^* + 2H^* \rightleftharpoons HCO^* + H^*}$

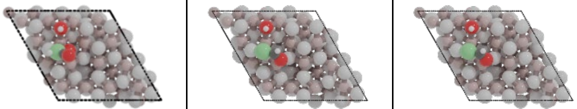

15. $HCO^* + H^* \rightleftharpoons H_2CO^* + *$

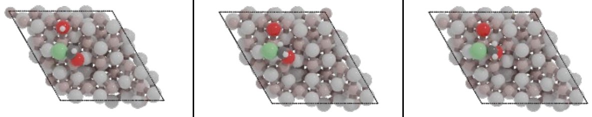

16. $\mathbf{CO_2^* + H^* \rightleftharpoons COOH^* + *}$

17. $COOH^* + * \rightleftharpoons CO^* + OH^*$

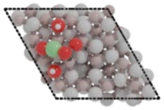	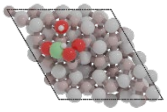	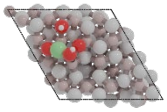	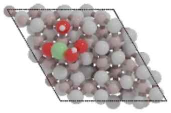	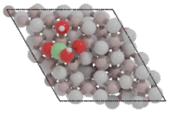	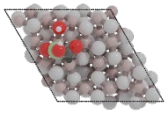
18. $\mathbf{CO^* + OH^* + H^* \rightleftharpoons H_2O^* + CO^*}$			19. $CO^* \rightleftharpoons CO(g) \ + \ Ov$		
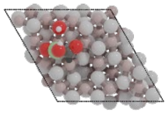	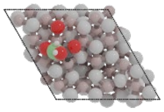	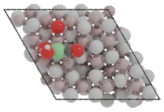	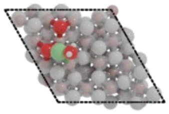		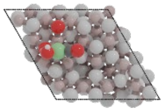
20. $\mathbf{CO^* \rightleftharpoons CO(g) \ + *}$					
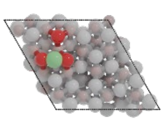		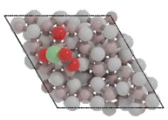			

S10. Geometries of elementary reaction steps on Ni_8-cluster model

1. $\mathbf{H_2 + 2 * \rightleftharpoons 2H^*}$			2. $2H^* + O \ \rightleftharpoons H^* + OH^*$		
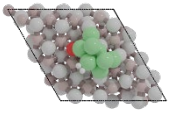	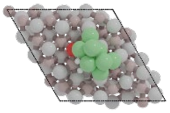	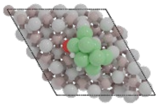	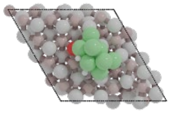	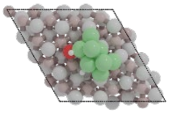	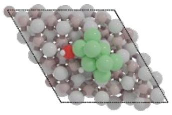
3. $\mathbf{H^* + OH^* \ \rightleftharpoons H_2O\ ^*}$			4. $H_2O + * \rightleftharpoons H_2O\ ^*$		
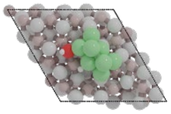	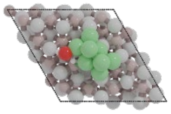	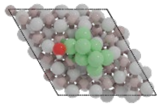	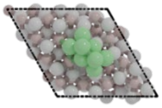		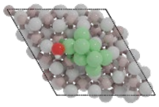
5. $\mathbf{CO_2 \ + * \rightleftharpoons CO_2^* +}$			6. $CO_2^* \ + H_2(g) \rightleftharpoons HCOO^* + \ 2H^*$		

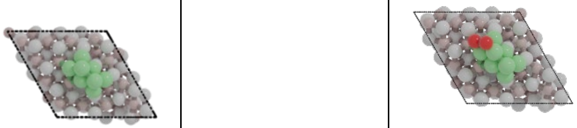

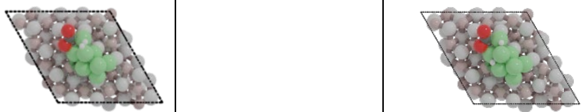

7. $\mathbf{CO_2^*} + \mathbf{H^*} \rightleftharpoons \mathbf{HCOO^*} + *$

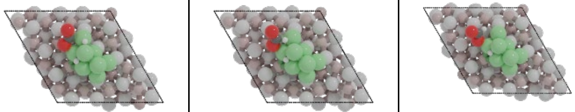

8. $HCOO^* + 2H^* \rightleftharpoons H_2COO^* + H^* + *$

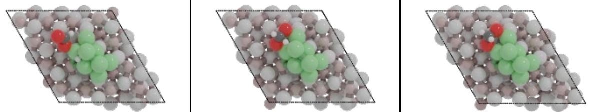

9. $\mathbf{H_2COO^*} \rightleftharpoons \mathbf{H_2CO^*} + \mathbf{O^*}$

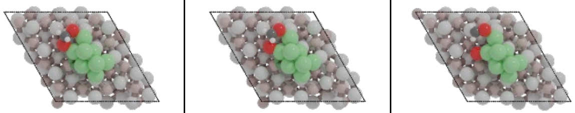

10. $H_2CO^* + H_2(g) + 2* \rightleftharpoons H_2CO^* + 2H^*$

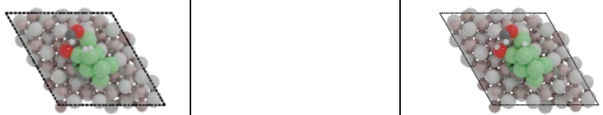

11. $\mathbf{H_2CO^*} + \mathbf{2H^*} \rightleftharpoons \mathbf{H_3CO^*} + \mathbf{H^*} + *$

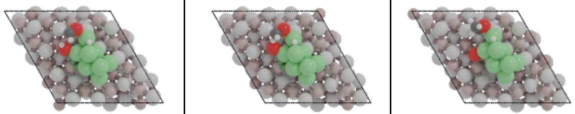

12. $H_3CO^* + H^* \rightleftharpoons H_3COH^*$

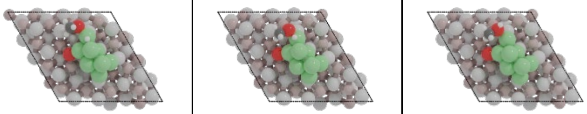

13. $\mathbf{H_3COH^*} \rightleftharpoons \mathbf{H_3COH(g)} + *$

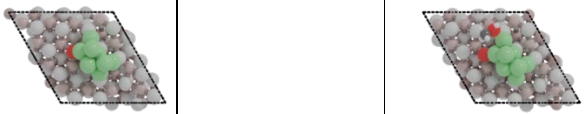

14. $CO^*_2 + * \rightleftharpoons CO^* + O^*$

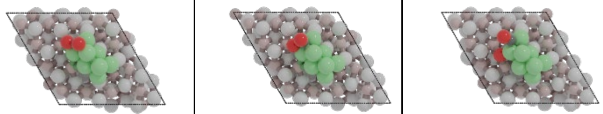

15. $\mathbf{CO^*_2} + \mathbf{H_2(g)} + \mathbf{2} * \rightleftharpoons \mathbf{CO^*} + \mathbf{2H^*}$

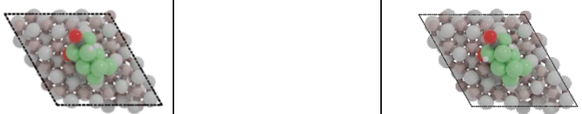

16. $CO^* + 2H^* \rightleftharpoons HCO^* + H^*$

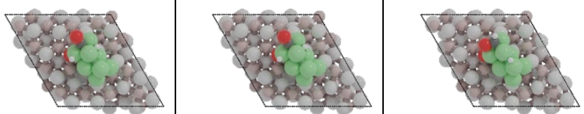

17. $\mathbf{CO^* + 2H^* \rightleftharpoons COH^* + H^*}$			18. $\mathrm{HCO^* + H^* \rightleftharpoons HCOH^* + *}$		
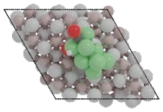	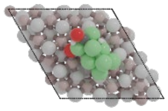	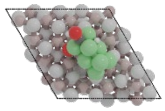	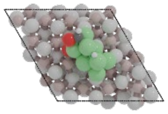	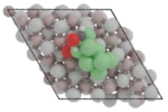	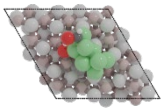
19. $\mathbf{HCO^* + H^* \rightleftharpoons H_2CO^* + *}$			20. $\mathrm{COH^* + H^* \rightleftharpoons HCOH^* + *}$		
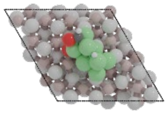	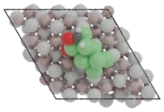	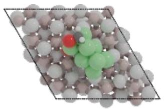	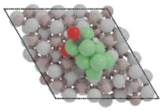	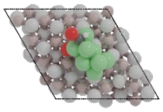	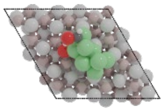
21. $\mathbf{HCOH^* + H_2(g) + 2 * \rightleftharpoons H_2COH^* + 2H^*}$			22. $\mathrm{HCOH^* + H^* \rightleftharpoons H_2COH^* + *}$		
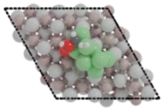		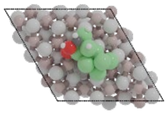	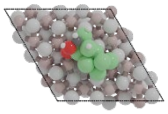	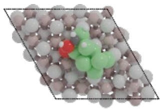	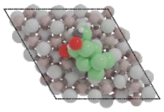
23. $\mathbf{H_2COH^* + H^* \rightleftharpoons H_3COH^* + *}$					
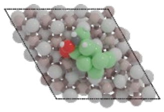	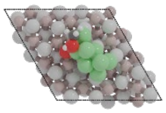	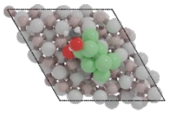			
24. $\mathbf{CO_2^* + H^* \rightleftharpoons COOH^* + *}$			25. $\mathrm{COOH^* + * \rightleftharpoons CO^* + OH^*}$		
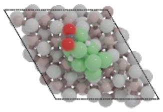	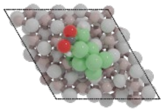	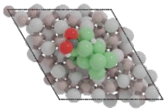	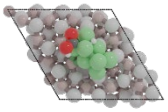	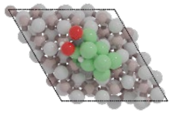	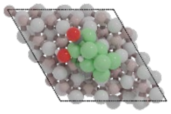
26. $\mathbf{CO^* + OH^* + H^* \rightleftharpoons H_2O^* + CO^*}$			27. $\mathrm{CO^* + H_2O(g) \rightleftharpoons * + CO(g) + H_2O^*}$		
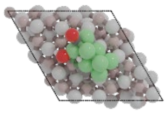	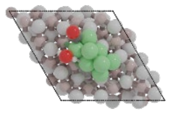	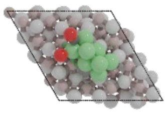	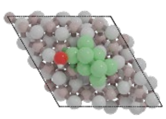		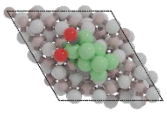

Table S10. Forward and backward activation energies (in kJ/mol) for CO activation elementary steps. A comparison with Sterk et al.[1] is included.

Elementary Reaction Step	E_a	E_b	Ref
$\mathbf{CO^*(3f) + * \rightleftarrows C^*(3f) + O^*(3f)}$	312	265	This work
$\mathbf{CO^*(2f) + * \rightleftarrows C^*(3f) + O^*(3f)}$	231	135	This work
$\mathbf{CO^*(3f) + * \rightleftarrows C^*(3f) + O^*(In)}$	241	75	This work
$\mathbf{COH^* + * \rightleftarrows HC^* + O^*}$	N.A.	N.A.	This work
$\mathbf{HCO^* + * \rightleftarrows HC^* + O^*}$	174	34	This work
$\mathbf{H_2CO^* + * \rightleftarrows H_2C^* + O^*}$	N.A.	N.A.	This work
$\mathbf{H_3CO^* + * \rightleftarrows H_3C^* + O^*}$	N.A.	N.A.	This work
$\mathbf{CO^* + * \rightleftarrows C^* + O^*}$	150	99	Ni(110)[1]
$\mathbf{COH^* + * \rightleftarrows HC^* + O^*}$	154	32.	Ni(110)[1]
$\mathbf{HCO^* + * \rightleftarrows HC^* + O^*}$	117	31	Ni(110)[1]
$\mathbf{H_2CO^* + * \rightleftarrows H_2C^* + O^*}$	82	68	Ni(110)[1]
$\mathbf{H_3CO^* + * \rightleftarrows H_3C^* + O^*}$	182	198	Ni(110)[1]

Table S11. Structures of IS, TS and FS for the CO dissociation elementary reaction steps

$\mathbf{CO^*(3f) + * \rightleftarrows C^*(3f) + O^*(3f)}$			$\mathbf{CO^*(2f) + * \rightleftarrows C^*(3f) + O^*(3f)}$		
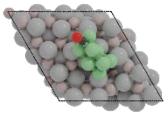	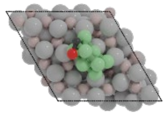	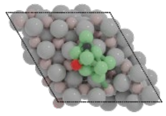	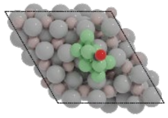	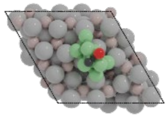	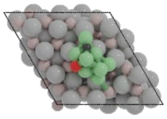
$\mathbf{CO^*(3f) + * \rightleftarrows C^*(3f) + O^*(In)}$			$\mathbf{HCO^* + * \rightleftarrows HC^* + O^*}$		
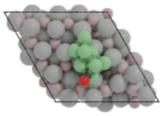	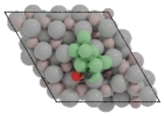	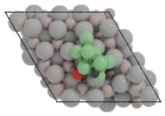	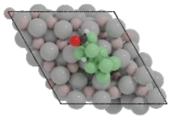	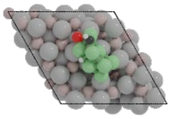	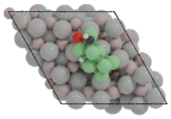

S11. Further microkinetic simulations

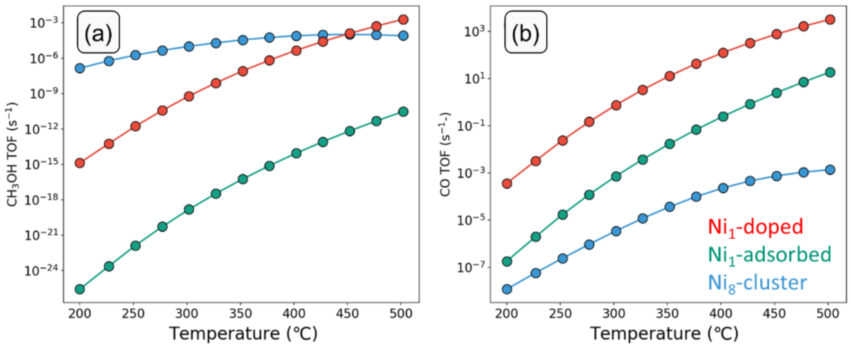

Figure S9. (a) TOF to CH_3OH (s^{-1}) and (b) TOF to CO (s^{-1}) as a function of temperature obtained by the microkinetic model for the three Ni/In_2O_3 model catalysts.

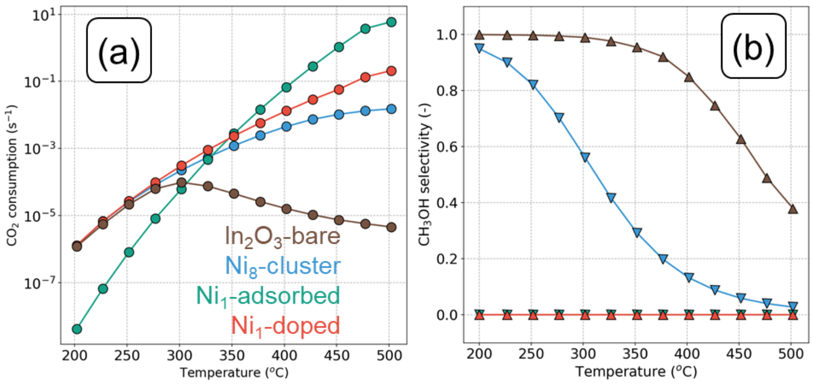

Figure S10. (a) CO_2 consumption rate (s^{-1}) and (b) CH_3OH selectivity as a function of temperature on different models (p = 50 bar, H_2/CO_2 ratio =5). The data for In_2O_3-bare are taken from reference [2]

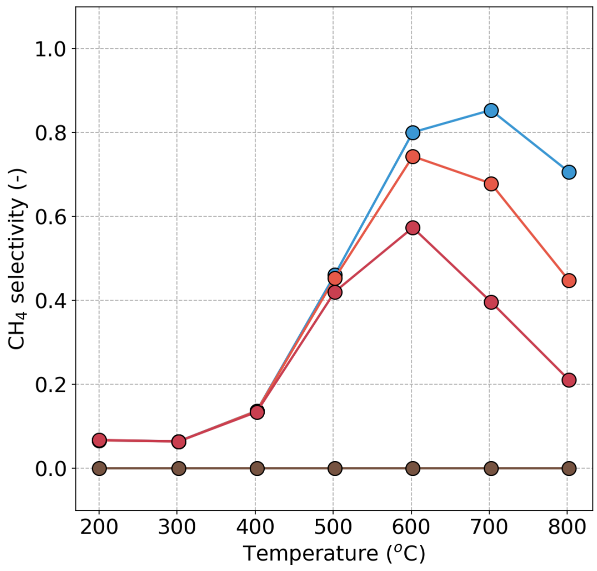

Figure S11. Methane selectivity as a function of the temperature. Blue: E_{act}(CO diss) = 30 kJ/mol; light red: E_{act}(CO diss) = 40 kJ/mol, dark red: E_{act}(CO diss) = 50 kJ/mol, brown: E_{act}(CO diss) = 60 kJ/mol.

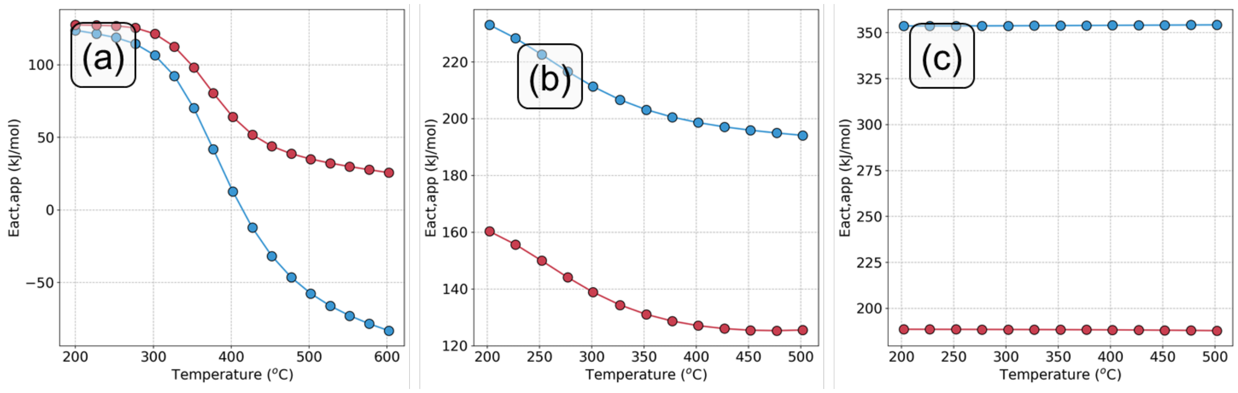

Figure S12. Apparent activation energy with CH_3OH (blue) or CO (red) as key component for (a) Ni_8-cluster, (b) Ni_1-adsorbed and (c) Ni_1-doped.

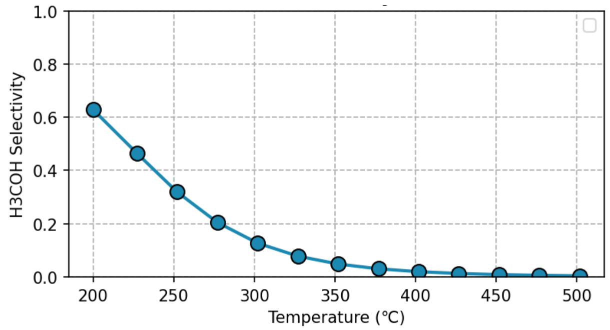

Figure S13. Methanol selectivity at lower H_2 partial pressure (H_2:CO_2 = 1:5).

S12. Comparison with Ni_6-cluster model

Table S12. Forward and backwards activation energies (in kJ/mol) for key elementary reaction steps on Ni_8- and Ni_6-In_2O_3 models.

	Ni_8		Ni_6	
**Elementary Reaction Step**	E_a	E_b	E_a	E_b
$CO^* + * \rightleftarrows C^* + O^*$	231	135	270	154
$HCO_2^* + H^* \rightleftarrows H_2CO_2^* + *$	177	40	133	76
$CO_2^* + * \rightleftarrows CO^* + O^*$	114	144	98	121

Table S13. Initial, transition and final states the calculations on Ni_6-In_2O_3 as reported in Table S8.

$CO^* + * \rightleftarrows C^* + O^*$			$HCO_2^* + H^* \rightleftarrows H_2CO_2^* + *$		
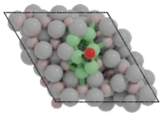	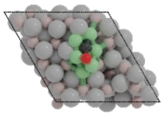	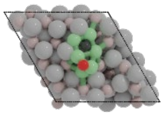	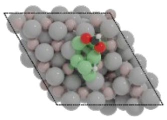	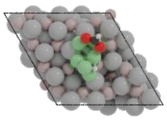	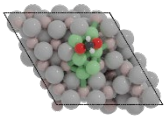
$CO_2^* + * \rightleftarrows CO^* + O^*$					
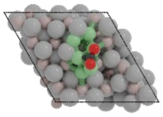	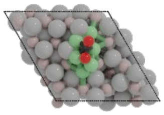	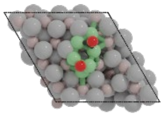			

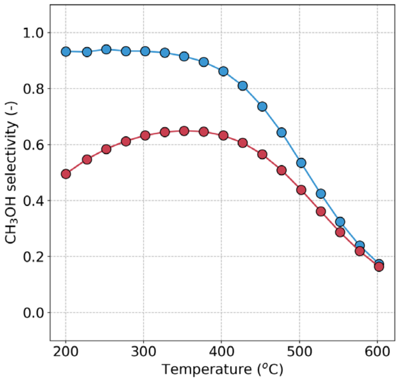

Figure S14. Methanol selectivity for different Ni clusters as a function of temperature. Red: Ni_6-In_2O_3; blue: Ni_8-In_2O_3.

Section S13 . Coordinates of the models

S13.1. **Ni_8-cluster model.**

```
O In Ni
  1.00000000000000
   14.5657999999999994    0.0000000000000000    0.0000000000000000
   -7.2828999999999997   12.6143500000000000    0.0000000000000000
    0.0000000000000000    0.0000000000000000   26.0146000000000015
  O    In   Ni
   96    64     8
Selective dynamics
Direct
  0.1712503710388233  0.5891963298506226  0.7118193153629377   T   T   T
  0.4164360654394486  0.8252367326184782  0.7117962634082531   T   T   T
  0.3980044793319593  0.5801049013246526  0.7128734566953554   T   T   T
  0.6654050227711181  0.8178736193184903  0.7092269519516137   T   T   T
  0.1840719843285612  0.8446239269347586  0.7009090743479263   T   T   T
  0.1509970091277424  0.3354887142403911  0.6998330026310882   T   T   T
  0.9108115485247552  0.8404382417012499  0.6973042231097395   T   T   T
  0.9326638223103103  0.0929177533943317  0.6974291256609177   T   T   T
  0.1592097201033837  0.0666295648052539  0.6978839283958038   T   T   T
  0.9347713176457901  0.3277970201978988  0.6945594501850607   T   T   T
  0.6768744295315727  0.6140111091238083  0.6865856993332002   T   T   T
  0.3953594762268793  0.0608535528100731  0.6934591600895175   T   T   T
  0.8012716628189764  0.5088169646444598  0.6842272698332010   T   T   T
  0.4988120236816869  0.2695642348341423  0.6832975867297293   T   T   T
  0.7227900662010498  0.2257075287842127  0.6619037225479403   T   T   T
  0.9995656962949511  0.7264879253876231  0.6473903758557924   T   T   T
  0.7288114975584037  0.0003371769382809  0.6523548487652775   T   T   T
  0.2771126890659588  0.2678238886524795  0.6503681330045765   T   T   T
```

0.4696255451372835	0.4853919566673852	0.6408806329286421	T	T	T
0.5193839125121471	0.9913992481665872	0.6403457566182666	T	T	T
0.0090065628775804	0.5253045243762884	0.6398586134091744	T	T	T
0.2576981738904109	0.5151246307508615	0.6312821782746170	T	T	T
0.4897452271139784	0.7435476869247495	0.6321843090528532	T	T	T
0.2573096114323263	0.7437837610850835	0.6303213019036278	T	T	T
0.8443231267274101	0.9162841079459199	0.5989534896374380	T	T	T
0.0828889656902874	0.9279581032164117	0.5979007432871697	T	T	T
0.0724677199428712	0.1536240683013972	0.5984983166718152	T	T	T
0.8568844982107890	0.1834281193150886	0.5880575968314190	T	T	T
0.8134741076813583	0.6683394112533776	0.5879222630978944	T	T	T
0.3297148133095433	0.1427582951384707	0.5889602867352011	T	T	T
0.3337113713626678	0.9395829786157535	0.5810105878916545	T	T	T
0.6063113665274074	0.6650048863687843	0.5806358157862803	T	T	T
0.0593525702670092	0.3924772857530172	0.5812734718023904	T	T	T
0.6057754677149276	0.4262307398781786	0.5864008430423340	T	T	T
0.5657758140438389	0.1758551423208274	0.5818076830211965	T	T	T
0.8209262850670953	0.3947909684891329	0.5819549028546831	T	T	T
0.3963724870042782	0.5655157246074864	0.5376327810117303	T	T	T
0.1686725682751486	0.6029745311802159	0.5373028841486089	T	T	T
0.4345752213797027	0.8302496413071196	0.5377270752967576	T	T	T
0.9375207530525032	0.6021672965716149	0.5340847549917456	T	T	T
0.3979128873769682	0.3352924126424028	0.5353526268936903	T	T	T
0.6640537541631346	0.0620098322189051	0.5356270457517311	T	T	T
0.6766573489635306	0.8599629548275018	0.5278481553317178	T	T	T
0.1846557193312961	0.3225709407599950	0.5278337140252347	T	T	T
0.1395649715849814	0.8156682414107195	0.5271113275539889	T	T	T
0.9253360816790988	0.8431520146532634	0.5180983396108161	T	T	T
0.1569502037106574	0.0819176241906092	0.5180093935886916	T	T	T

0.9177768051123323	0.0725746022484586	0.5180526192710531	T	T	T
0.7474300000000014	0.2590300000000028	0.4831199999999995	F	F	F
0.5116000000000014	0.2510499999999993	0.4831199999999995	F	F	F
0.7394499999999979	0.4868799999999993	0.4831199999999995	F	F	F
0.4821200000000019	0.0083800000000025	0.4730699999999999	F	F	F
0.5262600000000006	0.5163599999999988	0.4730699999999999	F	F	F
0.9900999999999982	0.4722200000000001	0.4730699999999999	F	F	F
0.2715400000000017	0.9989300000000014	0.4665899999999965	F	F	F
0.7273899999999998	0.7269399999999990	0.4665899999999965	F	F	F
0.9995499999999993	0.2710900000000009	0.4665899999999965	F	F	F
0.4968900000000005	0.7296100000000010	0.4629400000000032	F	F	F
0.2327200000000005	0.5015900000000002	0.4629400000000032	F	F	F
0.2688800000000029	0.7657700000000034	0.4629400000000032	F	F	F
0.8354300000000023	0.9350400000000008	0.4227699999999999	F	F	F
0.0996099999999984	0.1630499999999984	0.4227699999999999	F	F	F
0.0634399999999999	0.8988799999999983	0.4227699999999999	F	F	F
0.0607800000000012	0.6657099999999971	0.4191199999999995	F	F	F
0.6049300000000031	0.9376999999999995	0.4191199999999995	F	F	F
0.3327699999999965	0.3935600000000008	0.4191199999999995	F	F	F
0.8502000000000010	0.6562600000000032	0.4126400000000032	F	F	F
0.8060600000000022	0.1482799999999997	0.4126400000000032	F	F	F
0.3422199999999975	0.1924199999999985	0.4126400000000032	F	F	F
0.5928699999999978	0.1777599999999993	0.4025900000000036	F	F	F
0.5848900000000015	0.4056100000000029	0.4025900000000036	F	F	F
0.8207200000000014	0.4135899999999992	0.4025900000000036	F	F	F
0.4140999999999977	0.5923700000000025	0.3688300000000027	F	F	F
0.1782699999999977	0.5843899999999991	0.3688300000000027	F	F	F
0.4061200000000014	0.8202100000000030	0.3688300000000027	F	F	F
0.1487899999999982	0.3417099999999991	0.3587800000000030	F	F	F

0.1929299999999969	0.8496999999999986	0.3587800000000030	F	F	F
0.6567700000000016	0.8055499999999967	0.3587800000000030	F	F	F
0.9382000000000019	0.3322700000000012	0.3522999999999996	F	F	F
0.3940600000000032	0.0602799999999988	0.3522999999999996	F	F	F
0.6662200000000027	0.6044199999999975	0.3522999999999996	F	F	F
0.1635599999999968	0.0629399999999976	0.3486499999999992	F	F	F
0.8993800000000007	0.8349299999999999	0.3486499999999992	F	F	F
0.9355400000000031	0.0991000000000000	0.3486499999999992	F	F	F
0.5020999999999987	0.2683699999999973	0.3084800000000030	F	F	F
0.7662699999999987	0.4963800000000020	0.3084800000000030	F	F	F
0.7301100000000034	0.2322100000000020	0.3084800000000030	F	F	F
0.7274499999999975	0.9990400000000008	0.3048300000000026	F	F	F
0.2715900000000033	0.2710300000000032	0.3048300000000026	F	F	F
0.9994399999999999	0.7268899999999974	0.3048300000000026	F	F	F
0.4727299999999985	0.4816100000000034	0.2983499999999992	F	F	F
0.0088900000000010	0.5257599999999982	0.2983499999999992	F	F	F
0.5168699999999973	0.9896000000000029	0.2983499999999992	F	F	F
0.2515599999999978	0.7389399999999995	0.2882999999999996	F	F	F
0.4873800000000017	0.7469200000000029	0.2882999999999996	F	F	F
0.2595400000000012	0.5110999999999990	0.2882999999999996	F	F	F
0.3277687632153672	0.6681699044892042	0.6777498861597167	T	T	T
0.8291511613159983	0.9276239537817474	0.6807566115171895	T	T	T
0.0785729727412772	0.8993216931715793	0.6797602142942409	T	T	T
0.1006379477244366	0.1748989162752892	0.6802624615986801	T	T	T
0.5677886409256274	0.8894180282244335	0.6765288468357795	T	T	T
0.3121644911595993	0.4246008281979456	0.6746282397234182	T	T	T
0.1117328522329046	0.6801237810013916	0.6750674198049609	T	T	T
0.8432839624608874	0.1678610414254678	0.6712330816808461	T	T	T
0.8418095466333071	0.6786833669300947	0.6703899169030905	T	T	T

0.3221421979156105	0.1501969257108371	0.6729755799633423	T	T	T
0.0949264218726782	0.4391451775914348	0.6690458131586681	T	T	T
0.5586543130467945	0.6566546034426963	0.6641076490579820	T	T	T
0.3427567045768711	0.9027182977012700	0.6688664371384121	T	T	T
0.5833800789727803	0.4426073702769671	0.6672140586542028	T	T	T
0.5537467258919975	0.1491381389635293	0.6647934442334460	T	T	T
0.8511282475041853	0.3991617589248690	0.6646284433420533	T	T	T
0.7707146345879963	0.0099565968311452	0.5659674070877756	T	T	T
0.9905332933808211	0.7604033474619314	0.5644281828899267	T	T	T
0.2388312884875887	0.2274170202015497	0.5663620572968995	T	T	T
0.7352416704734850	0.2258757604466410	0.5654975250703345	T	T	T
0.7721695061424256	0.5097967138887967	0.5623310521780454	T	T	T
0.4874933691788428	0.2606216281813829	0.5621260738993570	T	T	T
0.0002958192744913	0.9996645552386170	0.5577956345820780	T	T	T
0.4995650074012019	0.5017495288041118	0.5565623630977896	T	T	T
0.4966844897229379	0.9980929355378407	0.5572517403384580	T	T	T
0.0012363411811052	0.4984692811165417	0.5571555032993345	T	T	T
0.2257737073730510	0.4889106391102231	0.5495953427605471	T	T	T
0.5101584514172259	0.7372994550054344	0.5488001659630157	T	T	T
0.2626817713012315	0.7728870390648339	0.5490186162213888	T	T	T
0.0113626749943734	0.2391366592914110	0.5495516586520210	T	T	T
0.7606830909251343	0.7710984301201123	0.5493548794438325	T	T	T
0.2290866169280434	0.9882945415649118	0.5494683424693960	T	T	T
0.9050099999999972	0.5600299999999976	0.4504999999999981	F	F	F
0.4050099999999972	0.5600299999999976	0.4504999999999981	F	F	F
0.1550099999999972	0.5934700000000035	0.4504999999999981	F	F	F
0.4384599999999992	0.8434700000000035	0.4504999999999981	F	F	F
0.6550099999999972	0.0934700000000035	0.4504999999999981	F	F	F
0.4384599999999992	0.3434700000000035	0.4504999999999981	F	F	F

0.6661599999999979	0.3323200000000028	0.4428500000000000	F	F	F
0.1661599999999979	0.8323200000000028	0.4428500000000000	F	F	F
0.6661599999999979	0.8323200000000028	0.4428500000000000	F	F	F
0.1661599999999979	0.3323200000000028	0.4428500000000000	F	F	F
0.4273099999999985	0.1046199999999970	0.4352099999999979	F	F	F
0.9273099999999985	0.1046199999999970	0.4352099999999979	F	F	F
0.1773099999999985	0.0711700000000022	0.4352099999999979	F	F	F
0.6773099999999985	0.5711700000000022	0.4352099999999979	F	F	F
0.8938599999999965	0.3211700000000022	0.4352099999999979	F	F	F
0.8938599999999965	0.8211700000000022	0.4352099999999979	F	F	F
0.5716800000000006	0.8933600000000013	0.3362100000000012	F	F	F
0.0716800000000006	0.8933600000000013	0.3362100000000012	F	F	F
0.8216800000000006	0.9268000000000001	0.3362100000000012	F	F	F
0.3216800000000006	0.4268000000000001	0.3362100000000012	F	F	F
0.1051199999999994	0.6768000000000001	0.3362100000000012	F	F	F
0.1051199999999994	0.1768000000000001	0.3362100000000012	F	F	F
0.8328300000000013	0.1656499999999994	0.3285600000000031	F	F	F
0.3328300000000013	0.1656499999999994	0.3285600000000031	F	F	F
0.8328300000000013	0.6656499999999994	0.3285600000000031	F	F	F
0.3328300000000013	0.6656499999999994	0.3285600000000031	F	F	F
0.5939800000000020	0.4379500000000007	0.3209200000000010	F	F	F
0.8439800000000020	0.4045100000000019	0.3209200000000010	F	F	F
0.5605300000000000	0.1545100000000019	0.3209200000000010	F	F	F
0.0939800000000020	0.4379500000000007	0.3209200000000010	F	F	F
0.3439800000000020	0.9045100000000019	0.3209200000000010	F	F	F
0.5605300000000000	0.6545100000000019	0.3209200000000010	F	F	F
0.4935309162814647	0.4500411816449059	0.7869199479711497	T	T	T
0.5416505052611171	0.3329612559239834	0.7501886142852996	T	T	T
0.4668252834661430	0.5896920339378795	0.7727390815576243	T	T	T

0.6350069711101938 0.7284637674699610 0.7675667769498915 T T T

0.7170611531867124 0.4610702622753848 0.7421021184087948 T T T

0.6566573032052645 0.5809459054036736 0.7609870993393244 T T T

0.6381067663744417 0.4491803930385601 0.8207761818198733 T T T

0.5889640194111010 0.6053550426826746 0.8366001037719357 T T T

S13.2. **Ni_6-cluster model.**

O In Ni

1.00000000000000

14.5657999999999994 0.0000000000000000 0.0000000000000000

-7.2828999999999997 12.6143500000000000 0.0000000000000000

0.0000000000000000 0.0000000000000000 26.0146000000000015

O In Ni

96 64 6

Selective dynamics

Direct

0.1662886122775191 0.5840417349922618 0.7112284728357423 T T T

0.4056794455942949 0.8179797651396683 0.7116219249666855 T T T

0.3680763976813211 0.5610969664771943 0.7365793334177368 T T T

0.6107553769544438 0.8144209509682199 0.7428736944018581 T T T

0.1851343415704108 0.8430935934686445 0.7014983062681921 T T T

0.1453136035052367 0.3305083605313541 0.6990611361862449 T T T

0.9252697192682433 0.8467929352835029 0.6945642332362496 T T T

0.9314979171094890 0.0910456087632667 0.6969100328188134 T T T

0.1628413992389142 0.0697025375394347 0.6981974873050945 T T T

0.9271389343001459 0.3252711562754484 0.6945356887105589 T T T

0.7388434850104488 0.6575401970503165 0.7291840840336761 T T T

0.3944290776073132 0.0628164132684645 0.6927796627941092 T T T

0.7711576353654621 0.4988605731662972 0.6593527279841638 T T T

0.4974169885653339	0.2783643873393729	0.6872333288600743	T	T	T
0.7182199551702197	0.2239089292238532	0.6640611118302644	T	T	T
0.0017135443860923	0.7239865881003666	0.6459743098150170	T	T	T
0.7241214430635310	0.9894864576386411	0.6568638209078657	T	T	T
0.2747825658873282	0.2670031577557879	0.6478775511708434	T	T	T
0.4711528112296790	0.4865463506539456	0.6397259991260782	T	T	T
0.5170723047535404	0.9894405970859381	0.6419631970501527	T	T	T
0.0023696037684076	0.5217193020938211	0.6409897867205032	T	T	T
0.2633448107088441	0.5138658925210748	0.6341521739917013	T	T	T
0.4872025979368431	0.7410705834967430	0.6323621441401244	T	T	T
0.2536229011374829	0.7406962397372538	0.6295801331732136	T	T	T
0.8454672927759290	0.9176248419348170	0.5988584268051724	T	T	T
0.0851577725020292	0.9281279792780595	0.5972555022256785	T	T	T
0.0709481819434714	0.1522701405734068	0.5981440880547240	T	T	T
0.8537500679862973	0.1784787273019930	0.5901546185833340	T	T	T
0.8125074515325679	0.6724727496358014	0.5894215104222553	T	T	T
0.3298601270988897	0.1424756650912045	0.5887469109802552	T	T	T
0.3330777877566717	0.9388143554480521	0.5814375216142338	T	T	T
0.6070260620382086	0.6631496090655133	0.5781480094560862	T	T	T
0.0600140753560297	0.3916741274059703	0.5832017779616834	T	T	T
0.6003523475235042	0.4240169979743413	0.5825118996768595	T	T	T
0.5647575964049381	0.1750216747829043	0.5829046405711296	T	T	T
0.8225292991607134	0.3894498000458100	0.5791469584810685	T	T	T
0.3956008304971945	0.5650525243377089	0.5375532549617219	T	T	T
0.1681357190586183	0.6009258890467066	0.5379579728774004	T	T	T
0.4335970321295974	0.8298820959260169	0.5379906320572800	T	T	T
0.9359528940376123	0.6013568651204650	0.5347739979244679	T	T	T
0.3940980469056328	0.3321531515115481	0.5337158773516464	T	T	T
0.6624446767702636	0.0594421193494981	0.5364513225129315	T	T	T

0.6755459714695675	0.8585416495061153	0.5284009475774951	T	T	T
0.1829272151789212	0.3224832942459557	0.5279732308547054	T	T	T
0.1397509364953180	0.8150378355665850	0.5267306171580515	T	T	T
0.9263863294064123	0.8435650407295867	0.5177614588421235	T	T	T
0.1563921273978721	0.0815682050535216	0.5176881453183881	T	T	T
0.9164998860945275	0.0720418840491064	0.5180414684856558	T	T	T
0.7474300000000014	0.2590300000000028	0.4831199999999995	F	F	F
0.5116000000000014	0.2510499999999993	0.4831199999999995	F	F	F
0.7394499999999979	0.4868799999999993	0.4831199999999995	F	F	F
0.4821200000000019	0.0083800000000025	0.4730699999999999	F	F	F
0.5262600000000006	0.5163599999999988	0.4730699999999999	F	F	F
0.9900999999999982	0.4722200000000001	0.4730699999999999	F	F	F
0.2715400000000017	0.9989300000000014	0.4665899999999965	F	F	F
0.7273899999999998	0.7269399999999990	0.4665899999999965	F	F	F
0.9995499999999993	0.2710900000000009	0.4665899999999965	F	F	F
0.4968900000000005	0.7296100000000010	0.4629400000000032	F	F	F
0.2327200000000005	0.5015900000000002	0.4629400000000032	F	F	F
0.2688800000000029	0.7657700000000034	0.4629400000000032	F	F	F
0.8354300000000023	0.9350400000000008	0.4227699999999999	F	F	F
0.0996099999999984	0.1630499999999984	0.4227699999999999	F	F	F
0.0634399999999999	0.8988799999999983	0.4227699999999999	F	F	F
0.0607800000000012	0.6657099999999971	0.4191199999999995	F	F	F
0.6049300000000031	0.9376999999999995	0.4191199999999995	F	F	F
0.3327699999999965	0.3935600000000008	0.4191199999999995	F	F	F
0.8502000000000010	0.6562600000000032	0.4126400000000032	F	F	F
0.8060600000000022	0.1482799999999997	0.4126400000000032	F	F	F
0.3422199999999975	0.1924199999999985	0.4126400000000032	F	F	F
0.5928699999999978	0.1777599999999993	0.4025900000000036	F	F	F
0.5848900000000015	0.4056100000000029	0.4025900000000036	F	F	F

0.8207200000000014	0.4135899999999992	0.4025900000000036	F	F	F
0.4140999999999977	0.5923700000000025	0.3688300000000027	F	F	F
0.1782699999999977	0.5843899999999991	0.3688300000000027	F	F	F
0.4061200000000014	0.8202100000000030	0.3688300000000027	F	F	F
0.1487899999999982	0.3417099999999991	0.3587800000000030	F	F	F
0.1929299999999969	0.8496999999999986	0.3587800000000030	F	F	F
0.6567700000000016	0.8055499999999967	0.3587800000000030	F	F	F
0.9382000000000019	0.3322700000000012	0.3522999999999996	F	F	F
0.3940600000000032	0.0602799999999988	0.3522999999999996	F	F	F
0.6662200000000027	0.6044199999999975	0.3522999999999996	F	F	F
0.1635599999999968	0.0629399999999976	0.3486499999999992	F	F	F
0.8993800000000007	0.8349299999999999	0.3486499999999992	F	F	F
0.9355400000000031	0.0991000000000000	0.3486499999999992	F	F	F
0.5020999999999987	0.2683699999999973	0.3084800000000030	F	F	F
0.7662699999999987	0.4963800000000020	0.3084800000000030	F	F	F
0.7301100000000034	0.2322100000000020	0.3084800000000030	F	F	F
0.7274499999999975	0.9990400000000008	0.3048300000000026	F	F	F
0.2715900000000033	0.2710300000000032	0.3048300000000026	F	F	F
0.9994399999999999	0.7268899999999974	0.3048300000000026	F	F	F
0.4727299999999985	0.4816100000000034	0.2983499999999992	F	F	F
0.0088900000000010	0.5257599999999982	0.2983499999999992	F	F	F
0.5168699999999973	0.9896000000000029	0.2983499999999992	F	F	F
0.2515599999999978	0.7389399999999995	0.2882999999999996	F	F	F
0.4873800000000017	0.7469200000000029	0.2882999999999996	F	F	F
0.2595400000000012	0.5110999999999990	0.2882999999999996	F	F	F
0.3218101376621263	0.6631532771695205	0.6769685406956256	T	T	T
0.8345338104842099	0.9252089449254726	0.6818070789136996	T	T	T
0.0867444580384884	0.9033160997238454	0.6792115821692661	T	T	T
0.0983426991465208	0.1719636381045362	0.6797965927162792	T	T	T

0.5610259931848418	0.8864480435136954	0.6801801953942714	T	T	T
0.3106325516963437	0.4177882607324293	0.6779328614888342	T	T	T
0.1102418417388265	0.6776187621825258	0.6749115963576457	T	T	T
0.8385381334969892	0.1633496568349599	0.6733029664063110	T	T	T
0.8478260922942553	0.6801557011073527	0.6706240128465026	T	T	T
0.3243734833172667	0.1552422016497701	0.6726130103873729	T	T	T
0.0891252373000384	0.4335431606165230	0.6702820133246163	T	T	T
0.5592169895958889	0.6520562568352091	0.6569007220078689	T	T	T
0.3407167185143294	0.9020697447257646	0.6682928328065927	T	T	T
0.6006140903778245	0.4441416998499363	0.6644530657118963	T	T	T
0.5514257664004418	0.1488309940852588	0.6659280043771574	T	T	T
0.8427820814647973	0.3965757315896710	0.6624339773131922	T	T	T
0.7694271838623479	0.0070950471450715	0.5675454254306942	T	T	T
0.9909276486403797	0.7602704933277593	0.5640816587194735	T	T	T
0.2370640195868816	0.2263421951525828	0.5652839768270468	T	T	T
0.7339244025184612	0.2225082800579372	0.5648862713138953	T	T	T
0.7726280066020905	0.5091789962991058	0.5651015614977695	T	T	T
0.4855984489197169	0.2586157989001876	0.5613939356338378	T	T	T
0.0002552920427874	0.9993093967698670	0.5576423794657259	T	T	T
0.4965898236785549	0.4974183273898589	0.5555649241276157	T	T	T
0.4958973124059298	0.9975444585922730	0.5578560267164860	T	T	T
0.9999571810434276	0.4967675786971668	0.5574730409139832	T	T	T
0.2255845464763411	0.4876736234815056	0.5507820436700968	T	T	T
0.5087836293564365	0.7371256256297087	0.5481053940863916	T	T	T
0.2616277976031398	0.7717905882737526	0.5486546026001378	T	T	T
0.0093927433562371	0.2375676185568523	0.5500339789183499	T	T	T
0.7625922540349208	0.7725504409430641	0.5487994561551730	T	T	T
0.2294309542426092	0.9882675066557525	0.5491221864937046	T	T	T
0.9050099999999972	0.5600299999999976	0.4504999999999981	F	F	F

0.4050099999999972 0.5600299999999976 0.4504999999999981 F F F

0.1550099999999972 0.5934700000000035 0.4504999999999981 F F F

0.4384599999999992 0.8434700000000035 0.4504999999999981 F F F

0.6550099999999972 0.0934700000000035 0.4504999999999981 F F F

0.4384599999999992 0.3434700000000035 0.4504999999999981 F F F

0.6661599999999979 0.3323200000000028 0.4428500000000000 F F F

0.1661599999999979 0.8323200000000028 0.4428500000000000 F F F

0.6661599999999979 0.8323200000000028 0.4428500000000000 F F F

0.1661599999999979 0.3323200000000028 0.4428500000000000 F F F

0.4273099999999985 0.1046199999999970 0.4352099999999979 F F F

0.9273099999999985 0.1046199999999970 0.4352099999999979 F F F

0.1773099999999985 0.0711700000000022 0.4352099999999979 F F F

0.6773099999999985 0.5711700000000022 0.4352099999999979 F F F

0.8938599999999965 0.3211700000000022 0.4352099999999979 F F F

0.8938599999999965 0.8211700000000022 0.4352099999999979 F F F

0.5716800000000006 0.8933600000000013 0.3362100000000012 F F F

0.0716800000000006 0.8933600000000013 0.3362100000000012 F F F

0.8216800000000006 0.9268000000000001 0.3362100000000012 F F F

0.3216800000000006 0.4268000000000001 0.3362100000000012 F F F

0.1051199999999994 0.6768000000000001 0.3362100000000012 F F F

0.1051199999999994 0.1768000000000001 0.3362100000000012 F F F

0.8328300000000013 0.1656499999999994 0.3285600000000031 F F F

0.3328300000000013 0.1656499999999994 0.3285600000000031 F F F

0.8328300000000013 0.6656499999999994 0.3285600000000031 F F F

0.3328300000000013 0.6656499999999994 0.3285600000000031 F F F

0.5939800000000020 0.4379500000000007 0.3209200000000010 F F F

0.8439800000000020 0.4045100000000019 0.3209200000000010 F F F

0.5605300000000000 0.1545100000000019 0.3209200000000010 F F F

0.0939800000000020 0.4379500000000007 0.3209200000000010 F F F

0.3439800000000020 0.9045100000000019 0.3209200000000010 F F F

0.5605300000000000 0.6545100000000019 0.3209200000000010 F F F

0.4846519117959645 0.3599094764857753 0.7428619230793743 T T T

0.4252051503897434 0.4852889688707762 0.7645503168143648 T T T

0.4971857888263216 0.6786042756385058 0.7479562114069935 T T T

0.6067552545493200 0.5528036328944737 0.7479248520958350 T T T

0.6848774592536699 0.7480219946895161 0.7301175219925256 T T T

0.5226925222886578 0.4471812777942219 0.8183241241468764 T T T

S13.3. **Ni_1-adsorbed model.**

1.0

14.56580 0.00000 0.00000

-7.28290 12.61435 0.00000

0.00000 0.00000 26.01460

O In Ni

96 64 1

Selective Dynamics

Direct

0.17158 0.58877 0.71137 T T T

0.41693 0.82604 0.71110 T T T

0.40899 0.58094 0.71140 T T T

0.66198 0.81363 0.69852 T T T

0.18427 0.84639 0.69871 T T T

0.15185 0.33607 0.69881 T T T

0.90610 0.83850 0.69600 T T T

0.93228 0.09179 0.69552 T T T

0.15957 0.06580 0.69579 T T T

0.93080 0.32894 0.69276 T T T

0.66843 0.59905 0.69325 T T T

0.39835 0.06708 0.69270 T T T

0.77053	0.49197	0.65893	T	T	T
0.50571	0.27630	0.65847	T	T	T
0.72137	0.22694	0.65883	T	T	T
-0.00854	0.72116	0.65123	T	T	T
0.72936	0.00592	0.65055	T	T	T
0.27676	0.26858	0.65090	T	T	T
0.47248	0.47923	0.64020	T	T	T
0.51852	0.99084	0.63991	T	T	T
0.00653	0.52545	0.64012	T	T	T
0.25522	0.51322	0.63062	T	T	T
0.48448	0.73953	0.63051	T	T	T
0.25772	0.74258	0.63057	T	T	T
0.84486	0.91808	0.59761	T	T	T
0.07987	0.92461	0.59782	T	T	T
0.07349	0.15342	0.59766	T	T	T
0.85818	0.18342	0.58670	T	T	T
0.81454	0.67266	0.58709	T	T	T
0.32517	0.13984	0.58669	T	T	T
0.33262	0.93858	0.58080	T	T	T
0.60604	0.66504	0.58098	T	T	T
0.05931	0.39215	0.58089	T	T	T
0.60634	0.43280	0.57700	T	T	T
0.56520	0.17134	0.57689	T	T	T
0.82623	0.39151	0.57711	T	T	T
0.39565	0.56378	0.53667	T	T	T
0.16796	0.60240	0.53667	T	T	T
0.43422	0.82981	0.53660	T	T	T
0.93788	0.60262	0.53442	T	T	T
0.39553	0.33345	0.53434	T	T	T

0.66475	0.06012	0.53418	T	T	T
0.67645	0.86010	0.52701	T	T	T
0.18393	0.32179	0.52692	T	T	T
0.13766	0.81428	0.52699	T	T	T
0.92523	0.84335	0.51715	T	T	T
0.15444	0.07968	0.51710	T	T	T
0.91848	0.07280	0.51702	T	T	T
0.74743	0.25903	0.48312	F	F	F
0.51160	0.25105	0.48312	F	F	F
0.73945	0.48688	0.48312	F	F	F
0.48212	0.00838	0.47307	F	F	F
0.52626	0.51636	0.47307	F	F	F
0.99010	0.47222	0.47307	F	F	F
0.27154	0.99893	0.46659	F	F	F
0.72739	0.72694	0.46659	F	F	F
0.99955	0.27109	0.46659	F	F	F
0.49689	0.72961	0.46294	F	F	F
0.23272	0.50159	0.46294	F	F	F
0.26888	0.76577	0.46294	F	F	F
0.83543	0.93504	0.42277	F	F	F
0.09961	0.16305	0.42277	F	F	F
0.06344	0.89888	0.42277	F	F	F
0.06078	0.66571	0.41912	F	F	F
0.60493	0.93770	0.41912	F	F	F
0.33277	0.39356	0.41912	F	F	F
0.85020	0.65626	0.41264	F	F	F
0.80606	0.14828	0.41264	F	F	F
0.34222	0.19242	0.41264	F	F	F
0.59287	0.17776	0.40259	F	F	F

0.58489	0.40561	0.40259	F	F	F
0.82072	0.41359	0.40259	F	F	F
0.41410	0.59237	0.36883	F	F	F
0.17827	0.58439	0.36883	F	F	F
0.40612	0.82021	0.36883	F	F	F
0.14879	0.34171	0.35878	F	F	F
0.19293	0.84970	0.35878	F	F	F
0.65677	0.80555	0.35878	F	F	F
0.93820	0.33227	0.35230	F	F	F
0.39406	0.06028	0.35230	F	F	F
0.66622	0.60442	0.35230	F	F	F
0.16356	0.06294	0.34865	F	F	F
0.89938	0.83493	0.34865	F	F	F
0.93554	0.09910	0.34865	F	F	F
0.50210	0.26837	0.30848	F	F	F
0.76627	0.49638	0.30848	F	F	F
0.73011	0.23221	0.30848	F	F	F
0.72745	0.99904	0.30483	F	F	F
0.27159	0.27103	0.30483	F	F	F
0.99944	0.72689	0.30483	F	F	F
0.47273	0.48161	0.29835	F	F	F
0.00889	0.52576	0.29835	F	F	F
0.51687	0.98960	0.29835	F	F	F
0.25156	0.73894	0.28830	F	F	F
0.48738	0.74692	0.28830	F	F	F
0.25954	0.51110	0.28830	F	F	F
0.33238	0.66513	0.67971	T	T	T
0.82288	0.92420	0.67965	T	T	T
0.07361	0.89653	0.67995	T	T	T

0.10157	0.17540	0.67975	T	T	T
0.56754	0.88465	0.67430	T	T	T
0.31728	0.43024	0.67463	T	T	T
0.11276	0.68064	0.67467	T	T	T
0.84419	0.16869	0.66986	T	T	T
0.82873	0.67315	0.67035	T	T	T
0.32456	0.15352	0.66988	T	T	T
0.09431	0.43900	0.66896	T	T	T
0.55897	0.65316	0.66916	T	T	T
0.34449	0.90348	0.66882	T	T	T
0.59851	0.44437	0.66050	T	T	T
0.55329	0.15167	0.66035	T	T	T
0.84591	0.39915	0.66058	T	T	T
0.77108	0.00982	0.56560	T	T	T
0.98809	0.75914	0.56600	T	T	T
0.23888	0.22714	0.56574	T	T	T
0.73506	0.22343	0.56452	T	T	T
0.77449	0.50958	0.56462	T	T	T
0.48851	0.26292	0.56457	T	T	T
0.99940	0.99864	0.55730	T	T	T
0.50057	0.50158	0.55711	T	T	T
0.49632	0.99682	0.55696	T	T	T
0.00091	0.49736	0.55712	T	T	T
0.22597	0.48866	0.54983	T	T	T
0.50959	0.73539	0.54987	T	T	T
0.26243	0.77202	0.54986	T	T	T
0.01199	0.23885	0.54957	T	T	T
0.75923	0.77091	0.54965	T	T	T
0.22682	0.98612	0.54958	T	T	T

0.90501	0.56003	0.45050	F	F	F
0.40501	0.56003	0.45050	F	F	F
0.15501	0.59347	0.45050	F	F	F
0.43846	0.84347	0.45050	F	F	F
0.65501	0.09347	0.45050	F	F	F
0.43846	0.34347	0.45050	F	F	F
0.66616	0.33232	0.44285	F	F	F
0.16616	0.83232	0.44285	F	F	F
0.66616	0.83232	0.44285	F	F	F
0.16616	0.33232	0.44285	F	F	F
0.42731	0.10462	0.43521	F	F	F
0.92731	0.10462	0.43521	F	F	F
0.17731	0.07117	0.43521	F	F	F
0.67731	0.57117	0.43521	F	F	F
0.89386	0.32117	0.43521	F	F	F
0.89386	0.82117	0.43521	F	F	F
0.57168	0.89336	0.33621	F	F	F
0.07168	0.89336	0.33621	F	F	F
0.82168	0.92680	0.33621	F	F	F
0.32168	0.42680	0.33621	F	F	F
0.10512	0.67680	0.33621	F	F	F
0.10512	0.17680	0.33621	F	F	F
0.83283	0.16565	0.32856	F	F	F
0.33283	0.16565	0.32856	F	F	F
0.83283	0.66565	0.32856	F	F	F
0.33283	0.66565	0.32856	F	F	F
0.59398	0.43795	0.32092	F	F	F
0.84398	0.40451	0.32092	F	F	F
0.56053	0.15451	0.32092	F	F	F

0.09398 0.43795 0.32092 F F F

0.34398 0.90451 0.32092 F F F

0.56053 0.65451 0.32092 F F F

0.29878 0.54939 0.75478 F F F

S13.4. Ni_1-doped model

VASP POSCAR

1.00000000000000

14.5657999999999994 0.0000000000000000 0.0000000000000000

-7.2828999999999997 12.6143500000000000 0.0000000000000000

0.0000000000000000 0.0000000000000000 26.0146000000000015

O In Ni

96 63 1

Selective dynamics

Direct

0.2028381674127573 0.6012940483953884 0.7058248018793805 T T T

0.4078596543788464 0.8001147292391551 0.7057618734892444 T T T

0.4018195509866302 0.5957311619995309 0.7054938508695656 T T T

0.6637540085293026 0.8112323912329202 0.6954973596538281 T T T

0.1915227878196077 0.8458719401226347 0.6959842012831672 T T T

0.1567851983346031 0.3399142259464867 0.6960752022531480 T T T

0.9102640642035240 0.8386112810487703 0.6954190398159454 T T T

0.9372016650393888 0.0927706520373661 0.6954702927478849 T T T

0.1638712015720714 0.0654496355818068 0.6952148060767485 T T T

0.9367814304910453 0.3312273784564042 0.6923646441623177 T T T

0.6713021549266287 0.5997950572452879 0.6922987467655588 T T T

0.4020540038377902 0.0651455745564959 0.6917926605624771 T T T

0.7751277124936422 0.4928518541023834 0.6596431703097423 T T T

0.5085210640187157 0.2764082367407212 0.6597721785919930 T T T

0.7253935514219023 0.2269987564055498 0.6597065434767302 T T T

0.9978260437753548	0.7219125490823046	0.6506715173640956	T	T	T
0.7325939457038970	0.0051525446135159	0.6510180536618719	T	T	T
0.2799823049142134	0.2701060956629675	0.6505042321055186	T	T	T
0.4740858722131449	0.4814483749471705	0.6422117631027362	T	T	T
0.5208032056972414	0.9867032544237020	0.6420023751266442	T	T	T
0.0152053165801860	0.5282458053718528	0.6423483362077178	T	T	T
0.2645384009423353	0.5286622563660472	0.6297740903206090	T	T	T
0.4740620019595170	0.7306628015341730	0.6298561709185719	T	T	T
0.2724422436688201	0.7378282613111651	0.6298396873568777	T	T	T
0.8474309572878127	0.9177831460666894	0.5971267738718069	T	T	T
0.0828160328815315	0.9250753873328179	0.5971680650258691	T	T	T
0.0757816071995876	0.1537161445404394	0.5973394020905829	T	T	T
0.8615787024526682	0.1848432267975966	0.5870698694674061	T	T	T
0.8162343253679012	0.6726386032438043	0.5874236296155573	T	T	T
0.3282221666813925	0.1391213902367596	0.5867062672235690	T	T	T
0.3339162544029080	0.9347241367090078	0.5817170753793945	T	T	T
0.6055644530785784	0.6660236969770708	0.5812911416510005	T	T	T
0.0659704975908468	0.3950956006451634	0.5817095215386416	T	T	T
0.6079536815658102	0.4330362386191595	0.5781278267862433	T	T	T
0.5674616674096882	0.1702491844666767	0.5782319436609384	T	T	T
0.8308196110688257	0.3927318362726879	0.5782000200008426	T	T	T
0.3967287698971660	0.5625366464813695	0.5367384065036944	T	T	T
0.1691260448089835	0.6032700119513876	0.5368315994643282	T	T	T
0.4372223046902093	0.8306936439456010	0.5366389783628591	T	T	T
0.9396057278687735	0.6021292863517616	0.5350167846512193	T	T	T
0.3977856594536107	0.3340638394394020	0.5352741009653030	T	T	T
0.6665564016565995	0.0603354287833895	0.5348641709691293	T	T	T
0.6768923856817253	0.8596325767329986	0.5276776188265870	T	T	T
0.1861822512259579	0.3229124909598298	0.5271916678921897	T	T	T

0.1403819328116498	0.8136875575698781	0.5275536272788486	T	T	T
0.9267189276375376	0.8427322531974966	0.5171892020419548	T	T	T
0.1567324073619574	0.0803988044233677	0.5173684108315939	T	T	T
0.9200910123223167	0.0730687805405523	0.5172646370050629	T	T	T
0.7474300000000014	0.2590300000000028	0.4831199999999995	F	F	F
0.5116000000000014	0.2510499999999993	0.4831199999999995	F	F	F
0.7394499999999979	0.4868799999999993	0.4831199999999995	F	F	F
0.4821200000000019	0.0083800000000025	0.4730699999999999	F	F	F
0.5262600000000006	0.5163599999999988	0.4730699999999999	F	F	F
0.9900999999999982	0.4722200000000001	0.4730699999999999	F	F	F
0.2715400000000017	0.9989300000000014	0.4665899999999965	F	F	F
0.7273899999999998	0.7269399999999990	0.4665899999999965	F	F	F
0.9995499999999993	0.2710900000000009	0.4665899999999965	F	F	F
0.4968900000000005	0.7296100000000010	0.4629400000000032	F	F	F
0.2327200000000005	0.5015900000000002	0.4629400000000032	F	F	F
0.2688800000000029	0.7657700000000034	0.4629400000000032	F	F	F
0.8354300000000023	0.9350400000000008	0.4227699999999999	F	F	F
0.0996099999999984	0.1630499999999984	0.4227699999999999	F	F	F
0.0634399999999999	0.8988799999999983	0.4227699999999999	F	F	F
0.0607800000000012	0.6657099999999971	0.4191199999999995	F	F	F
0.6049300000000031	0.9376999999999995	0.4191199999999995	F	F	F
0.3327699999999965	0.3935600000000008	0.4191199999999995	F	F	F
0.8502000000000010	0.6562600000000032	0.4126400000000032	F	F	F
0.8060600000000022	0.1482799999999997	0.4126400000000032	F	F	F
0.3422199999999975	0.1924199999999985	0.4126400000000032	F	F	F
0.5928699999999978	0.1777599999999993	0.4025900000000036	F	F	F
0.5848900000000015	0.4056100000000029	0.4025900000000036	F	F	F
0.8207200000000014	0.4135899999999992	0.4025900000000036	F	F	F
0.4140999999999977	0.5923700000000025	0.3688300000000027	F	F	F

0.1782699999999977	0.5843899999999991	0.3688300000000027	F	F	F
0.4061200000000014	0.8202100000000030	0.3688300000000027	F	F	F
0.1487899999999982	0.3417099999999991	0.3587800000000030	F	F	F
0.1929299999999969	0.8496999999999986	0.3587800000000030	F	F	F
0.6567700000000016	0.8055499999999967	0.3587800000000030	F	F	F
0.9382000000000019	0.3322700000000012	0.3522999999999996	F	F	F
0.3940600000000032	0.0602799999999988	0.3522999999999996	F	F	F
0.6662200000000027	0.6044199999999975	0.3522999999999996	F	F	F
0.1635599999999968	0.0629399999999976	0.3486499999999992	F	F	F
0.8993800000000007	0.8349299999999999	0.3486499999999992	F	F	F
0.9355400000000031	0.0991000000000000	0.3486499999999992	F	F	F
0.5020999999999987	0.2683699999999973	0.3084800000000030	F	F	F
0.7662699999999987	0.4963800000000020	0.3084800000000030	F	F	F
0.7301100000000034	0.2322100000000020	0.3084800000000030	F	F	F
0.7274499999999975	0.9990400000000008	0.3048300000000026	F	F	F
0.2715900000000033	0.2710300000000032	0.3048300000000026	F	F	F
0.9994399999999999	0.7268899999999974	0.3048300000000026	F	F	F
0.4727299999999985	0.4816100000000034	0.2983499999999992	F	F	F
0.0088900000000010	0.5257599999999982	0.2983499999999992	F	F	F
0.5168699999999973	0.9896000000000029	0.2983499999999992	F	F	F
0.2515599999999978	0.7389399999999995	0.2882999999999996	F	F	F
0.4873800000000017	0.7469200000000029	0.2882999999999996	F	F	F
0.2595400000000012	0.5110999999999990	0.2882999999999996	F	F	F
0.8276758434332905	0.9248835710568457	0.6788012728618895	T	T	T
0.0779037364937025	0.8963356360988755	0.6786875522409659	T	T	T
0.1059226623663960	0.1755425226087368	0.6788301891182869	T	T	T
0.5681839519144205	0.8787173540341513	0.6732373169728803	T	T	T
0.3191732354684049	0.4351094199108161	0.6730998410363185	T	T	T
0.1243662203052551	0.6832500376945916	0.6732638875597203	T	T	T

0.8480300508241214	0.1693370987495371	0.6702039032479377	T	T	T
0.8334632246278133	0.6729578331944879	0.6704102165506214	T	T	T
0.3289474070417455	0.1543169090109089	0.6698578092887857	T	T	T
0.1009450640162015	0.4429422995874646	0.6678432011158293	T	T	T
0.5601917908454581	0.6525478948575767	0.6674582024677349	T	T	T
0.3501361093537412	0.9013666956447963	0.6676700192177165	T	T	T
0.6018459466767609	0.4443911754573762	0.6615153268440324	T	T	T
0.5575413654705770	0.1519531090167519	0.6614714906913264	T	T	T
0.8502631964231711	0.4001727461517248	0.6615934371071219	T	T	T
0.7737341320527875	0.0101865110414820	0.5656091000637673	T	T	T
0.9903191495644492	0.7593291669978299	0.5655810471276297	T	T	T
0.2409164849223728	0.2269674892326989	0.5654264303821819	T	T	T
0.7383106578700307	0.2241355206128333	0.5645397972437323	T	T	T
0.7763740155639738	0.5097718637329649	0.5645178432871845	T	T	T
0.4906702853937759	0.2623176801725862	0.5645092575642394	T	T	T
0.0016801743830257	0.9987928936736523	0.5567039154711466	T	T	T
0.5022327667224715	0.5010934313201361	0.5570363962603793	T	T	T
0.4990117864145276	0.9972359721020538	0.5570704674788658	T	T	T
0.0030982282464151	0.4977900404290239	0.5570454714188173	T	T	T
0.2285020428292199	0.4906102009808251	0.5486130637350328	T	T	T
0.5096679187930775	0.7342579535572185	0.5485145744231090	T	T	T
0.2657689654691922	0.7716141783007870	0.5486357089757661	T	T	T
0.0144867763616148	0.2394209012759987	0.5493872718899908	T	T	T
0.7608684791544960	0.7708060840787638	0.5494334451868639	T	T	T
0.2295162096053502	0.9858889455739472	0.5494450425457060	T	T	T
0.9050099999999972	0.5600299999999976	0.4504999999999981	F	F	F
0.4050099999999972	0.5600299999999976	0.4504999999999981	F	F	F
0.1550099999999972	0.5934700000000035	0.4504999999999981	F	F	F
0.4384599999999992	0.8434700000000035	0.4504999999999981	F	F	F

0.6550099999999972	0.0934700000000035	0.4504999999999981	F	F	F
0.4384599999999992	0.3434700000000035	0.4504999999999981	F	F	F
0.6661599999999979	0.3323200000000028	0.4428500000000000	F	F	F
0.1661599999999979	0.8323200000000028	0.4428500000000000	F	F	F
0.6661599999999979	0.8323200000000028	0.4428500000000000	F	F	F
0.1661599999999979	0.3323200000000028	0.4428500000000000	F	F	F
0.4273099999999985	0.1046199999999970	0.4352099999999979	F	F	F
0.9273099999999985	0.1046199999999970	0.4352099999999979	F	F	F
0.1773099999999985	0.0711700000000022	0.4352099999999979	F	F	F
0.6773099999999985	0.5711700000000022	0.4352099999999979	F	F	F
0.8938599999999965	0.3211700000000022	0.4352099999999979	F	F	F
0.8938599999999965	0.8211700000000022	0.4352099999999979	F	F	F
0.5716800000000006	0.8933600000000013	0.3362100000000012	F	F	F
0.0716800000000006	0.8933600000000013	0.3362100000000012	F	F	F
0.8216800000000006	0.9268000000000001	0.3362100000000012	F	F	F
0.3216800000000006	0.4268000000000001	0.3362100000000012	F	F	F
0.1051199999999994	0.6768000000000001	0.3362100000000012	F	F	F
0.1051199999999994	0.1768000000000001	0.3362100000000012	F	F	F
0.8328300000000013	0.1656499999999994	0.3285600000000031	F	F	F
0.3328300000000013	0.1656499999999994	0.3285600000000031	F	F	F
0.8328300000000013	0.6656499999999994	0.3285600000000031	F	F	F
0.3328300000000013	0.6656499999999994	0.3285600000000031	F	F	F
0.5939800000000020	0.4379500000000007	0.3209200000000010	F	F	F
0.8439800000000020	0.4045100000000019	0.3209200000000010	F	F	F
0.5605300000000000	0.1545100000000019	0.3209200000000010	F	F	F
0.0939800000000020	0.4379500000000007	0.3209200000000010	F	F	F
0.3439800000000020	0.9045100000000019	0.3209200000000010	F	F	F
0.5605300000000000	0.6545100000000019	0.3209200000000010	F	F	F
0.3373567299464355	0.6657123119883011	0.6676680595729039	T	T	T

References

(1) Sterk, E. B.; Nieuwelink, A. E.; Monai, M.; Louwen, J. N.; Vogt, E. T. C.; Filot, I. A. W.; Weckhuysen, B. M. Structure Sensitivity of CO2Conversion over Nickel Metal Nanoparticles Explained by Micro-Kinetics Simulations. *J. Am. Chem. Soc.* **2022**.

(2) Frei, M. S.; Capdevila-Cortada, M.; García-Muelas, R.; Mondelli, C.; López, N.; Stewart, J. A.; Curulla Ferré, D.; Pérez-Ramírez, J. Mechanism and Microkinetics of Methanol Synthesis via CO2 Hydrogenation on Indium Oxide. *J. Catal.* **2018**, *361*, 313–321.
